# Polysiloxanes Grafted with Mono(alkenyl)Silsesquioxanes—Particular Concept for Their Connection †

**DOI:** 10.3390/ma13214784

**Published:** 2020-10-26

**Authors:** Katarzyna Mituła, Julia Duszczak, Monika Rzonsowska, Patrycja Żak, Beata Dudziec

**Affiliations:** 1 Faculty of Chemistry, Department of Organometallic Chemistry, Adam Mickiewicz University in Poznan, Uniwersytetu Poznanskiego 8, 61-614 Poznan, Poland; julia.duszczak@amu.edu.pl (J.D.); m.rzonsowska@gmail.com (M.R.); pkw@amu.edu.pl (P.Ż.); 2 Centre for Advanced Technologies, Adam Mickiewicz University in Poznan, Uniwersytetu Poznanskiego 10, 61-614 Poznan, Poland

**Keywords:** polymers, hybrid materials, alkenylsilsesquioxanes, silsesquioxanes, polysiloxanes, hydrosilylation, grafting

## Abstract

Herein, a facile and efficient synthetic route to unique hybrid materials containing polysiloxanes and mono(alkyl)silsesquioxanes as their pendant modifiers (T_8_@PS) was demonstrated. The idea of this work was to apply the hydrosilylation reaction as a tool for the efficient and selective attachment of mono(alkenyl)substituted silsesquioxanes (differing in the alkenyl chain length, from -vinyl to -dec-9-enyl and types of inert groups iBu, Ph at the inorganic core) onto two polysiloxanes containing various amount of Si-H units. The synthetic protocol, determined and confirmed by FT-IR in situ and NMR analyses, was optimized to ensure complete Si-H consumption along with the avoidance of side-products. A series of 20 new compounds with high yields and complete β-addition selectivity was obtained and characterized by spectroscopic methods.

## 1. Introduction

Due to their hybrid nature, organosilicon compounds may be applied in the development of advanced, multifunctional materials, owing to a combination of organic and inorganic segments. A fundamental example of such derivatives based on Si-O-Si linkages is polysiloxanes, which are the most widely recognized and studied organosilicon polymers. This is a large group of compounds with a variety of architectures, and linear polymers are one of them. Another example is polysilsesquioxanes which may be classified as polysiloxanes but consist of a general structural unit [RSiO_3/2_]_n_ and contain three main subgroups: amorphous, cage-type and ladder-structured polysilsesquioxanes. The cage-type polysilsesquioxanes derivatives, commonly known as silsesquioxanes (SQs), for their three-dimensional nanosized structures, have attracted considerable attention not only from the synthetic perspective (tunable possibility for modification), but also for their application feature (thermal stability) [1,2,3,4]. These systems, thanks to their unique properties, have influenced almost all branches of science and have found numerous examples of applications also in everyday life, e.g., optoelectronics (OLEDs) [5,6,7], dendrimers [8,9,10,11], catalysts [12,13], medicine and biochemistry (drug delivery systems, dental applications) [14,15,16], lithium and fuel batteries, and conductive matrices [17,18,19,20], food industries, cosmetics, and many more [21,22,23,24,25,26,27,28,29,30,31].

**Figure 1:**
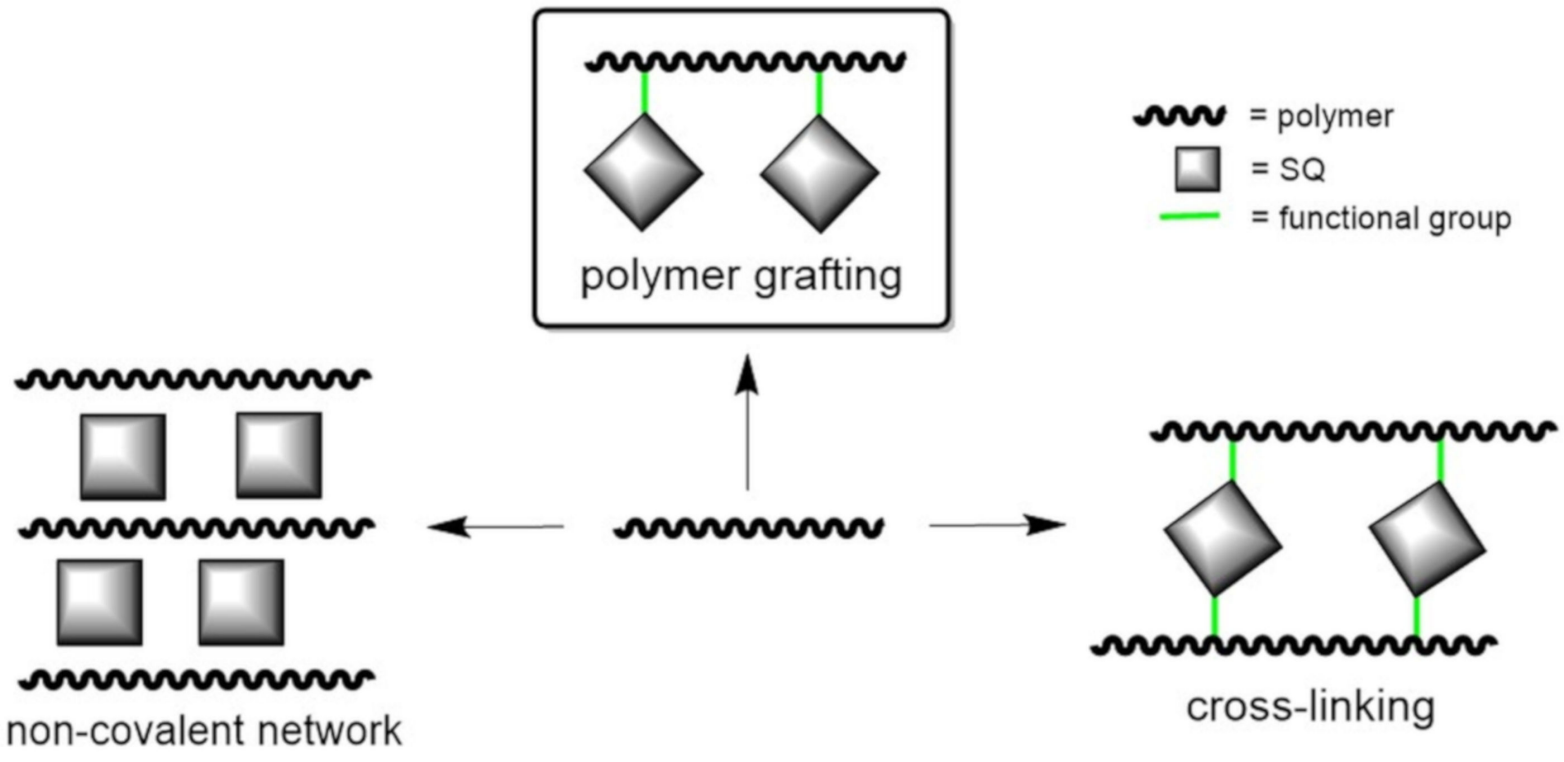
Possible polymer–SQ architectures.

The siloxane bond (Si-O-Si) in polysiloxanes is longer than the classical C-C-C bond in saturated hydrocarbons and is characterized by average bond angle in a range of 110°–143° Si-O-Si vs. the 109.5° angle in C-C-C bond. Because of this feature, the organic substituents at the siloxane skeleton in polysiloxanes are at a greater distance than in hydrocarbons which in turn enables their better rotational mobility affecting, i.a., the elasticity of polysiloxanes [30]. The significant perspectives for polysiloxanes concern modification of their structure. It may be performed at the stage of polysiloxanes chain formation, i.a., anionic ring-opening polymerization of cyclic siloxanes, or hydrolytic polycondensation of two chloro/alkoxysilanes varying in functional groups [23,31,32,33]. On the other hand, it may be achieved by modification of functional group present on the existing polysiloxane chain, mostly via catalytic reactions [34,35,36,37,38,39,40,41,42,43,44], to gain co-polymers with precise and tailored properties [45,46,47,48,49,50,51,52,53,54,55]. Silsesquioxanes represent a versatile group of organosilicon species, with distinct and defined structures, but the most popular are cage silsesquioxanes, especially cubic T_8_ [56,57]. Rigid inorganic, Si-O-Si based core with organic substituents (reactive or inert) coordinated around the silicon vertices, determine properties and potential application of these molecules [58,59,60,61,62,63,64]. The most significant branch of SQs successful application is material chemistry as nanofillers and polymers modifiers [65,66,67,68,69,70,71]. Depending on the type of applied silsesquioxane and its amount in a polymer matrix, the resulting nanocomposite material possesses improved properties, i.e., mechanical (increased elongation at break, lower friction), optical (maintained optical transparency, reduced or removed the color of the nanocomposite), thermal (increased thermal resistance and glass transition temperature, lowered thermal conductivity), and others (e.g., improved the oxidation and corrosion resistance, reduced flammability of the material) [72,73,74,75,76,77,78,79,80,81,82,83,84,85,86]. There are a few possibilities to introduce silsesquioxane molecules into the polymer matrix (Figure 1). The synthetic protocol depends on the amount and type of the reactive groups attached to the SQ core [56,87]. Silsesquioxanes with only inert groups may be included into polymer matrix by simple blending (non-covalent network). On the contrary, when SQs possess several functional groups they may act as cross-linking agents (at the copolymerization step or post-modification of polymer). Finally, silsesquioxanes may be incorporated into the polymer matrix as pendant groups (polymer grafting) at the post-modification stage of an already existing polymer chain, which was exploited in this work. 

It should be emphasized that the reports on the modification of polysiloxanes with silsesquioxanes are limited. These papers concern mostly octa-functionalized T_8_ silsesquioxanes that are used in the modification of polysiloxanes in cross-linking processes resulting in the formation of aerogels, membranes, ceramic precursors or silicone rubbers [88,89,90,91,92,93]. There are also few examples of exploitation of other types of silsesquioxanes. Kuo et al. obtained highly thermally stable and flexible polybenzoxazine nanocomposites after hydrosilylation of di-functional double-decker (DDSQ) silsesquioxane with polydimethylsiloxane [94]. Kunitake et al. used a polycondensation reaction to obtain dimethylsiloxane polymers with embedded DDSQ cages of alternating chains length [95]. Naka et al. utilized incomplete-condensed silsesquioxane as monomers in platinum-catalyzed hydrosilylation polymerization with siloxane to obtain polysiloxanes with SQs in the main chain [96]. He et al. obtained organic/inorganic hybrids for coating by polymerization of modified mono-carbinol terminated polymethylsiloxane with mono(methacryl)isobutyl silsesquioxane (9.48, 17.33 and 23.93 wt % SQ content) [97]. Lee et al. applied mono(allyl)cyclohexyl silsesquioxane for poly(ethylhydrosiloxane) grafting via hydrosilylation reaction in the presence of Karstedt’s catalyst [98,99]. They used up to 25 mol % of SQ per Si–H group in polysiloxane (to ensure 100% conversion of all Si–H group in polysiloxane they performed additional reaction with 1-octene or allyl-oligo(ethylene oxide)). The obtained materials exhibited better mechanical and thermal property than the parent polymer matrix with increasing content of SQ. 

**Figure 2:**
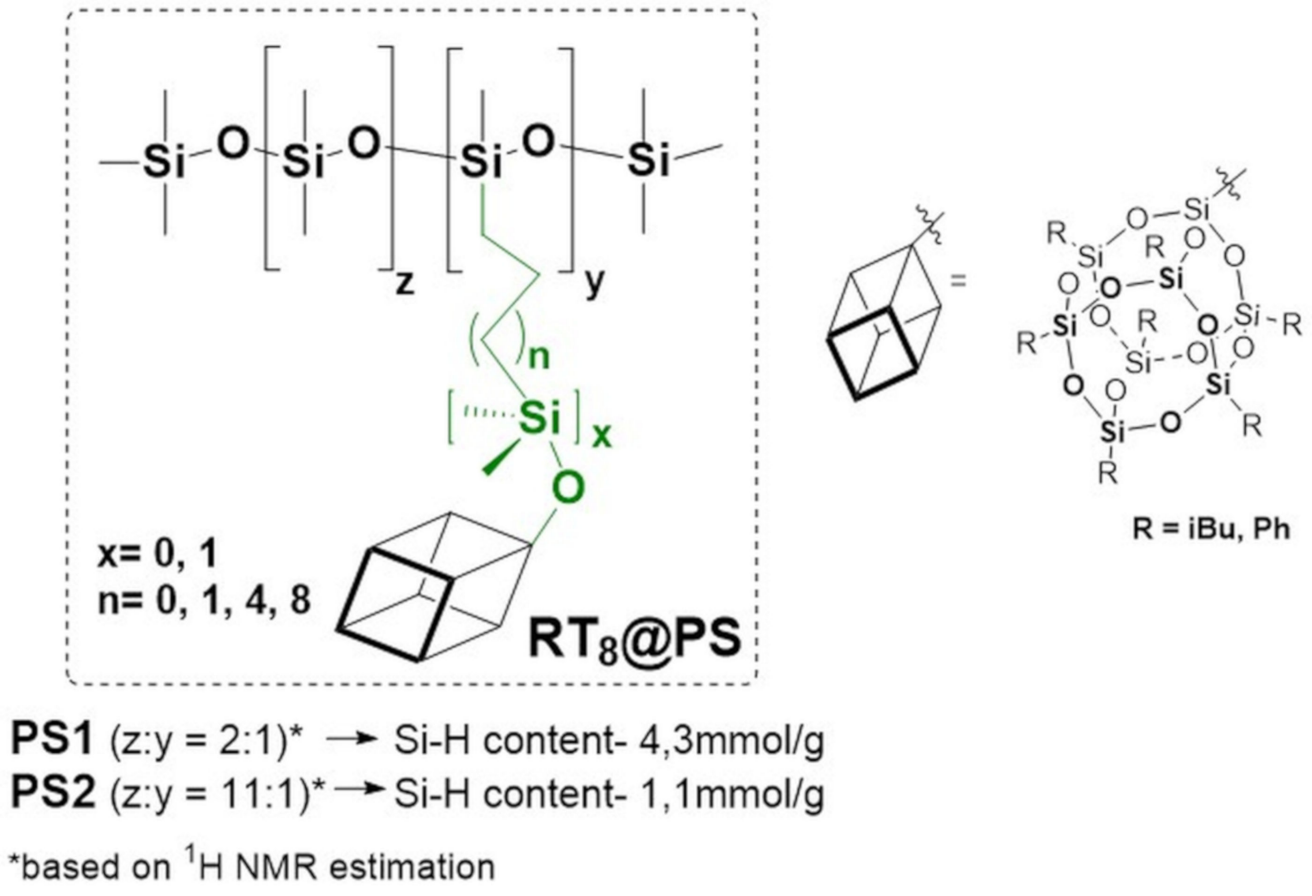
Schematic depiction of Polysiloxanes grafted with Mono(alkenyl)Silsesquioxanes RT_8_@PS.

The idea of our research was to connect the polysiloxanes (PDMHS) and silsesquioxanes (SQs) unique structures into one, resulting in the formation of hybrid, polymeric SQ-based systems. Hence, a study was undertaken to design new types of hybrid materials that are based on polysiloxanes and different silsesquioxanes as their modifiers and to provide an efficient procedure for their synthesis. The idea of this research was to use Poly(dimethylsiloxane-co-methylhydrosiloxane) (PDMHS) with reactive Si-H moiety as a hydrosilylating agent of mono(alkenyl)substituted T_8_ SQs. The crucial aspect of this study was to investigate the possibility to use polysiloxanes varying in Si-H content (PS1-PS2) and mono(alkenyl)substituted T_8_ SQs with different alkenyl chain lengths to achieve the complete conversion of reactive groups of PDMHS. It was monitored with FTIR in situ apparatus (Figure 2). 

Moreover, as the hydrosilylation reaction may be accompanied by side-reactions, such as dehydrogenative silylation, isomerization or olefin’s hydrogenation, additional studies were undertaken to resolve this aspect using diverse Pt-based catalytic systems [100]. The final products, i.e., the polysiloxanes grafted with mono(alkenyl)silsesquioxanes T_8_@PS were obtained as air-stable organosilicons systems with diverse structures and a morphology dependent on the amount of the SQ attached to the polysiloxane chain as well as the length of the alkyl linker between them.

## 2. Materials and Methods 

### 2.1. Materials

The chemicals were purchased from the following sources: Karstedt’s catalyst (platinum(0)-1,3-divinyl-1,1,3,3-tetramethyldisiloxane complex (Pt_2_(dvs)_3_) solution in xylene with 2% of Pt), calcium hydride, platinum(IV) oxide (surface area ≥ 75 m^2^/g) from Sigma-Aldrich (Saint Louis, MO, USA). Sodium and benzophenone from Acros Organics (Geel, Belgium). Polysiloxanes: Cross-linker 101 (PS1, 4.3 mmol/g Si–H content) and 120 (PS2, 1.1 mmol/g Si-H content) from Evonik (Darmstadt, Germany). ((IPr*)Pt(dvds)) (IPr* = 1,3-bis{2,6-bis(diphenylmethyl)-4-methylphenyl}-imidazol-2-ylidene) and Mono(alkenyl)functionalized silsesquioxanes were synthesized according to the literature [101,102]. Toluene, tetrahydrofuran (THF), chloroform-d, molecular sieves type 4 Å from Chempur (Piekary Śląskie, Poland). Argon and liquid nitrogen were obtained from Linde Gas (Kraków, Poland). All syntheses were conducted under argon atmosphere using standard Schlenk-line and vacuum techniques. Toluene was dried over CaH_2_ and THF over Na with benzophenone prior to use and stored under argon over type 4 Å molecular sieves. 

### 2.2. Measurements

Nuclear magnetic resonance spectroscopy (NMR) measurements (^1^H, ^13^C, and ^29^Si NMR) were conducted using spectrometers: Bruker Ultrashield 300 MHz and 400 MHz respectively (Faellanden, Switzerland) with CDCl_3_ as a solvent. Chemical shifts are reported in ppm with reference to the residual solvent signals (CHCl_3_) peaks for ^1^H and ^13^C and to TMS for ^29^Si.

Fourier transform-infrared (FT-IR) spectra were recorded on a Nicolet iS5 (Thermo Scientific, Waltham, MA, USA) spectrophotometer equipped with a SPECAC Golden Gate, diamond ATR unit with a resolution of 2 cm^−1^. In all cases, 16 scans were collected to record the spectra in a range of 4000–430 cm^−1^.

Real-time in situ FT-IR measurements were performed on a Mettler Toledo ReactIR 15 (Mettler-Toledo GmbH, Greifensee, Switzerland) equipped with a DS 6.3 mm AgXDiComp Fiber Probe with a diamond sensor, and a Mercury Cadmium Telluride detector. For all spectra, 256 scans were recorded with the resolution of 1 cm^−1^ in 1, 2, 5, and 10 min. intervals.

### 2.3. Synthetic Procedures

General Procedure for grafting of PDMHS with SQs via hydrosilylation reaction (1-5-iBuT_8_@PS1-2 and 1-5-PhT_8_@PS1-2 synthesis)

The procedure for the synthesis of 2-iBuT_8_@PS1 is described as an example.

2-iBuT_8_ (538 mg, 0.645 mmol), PS1 (150 mg, 0.645 mmol) and anhydrous toluene (3 mL) were introduced into Schlenk reactor purged with argon, equipped with a magnetic stirrer and in situ FT-IR probe. The reaction mixture was heated up to 90 °C and afterwards (Pt_2_(dvds)_3_) (Karstedt’s catalyst 2% solution in xylene, 0.736 μL, 6.45 × 10^−8^ mol) was added. Reactions were carried out until >99% (or maximum) conversion of reactive Si-H in PS1, which was precisely controlled by in situ FT-IR apparatus analyzing the disappearance of Si-H bands at ῡ = 900 cm^−1^ (24–96 h). After cooling it to room temperature solvent was evaporated under a vacuum.

## 3. Results and Discussion

In our previous studies, we reported on an efficient synthetic protocol for the preparation of a library of mono(alkenyl)silsesquioxanes varying in the length of the alkenyl chain and also in the inert substituents (R = Et, iBu, Cy, iOc, Ph) at the silsesquioxane core [101]. These studies revealed the dependence of the thermal stability of this series of compounds on the type of inert (R) and alkenyl group at SQs as well as the presence of the –OSi(Me_2_)– spacer. In this paper, our goal was to synthesize a series of hybrid materials based on selected PDMHS-Poly(dimethylsiloxane-co-methylhydrosiloxane)–PS1 (with 4.3 mmol/g Si-H content) and PS2 (with 1.1 mmol/g Si-H content) grafted with mono(alkenyl)substituted T_8_ SQs as pendant groups. Basing on the scientific literature and our experience, we selected two types of mono(alkenyl)substituted T_8_ SQs possessing two distinct inert substituents, i.e., iBu and Ph that exhibit different solubility as well as thermal stability [101]. It is well known that the type of inert and functional groups determine the physical and chemical properties of silsesquioxane [56,60,87] and as a consequence, may influence the properties of polysiloxanes modified with these compounds.

**Scheme 1:**
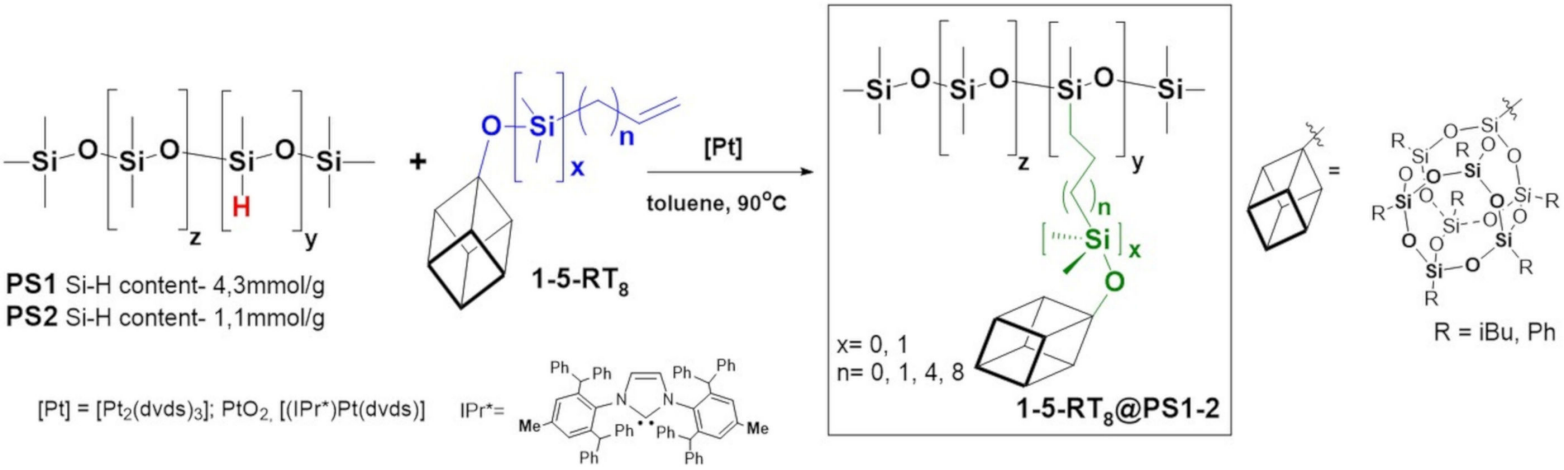
General scheme for the hydrosilylation of mono(alkenyl)functionalized silsesquioxanes (1-5RT_8_) with Poly(dimethylsiloxane-co-methylhydrosiloxane) (PS1-2).

The synthetic protocol was based on the general reaction presented in Scheme 1. Preliminary tests were performed to optimize the reaction conditions in terms of the catalyst type and its amount. The Pt-based catalytic systems, i.e., (Pt_2_(dvds)_3_), PtO_2_ and (Pt(IPr*)(dvds)) (IPr* = 1,3-bis{2,6-bis(diphenylmethyl)-4-methylphenyl}-imidazol-2-ylidene) were selected due to their confirmed activity in the hydrosilylation reaction [52,103,104,105,106,107]. The selection of toluene was based on the good solubility of all 1-5-RT_8_ SQs as well as PS1-2.

**Figure 3:**
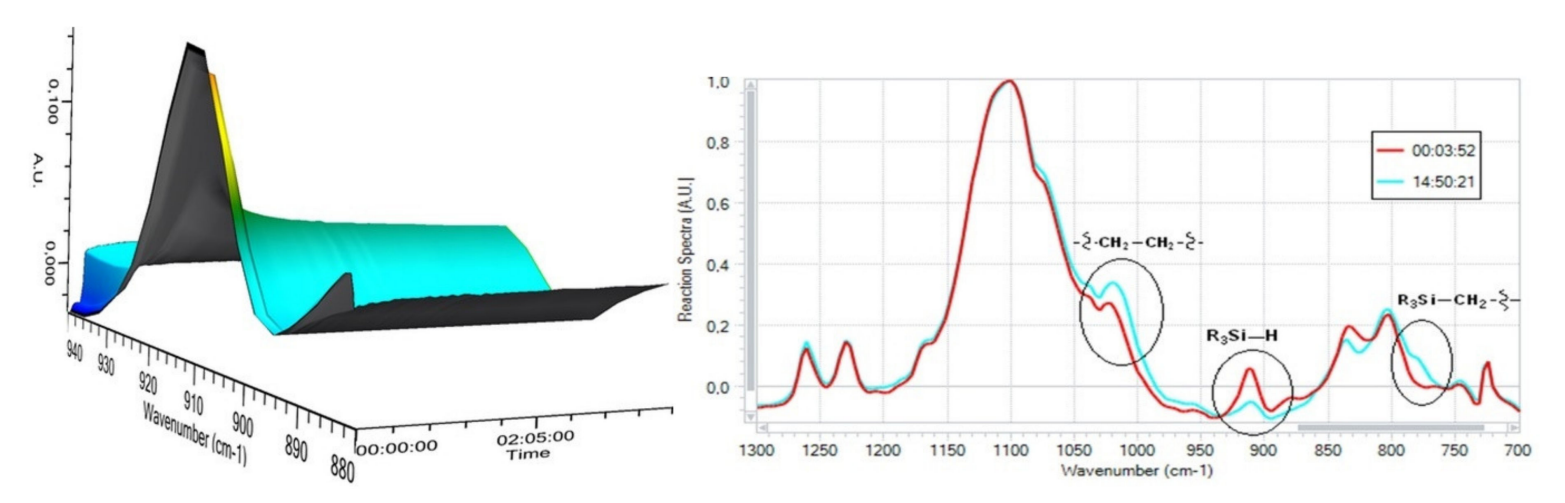
Selected FT-IR spectrum area—illustration of Si-H band disappearance at ῡ = 907 cm^−1^, recorded in real-time during hydrosilylation of 2-iBuT_8_ with PS1.

The progress of the reaction was performed using FT-IR Mettler Toledo ReactIR 15 (Mettler-Toledo GmbH, Greifensee, Switzerland) apparatus that provided on-line (in situ) monitoring reaction course, due to the changes in the area of the bands ascribed to the stretching vibrations of Si–H bond within time, i.e., at ῡ = 907 cm^−1^ (Figure 3). The final conversions of reagents we also confirmed by the ^1^H NMR.

The reagents and catalyst stoichiometry was verified to ensure the highest Si-H conversion. This is dependent on several factors, i.e., the type of inert R group at silsesquioxane (iBu, Ph), alkenyl chain length and the presence/absence of siloxane (–OSiMe_2_-) spacer between the SQ core and alkenyl chain. The optimized catalyst amount was established at 10^−4^ mol of (Pt_2_(dvds)_3_) for 1-5-iBuT_8_ (Table 1) and 10^−3^ mol of (Pt_2_(dvds)_3_) for 1-5PhT_8_ (Table 2) per one mol of Si-H in PS1-2. A higher amount of catalyst necessary for 1-5-PhT_8_ with phenyl inert groups might be related to the bulkiness of the SQs core affecting the limited access of 1-5-PhT_8_ to the reactive Si-H bond in PS1-2. Additionally, their electronic impact may also be important.

In the case of vinyl moiety attached directly to the SQs core, i.e., the substrate without the siloxane (-OSiMe_2_-) spacer (1-iBuT_8_) requires higher amount of catalyst, i.e., 10^−3^ mol of (Pt_2_(dvds)_3_) to ensure full conversion of Si-H (Table 1, entries 2 and 3). It is also the steric aspect of the reagent. When 10^−4^ mol of Karstedt’s catalyst was exploited, only 50% of Si-H conversion was noted, as well as even longer, i.e., 96 h of reaction time. It might be also the aspect of steric hindrance and closure of the SQ core preventing the effective addition of vinyl moiety into the coordination sphere of Pt (according to the Chalk–Harrod mechanism) [100]. On the contrary, the hydrosilylation of 4-PhT_8_ and 5-PhT_8_ might also be conducted with lower loading of the catalyst, i.e., 10^−4^ mol of (Pt_2_(dvds)_3_) (Table 2, entries 9 and 12), but it affects the total reaction time and an additional 72 h apart from 24 h which are required for complete Si-H conversion.

The inert groups and type of alkenyl chain also impact the reaction time. The highest conversion of PS1-2 grafted with iBuT_8_ was ensured within 24 h for almost all alkenyl chain lengths and for Karstedt’s catalyst used. The exceptions are hydrosilylation reactions of PS1 and PS2 with 1-iBuT_8_ as the time evaluated for these reactions was 48 h. This might be explained with the absence of a siloxane (-OSiMe_2_-) spacer between -vinyl group and SQ core. On the contrary, the reaction time of polysiloxanes grafting with PhT_8_ significantly depends on alkenyl chain length. For -vinyl and -allyl groups the evaluated time was 48 h in comparison to -hex-5-enyl and -dec-9-enyl groups as in their case the established time was 24 h (both for Karstedt’s catalyst). Longer alkenyl chains are further apart from the SQ core and bulky inert groups (Ph), which in consequence enables their better access to Si-H bond. 

**Scheme 2:**
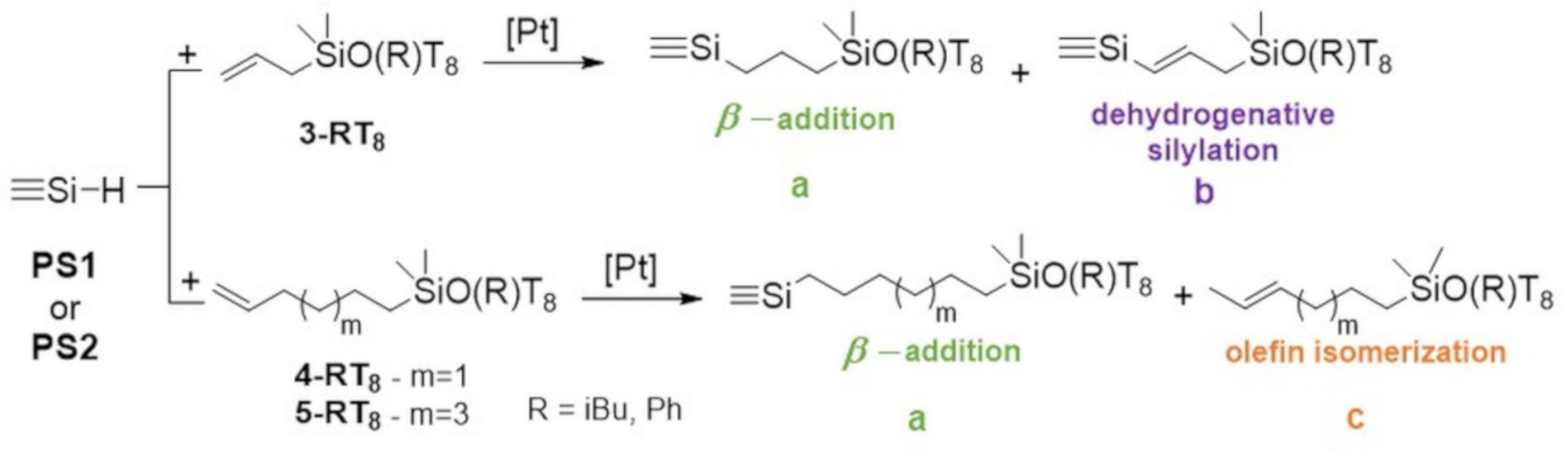
A route for the hydrosilylation of 3-5-RT_8_ by PS1 and PS2 and possible products in the reaction mixtures.

**Figure 4:**
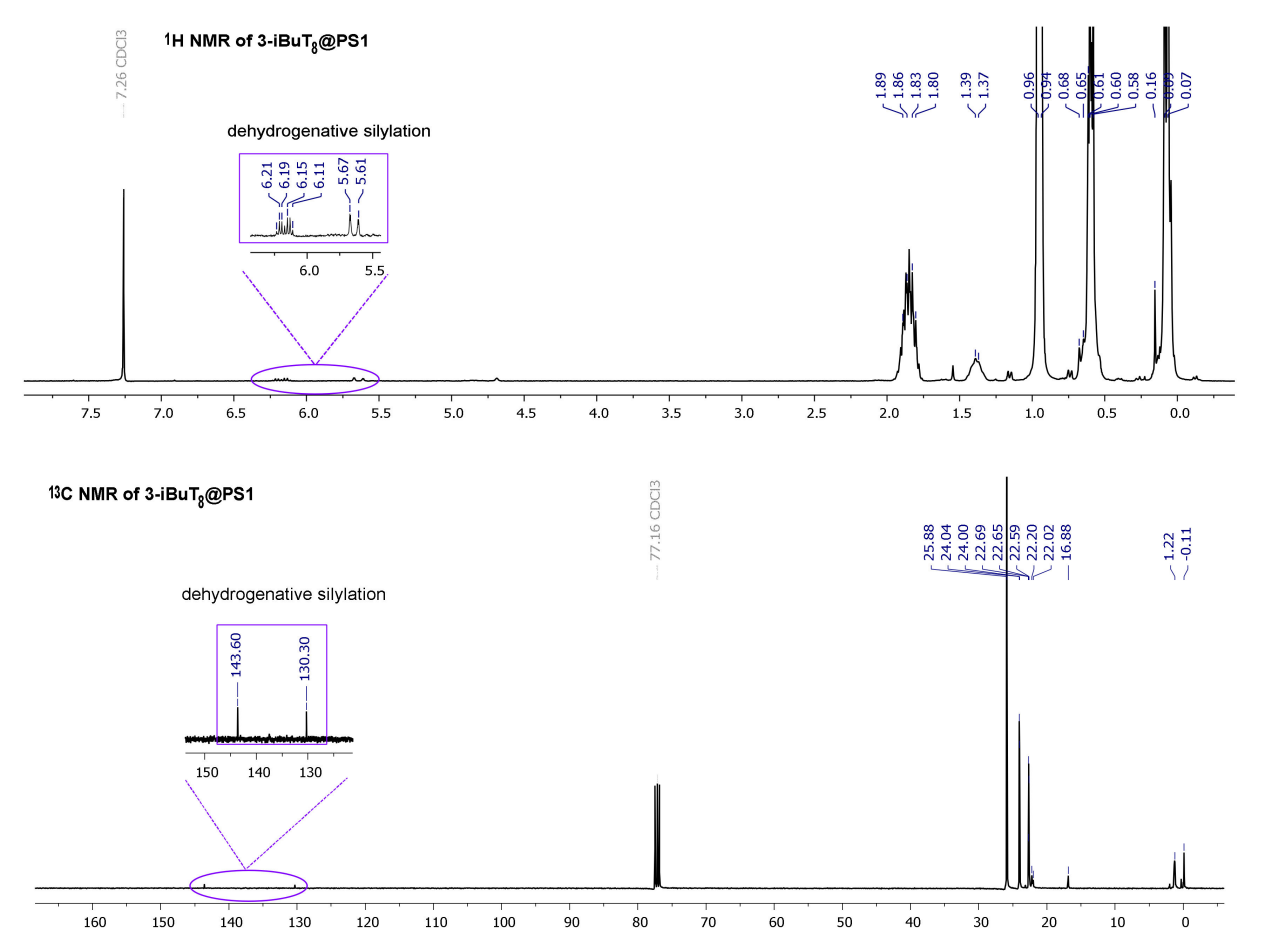
^1^H and ^13^C NMR spectra of 3-iBuT_8_@PS1 after hydrosilylation reaction.

**Figure 5:**
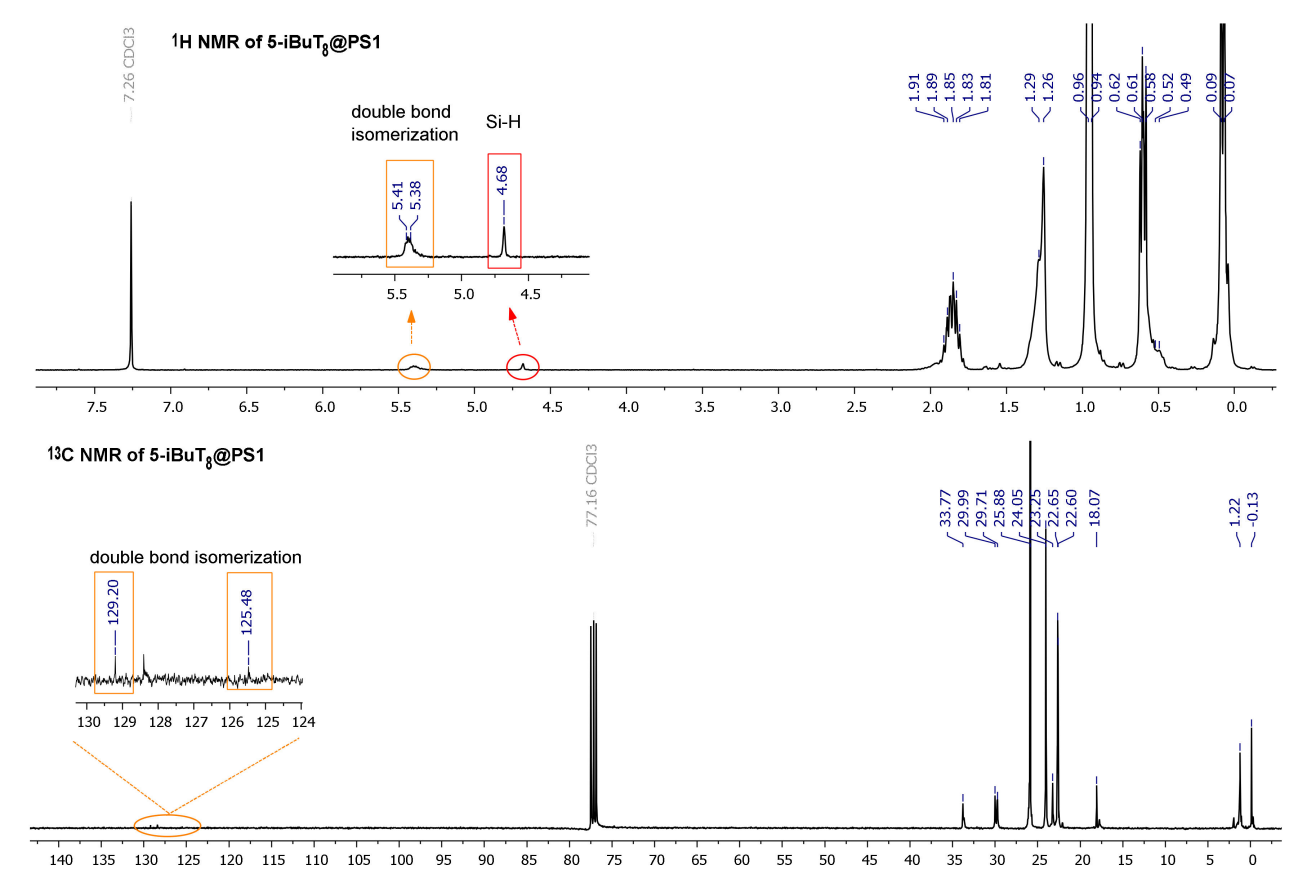
^1^H and ^13^C NMR spectra’s of 5-iBuT_8_@PS1 after hydrosilylation reaction.

It is worth mentioning that hydrosilylation may not proceed with a complete selectivity control towards the desired product, i.e., in this system, β-addition product and side reactions may occur, e.g., isomerization of olefin, α-addition product or dehydrogenative silylation [100,108,109]. For the reactions with SQs with an alkenyl chain longer than -vinyl, i.e., for -allyl, -hex-5-enyl and -dec-9-enyl derivatives (3-5RT_8_), a careful evaluation of spectroscopic analyses (Figure 4 and Figure 5) revealed the presence of by-products (Scheme 2). In the case of -allyl 3-iBu/PhT_8_, the formation of dehydrogenative silylation by-product (Scheme 2–product b) was noted. Interestingly, the Pt-based complexes are not particularly favorable towards the dehydrogenative silylation [110,111] in contrast to 8–9 group of the periodic table, i.e., iron [112], rhodium [113], iridium [114] and cobalt [115] complexes, specifically known for this selectivity [116,117]. This observation is reflected in NMR spectra (Figure 4) and the appearance of new signals in ^1^H NMR derived from –C*H*=C*H*-Si-bond, i.e., 5.61–5.67, 6.11–6.23 ppm and in ^13^C NMR: 130.30, 143.60 ppm. They are shifted in comparison to the signals of the terminal –CH=CH_2_ bond in the -allyl group (^1^H NMR: 4.84–4.91, 5.73–5.87 ppm and ^13^C NMR: 113.54, 133.99 ppm respectively). However, for the -hex-5-enyl, i.e., 4-iBu/PhT_8_ and -dec-9-enyl, i.e., 5-iBu/PhT_8_, the by-product of double bond isomerization in alkenyl moiety (Scheme 2–product c) was noted. The analogical observation was previously reported for the hydrosilylation of 1,5-hexadiene by chlorosilane by Saiki et al. [118]. In our case, the recorded ^1^H NMR spectrum revealed (Figure 5) the appearance of new signals at 5.38–5.41 ppm and respective resonance lines: 125.48, 129.20 ppm were also present at ^13^C NMR. They were assigned to the –C*H*=C*H*-CH_3_ moiety derived from double bond isomerization in the alkenyl group attached to the SQs core. Moreover, since the isomerization of C=C bond in the 4-5-RT_8_ is noted and these molecules are inactive in the hydrosilylation conditions, there is a characteristic peak at 4.68 ppm in ^1^H NMR spectrum (Figure 5). This confirms the incomplete conversion of Si-H due to the formation of a by-product (bond isomerization, Scheme 2–product c) of hydrosilylation reaction.

For that reason, additional tests for grafting of the PS1 with SQs possessing longer alkenyl chains, i.e., -allyl (Table 1–entry 8, 9-3a,b-iBuT_8_@PS1 and Table 2–entry 7, 8-3a,b-PhT_8_@PS1) and -dec-9-enyl (Table 1–entry 14, 15-5a,b-iBuT_8_@PS1 and Table 2–entry 15, 16-5a,b-PhT_8_@PS1), with different platinum catalysts, i.e., ((IPr*)Pt(dvds)) and PtO_2_ were performed. It was decided to use 10^−4^ mol of ((IPr*)Pt(dvds)) [105] and 10^−2^ mol of PtO_2_ [119,120,121] per 1 mol of Si-H group based on the literature. The estimated time for the hydrosilylation of 3-iBu/PhT_8_ (72 h vs. 24 and 48 h) and 5-iBu/PhT_8_ (48 h vs. 24 h) was significantly higher in comparison with [Pt_2_(dvds)_3_]. Moreover, these reaction times did not ensure the complete conversion of Si-H bond. The selectivity of the process was also not improved (Table 1 entry 8, 9 and 14–15; Table 2, entry 7–8 and 15–16). The NMR spectra of the reactions mixtures resulting from these tests revealed the presence of by-products i.e., dehydrogenative silylation (for 3-iBu/PhT_8_) and double bond isomerization (for 5-iBu/PhT_8_). Finally, additional tests verifying the influence of reaction mixture concentration on the by-product formation were performed. The hydrosilylation reaction of the selected 3-PhT_8_ by PS1 was performed in higher (0.17 M) and lower (0.022 M) concentrations than set 0.057 M. However, equally for both tests, a product of dehydrogenative silylation was observed in the post-reaction mixture (^1^H NMR).

**Figure 6:**
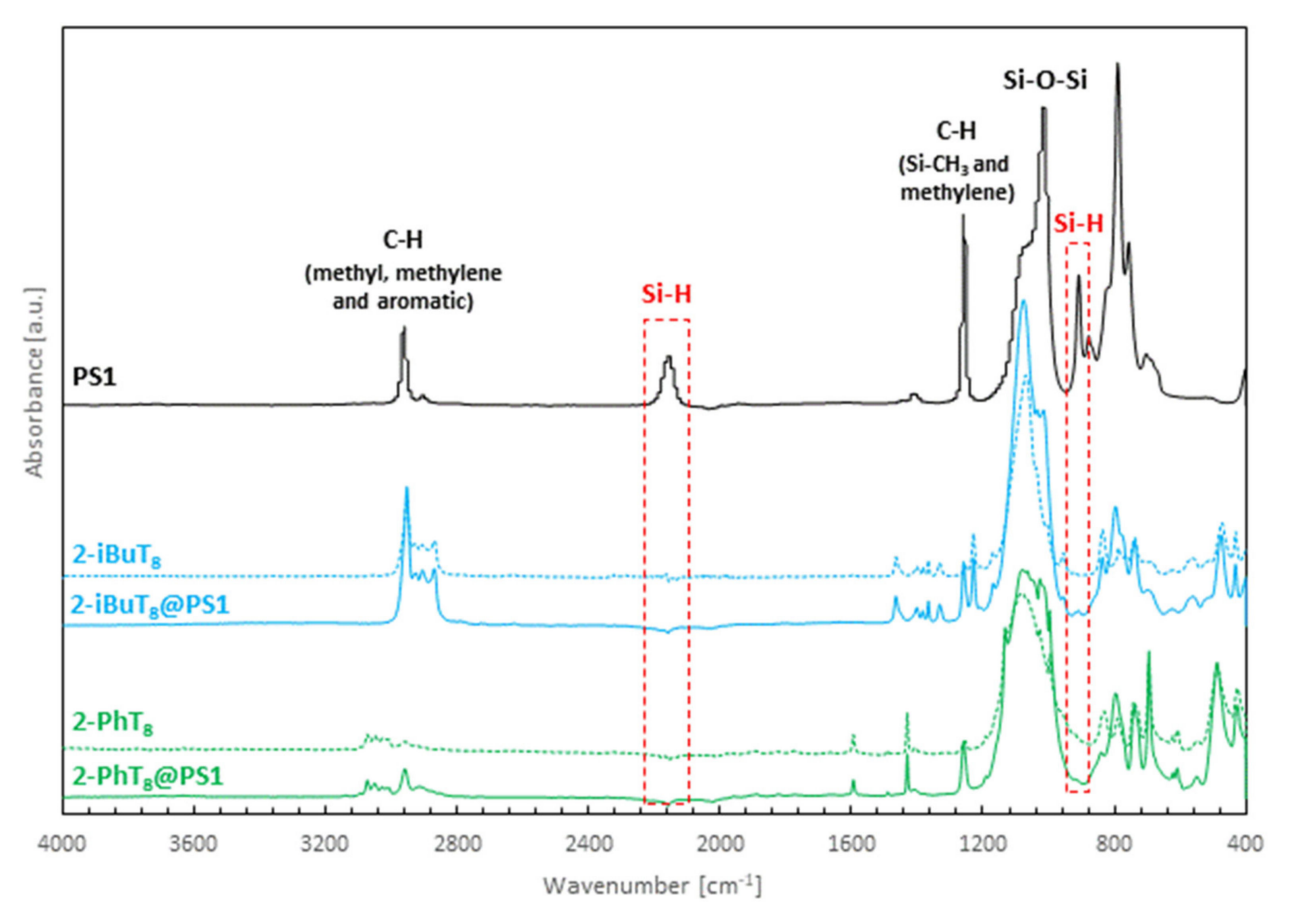
Selected FT-IR spectra of polysiloxane PS1 and mono(vinyl)substituted iBuT_8_ (2-iBuT_8_), and mono(vinyl)substituted PhT_8_ (2-PhT_8_) along with the obtained products 2-iBu/PhT_8_@PS1.

The stacked FT-IR spectra of starting material (PS1-2), selected mono(alkenyl)silsesquioxane, i.e., 2-iBuT_8_ and 2-PhT_8_ substrates and the resulting products: (2-iBu/PhT_8_@PS1-2) are depicted in Figure 6 and Figure S1 (see ESI). Established reaction conditions resulted in the complete conversion of reactive Si-H groups in PS1 and PS2, confirmed by the disappearance of respective band at ῡ = 895–900 cm^−1^ and ῡ = 2130–2157 cm^−1^, marked in Figure 6 and Supplementary Figure S1.

For the respectively modified polysiloxanes, i.e., 2-iBuT_8_@PS1, 2-iBuT_8_@PS2, 2-PhT_8_@PS1 and 2-PhT_8_@PS2 (in Supplementary Figure S1), there are new bands on the spectra, characteristics of C-H (stretching vibrations) methyl group from polysiloxane, as well as iBu moiety methylene groups in the aliphatic chain at a range, ca. 2962–2870 cm^−1^ and additional bands at a range ca. 1259–1229 cm^−1^ attributed to the bending vibrations (doubled in the case of 2-iBuT_8_@PS1, 2-iBuT_8_@PS2) of the aforementioned C-H groups. There are also aromatic C-H stretching bands at ca. 3074–3029 cm^−1^ and C=C (aromatic) at ca. 1594 and 1430 cm^−1^ characteristic of phenyl groups presence in the case of 2-PhT_8_@PS1 and 2-PhT_8_@PS2. These FT-IR spectra confirm the formation of the desired grafted polysiloxanes. 

**Figure 7:**
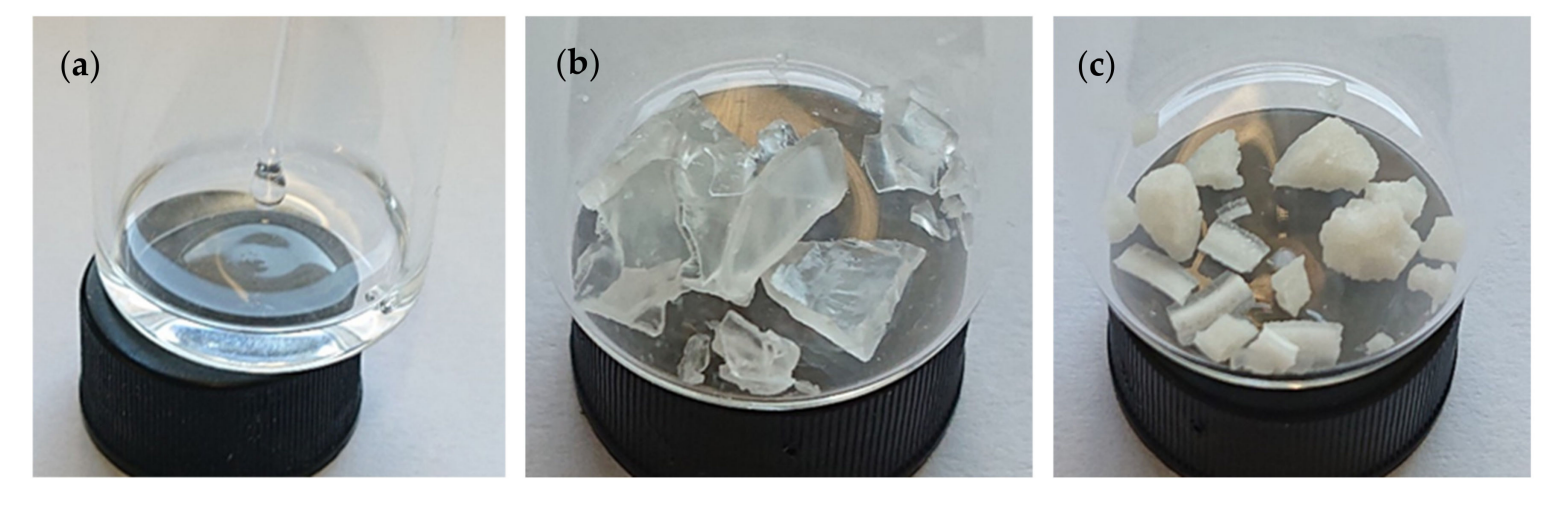
The images of the PS1 (**a**) and selected final, grafted polymers: 2-iBuT_8_@PS1 (**b**) and 5-PhT_8_@PS1 (**c**).

The crude products were isolated from the reaction mixture by evaporation of the solvent on a vacuum. All obtained products are air-stable solids or waxy solids and can be synthesized on a multigram scale. They are soluble in organic solvents like DCM, CHCl_3_, THF, acetone, and toluene, but not in, e.g., methanol, MeCN, and hexane (for products with Ph groups). They were isolated (Table S1) and characterized by means of spectroscopic (^1^H, ^13^C and ^29^Si NMR as well as FT-IR, see Supplementary Materials) methods. Depending on the content of Si-H in the PS1 and PS2, as well as the alkyl chain via which the SQs core is attached to the polysiloxane, and finally on the presence of iBu or Ph inert substituents, the resulting 1-5RT_8_@PS1-2 differ in the morphology (Figure 7).

## 4. Conclusions

To conclude, we designed a synthetic pathway for an efficient PDMHS modification with selected alkenylfunctionalized SQs (the alkenyl group with a different chain length, from -vinyl to -dec-9-enyl and two types of inert groups- iBu, Ph at the inorganic core). We revealed a simple and efficient route to graft the polysiloxanes with the abovementioned RT_8_, based on Pt-catalyzed hydrosilylation reaction. The synthetic protocol was optimized to ensure a complete Si-H consumption along with the avoidance of side-products. As a result, we obtained 20 new compounds, characterized by spectroscopic methods (^1^H, ^13^C, ^29^Si NMR, and FT IR; see Supporting Information). These compounds not previously been described. Based on the literature concerning the directions of polysiloxanes application, these materials seem to be potentially interesting to be used as protecting coatings. Further studies aiming at the determination of the physical properties (thermal, mechanical, etc.) of these materials are planned to be performed.

## Figures and Tables

**Table 1:** The hydrosilylation reaction of PS1-2 with iBuT_8_^a.^

Entry	RT_8_	PS	[Pt] (Amount per One Si-H Group)	Conversion of Si-H ^b^ [%]	Time [h]	Selectivity (a/b/c) ^d^ [%]	Product
1	R = iBu; x = 0; n = 0 1-iBuT_8_	1	[Pt_2_(dvds)_3_]	>50	96	100/0/0	1′-iBuT_8_@PS1 ^e^
2		1		>99 ^c^	48	100/0/0	1-iBuT_8_@PS1 ^e^
3		2		>99 ^c^	48	100/0/0	1-iBuT_8_@PS2 ^f^
4	R = iBu; x = 1; n = 0 2-iBuT_8_	1		>99	24	100/0/0	2-iBuT_8_@PS1 ^e^
5		2		>99	24	100/0/0	2-iBuT_8_@PS2 ^f^
6	R = iBu; x = 1; n = 1 3-iBuT_8_	1		>94	24	87/13/0	3-iBuT_8_@PS1 ^e^
7		2		>94	24	94/6/0	3-iBuT_8_@PS2 ^f^
8		1	[(IPr*)Pt(dvds)]	>92	72	85/15/0	3a-iBuT_8_@PS1 ^e^
9			PtO_2_	>94	72	88/12/0	3b-iBuT_8_@PS1 ^e^
10	R = iBu; x = 1; n = 4 4-iBuT_8_	1	[Pt_2_(dvds)_3_]	>90	24	94/0/6	4-iBuT_8_@PS1 ^e^
11		2		>87	24	90/0/10	4-iBuT_8_@PS2 ^f^
12	R = iBu; x = 1; n = 8 5-iBuT_8_	1		>89	24	86/0/14	5-iBuT_8_@PS1 ^e^
13		2		>88	24	87/0/13	5-iBuT_8_@PS2 ^f^
14		1	[(IPr*)Pt(dvds)]	>80	48	86/0/14	5a-iBuT_8_@PS1 ^e^
15			PtO_2_	>89	48	87/0/13	5b-iBuT_8_@PS1 ^e^

^a^ Reaction conditions: [HSi]:[1-5-iBuT_8_]:[Pt] = 1:1:10^−4^, for [Pt] = PtO_2_:10^−2^; toluene (0.057 M), 90 °C. ^b^ Conversion of Si-H was determined by FT-IR in situ and ^1^H NMR analyses. ^c^ [Pt_2_(dvds)_3_] = 10^−3^. ^d^ Process selectivity was confirmed by ^1^H, ^13^C and ^29^Si NMR (**a-product of β-addiction; b-product of dehydrogenative silylation; c-product of C=C bond isomerization**). ^e^ State of compound: transparent solid, ^f^ State of compound: transparent viscous solid.

**Table 2:** The hydrosilylation reaction of PS1-2 with PhT_8_^a.^

Entry	SQ	PS	[Pt] (Amount per One Si-H Group)	Conversion of Si-H ^b^ [%]	Time [h]	Selectivity (a/b/c) ^d^ [%]	Product
1	R = Ph; x = 0; n = 0 1-PhT_8_	1	[Pt_2_(dvds)_3_]	>99	48	100/0/0	1-PhT_8_@PS1 ^e^
2		2		>99	48	100/0/0	1-PhT_8_@PS2 ^f^
3	R = Ph x = 1; n = 0 2-PhT_8_	1		>99	48	100/0/0	2-PhT_8_@PS1 ^e^
4		2		>99	48	100/0/0	2-PhT_8_@PS2 ^f^
5	R = Ph; x = 1; n = 1 3-PhT_8_	1		>94	48	89/11/0	3-PhT_8_@PS1 ^e^
6		2		>94	48	81/19/0	3-PhT_8_@PS2 ^f^
7		1	[(IPr*)Pt(dvds)]	>93	72	88/12/0	3a-PhT_8_@PS1 ^e^
8			PtO_2_	>92	72	88/12/0	3b-PhT_8_@PS1 ^e^
9	R = Ph; x = 1; n = 4 4-PhT_8_	1	[Pt_2_(dvds)_3_]	>90 ^c^	96	91/0/9	4′-PhT_8_@PS1 ^e^
10				>91	24	93/0/7	4-PhT_8_@PS1 ^e^
11		2		>86	24	89/0/11	4-PhT_8_@PS2 ^f^
12	R = Ph; x = 1; n = 8 5-PhT_8_	1		>88 ^c^	96	90/0/10	5′-PhT_8_@PS1 ^e^
13				>90	24	89/0/11	5-PhT_8_@PS1 ^e^
14		2		>87	24	89/0/11	5-PhT_8_@PS2 ^f^
15		1	[(IPr*)Pt(dvds)]	>84	48	89/0/11	5a-PhT_8_@PS1 ^e^
16			PtO_2_	>88	48	88/0/12	5b-PhT_8_@PS1 ^e^

^a^ Reaction conditions: [HSi]:[1-5-PhT_8_]:[Pt] = 1:1:10^−3^, for [Pt] = PtO_2_:10^−2^; toluene (0.057 M), 90 °C. ^b^ Conversion of Si-H was determined by FT-IR in situ and ^1^H NMR analyses. ^c^ [Pt_2_(dvds)_3_] = 10^−4^. ^d^ Process selectivity was confirmed by ^1^H, ^13^C and ^29^Si NMR (**a-product of β-addiction; b-product of dehydrogenative silylation; c-product of C=C bond isomerization**). ^e^ State of compound: milky solid, ^f^ State of compound: milky viscous solid.

## Supplementary Materials

Detailed analytical data with NMR spectra of all isolated products along with the additional NMR spectra are available online at https://www.mdpi.com/1996-1944/13/21/4784/s1.
