# Supplementary material for: Polysiloxanes Grafted with Mono(alkenyl)Silsesquioxanes—Particular Concept for Their Connection
Source: Materials (Basel). 2020 Oct 26;13(21):4784. doi: 10.3390/ma13214784 (PMC7662624; doi:10.3390/ma13214784)
Supplement: Supplementary file 1 [file materials-13-04784-s001.pdf]

Supporting Information for

Polysiloxanes grafted with Mono(alkenyl)Silsesquioxanes – Particular Concept for their Connection‡

Katarzyna Mituła[1,2,*], Julia Duszczak[1,2], Monika Rzonsowska[1,2], Patrycja Żak[1], Beata Dudziec[1,2,*]

[1] Faculty of Chemistry, , Department of Organometallic Chemistry, Adam Mickiewicz University in Poznan, Uniwersytetu Poznanskiego 8, 61-614 Poznan, Poland; katarzyna.mitula@gmail.com (K.M.); julia.duszczak@amu.edu.pl (J.D.); m.rzonsowska@gmail.com (M.R); pkw@amu.edu.pl (P.Ż.); beata.dudziec@gmail.com (B.D.)

[2] Centre for Advanced Technologies, Adam Mickiewicz University in Poznan, Uniwersytetu Poznanskiego 10, 61-614 Poznan, Poland;

* Correspondence: katarzyna.mitula@gmail.com (K.M.); beata.dudziec@gmail.com (B.D.); Tel.: +48 618291878

‡Dedicated to Professor Bogdan Marciniec on the occasion of his forthcoming 80th birthday.

Table of contents:

1. Table S1 of compounds ………………………………………………..………………. ••• S 2--

2. Data characterizing PS1-2 and obtained products 1-5-RT$_8$@PS1-2 (copies of ^{1}H, ^{13}C, and ^{29}Si NMR spectra)……………………………………………… ••• S 5--

3. Stacked FT-IR spectra of starting material (PS2), substrates and the resulting products ••• S 49--

4. References………………………………………………………………………….. ••• S 49--

1. Table S1 of compounds

Structure	Compound Abbrev.	NMR spectra page:
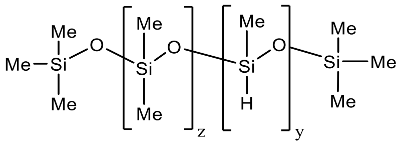	**PS1**	S 5
	**PS2**	S 7
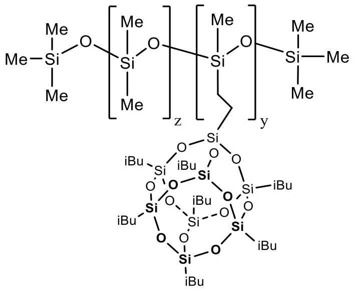	**1-iBuT$_8$@PS1**	S 9
	**1-iBuT$_8$@PS2**	S 11
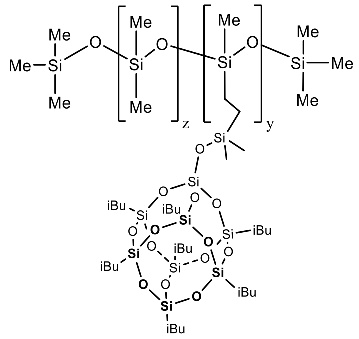	**2-iBuT$_8$@PS1**	S 13
	**2-iBuT$_8$@PS2**	S15
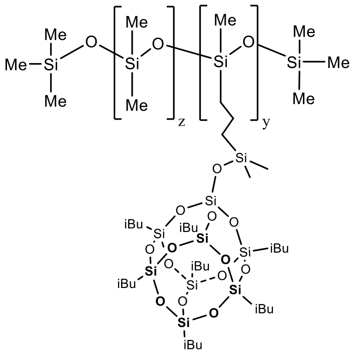	**3-iBuT$_8$@PS1**	S 17
	**3-iBuT$_8$@PS2**	S 19
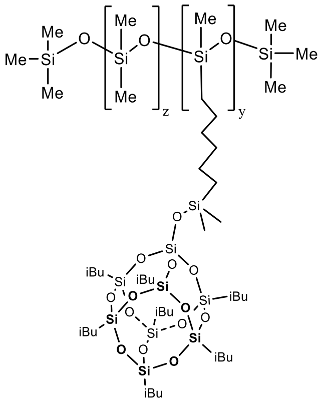	**4-iBuT$_8$@PS1**	S 21
	**4-iBuT$_8$@PS2**	S 23

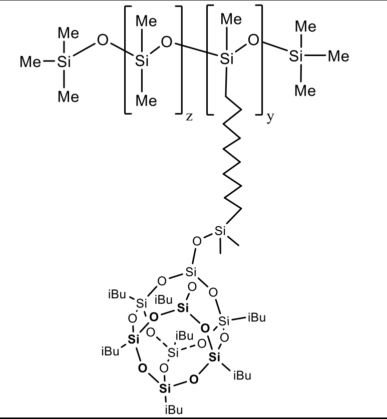	**5-iBuT$_8$@PS1**	S 25
	**5-iBuT$_8$@PS2**	S 27
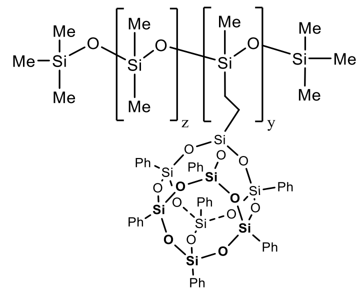	**1-PhT$_8$@PS1**	S 29
	**1-PhT$_8$@PS2**	S 31
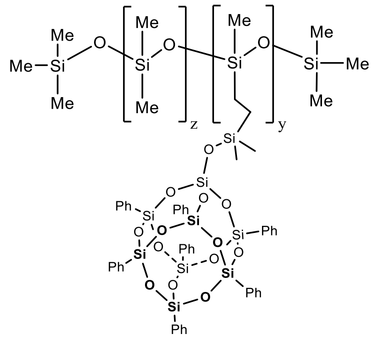	**2-PhT$_8$@PS1**	S 33
	**2-PhT$_8$@PS2**	S 35
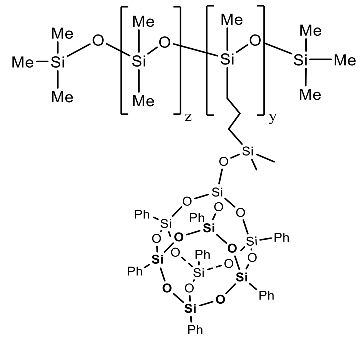	**3-PhT$_8$@PS1**	S 37
	**3-PhT$_8$@PS2**	S 39
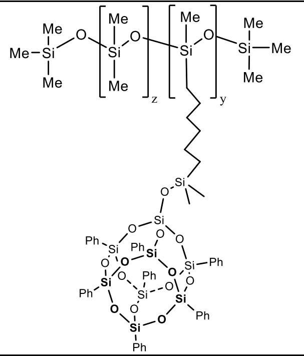	**4-PhT$_8$@PS1**	S 41
	**4-PhT$_8$@PS2**	S 43

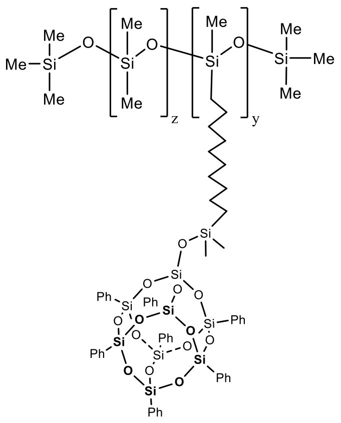	**5-PhT$_8$@PS1**	S 45
	**5-PhT$_8$@PS2**	S 47

2. **Data characterizing PS1-2 and obtained products 1-5-RT$_8$@PS1-2 (copies of ^{1}H, ^{13}C, and ^{29}Si NMR spectra)**

PS1 (4.3 mmol/g Si-H)

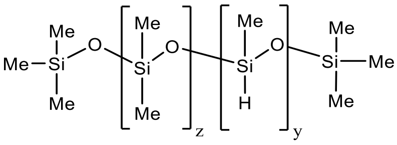

^{1}H NMR (300.2 MHz, $CDCl_3$, δ, ppm): 0.05-0.18 (m, -SiC***H$_3$***), 4.69-4.71 (s, -Si-***H***). **^{13}C NMR** (100.6 MHz, $CDCl_3$, δ, ppm): 1.04-1.34, 1.53, 1.86, 1.99 (-Si***C***H$_3$). **^{29}Si NMR** (79.5 MHz, $CDCl_3$, δ, ppm): 9.50-9.39, 7.57-7.15, -18.92 ,-19.21, -20.36, -20.64, -21.66, -22.02 (-***Si***-CH_3), -35.11, -35.38, -36.03, -36.51, -37.11, -37.34, -37.58 (-***Si***-H).
FT-IR (cm^{-1}): 2962.21, 2904.18 (-CH_3), 2156.57 (Si-H), 1411.40 (-C-H), 1257.63 (Si-C), 1015.81 (Si-O), 908.42 (Si-H).

The assignments are consistent with those in the literature.[1–3]

^{1}H NMR

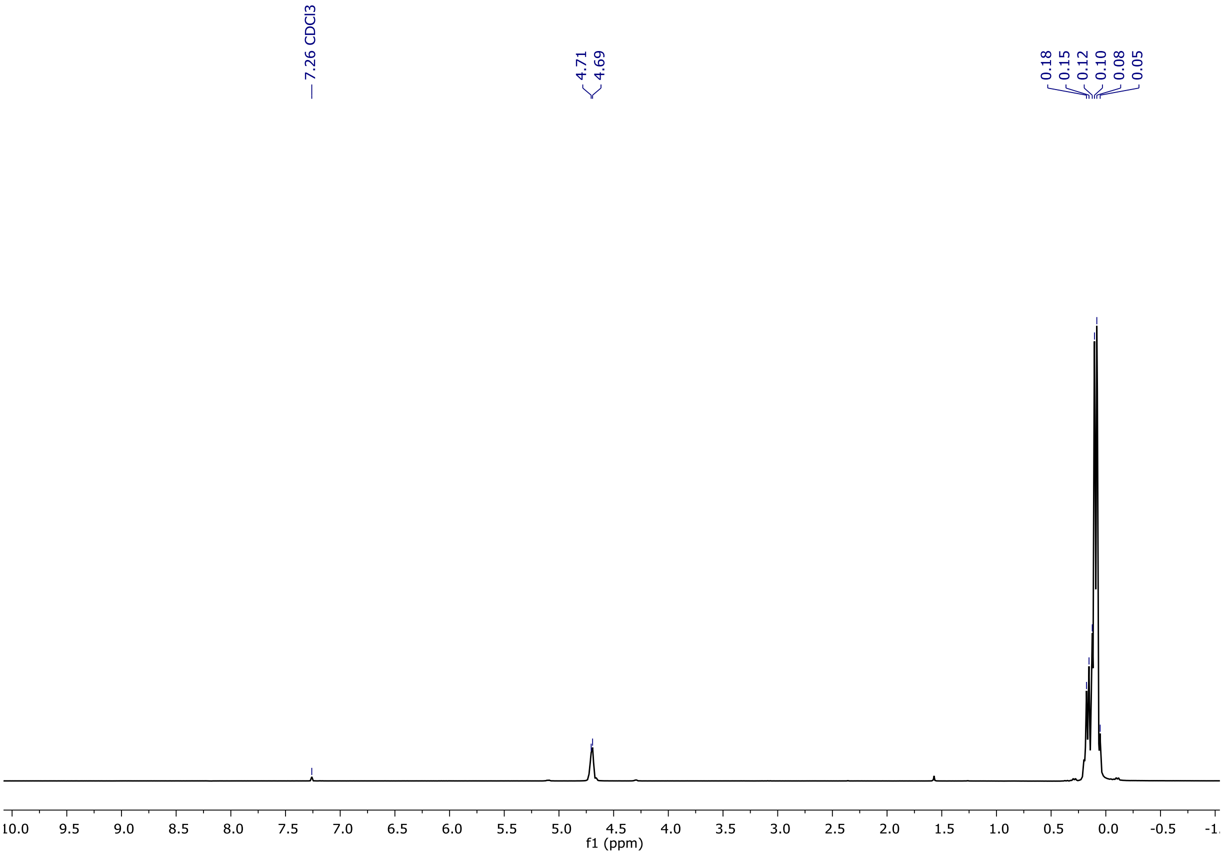

^{13}C NMR

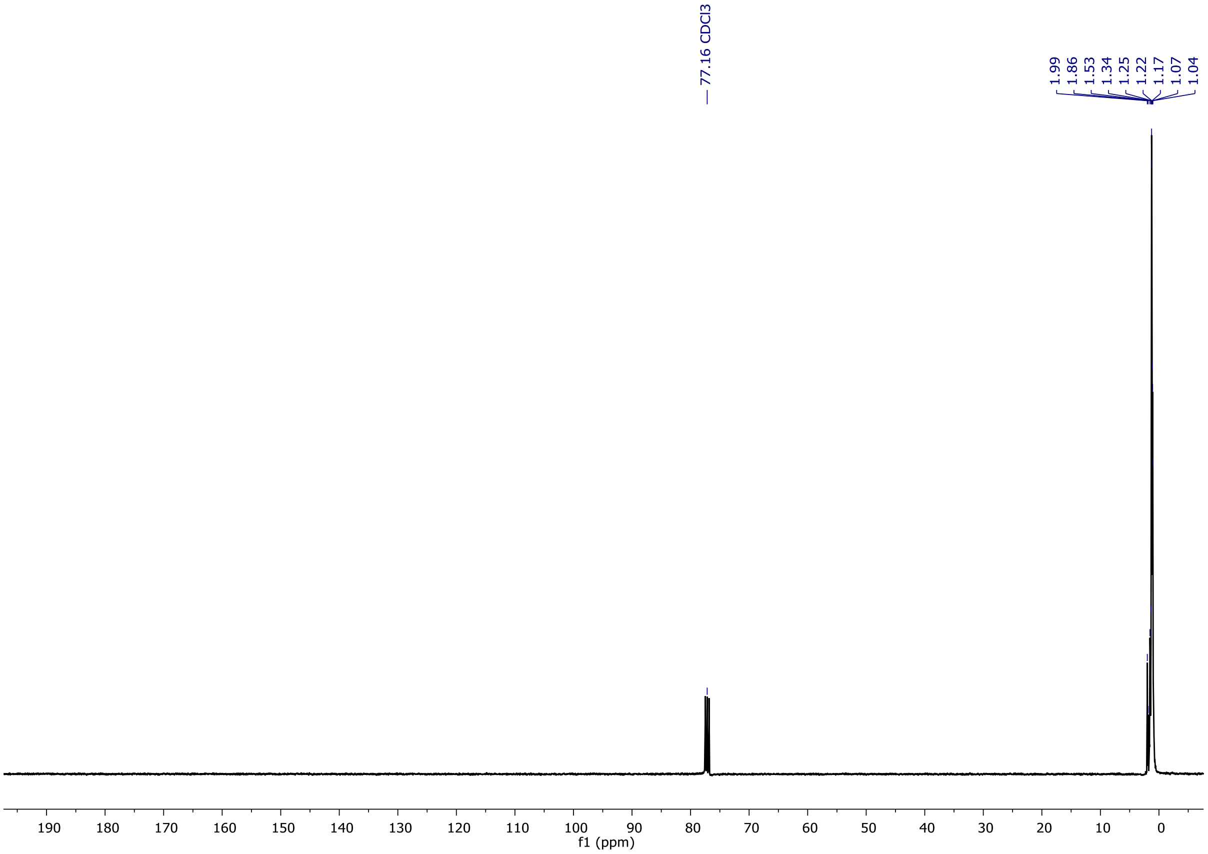

^{29}Si NMR

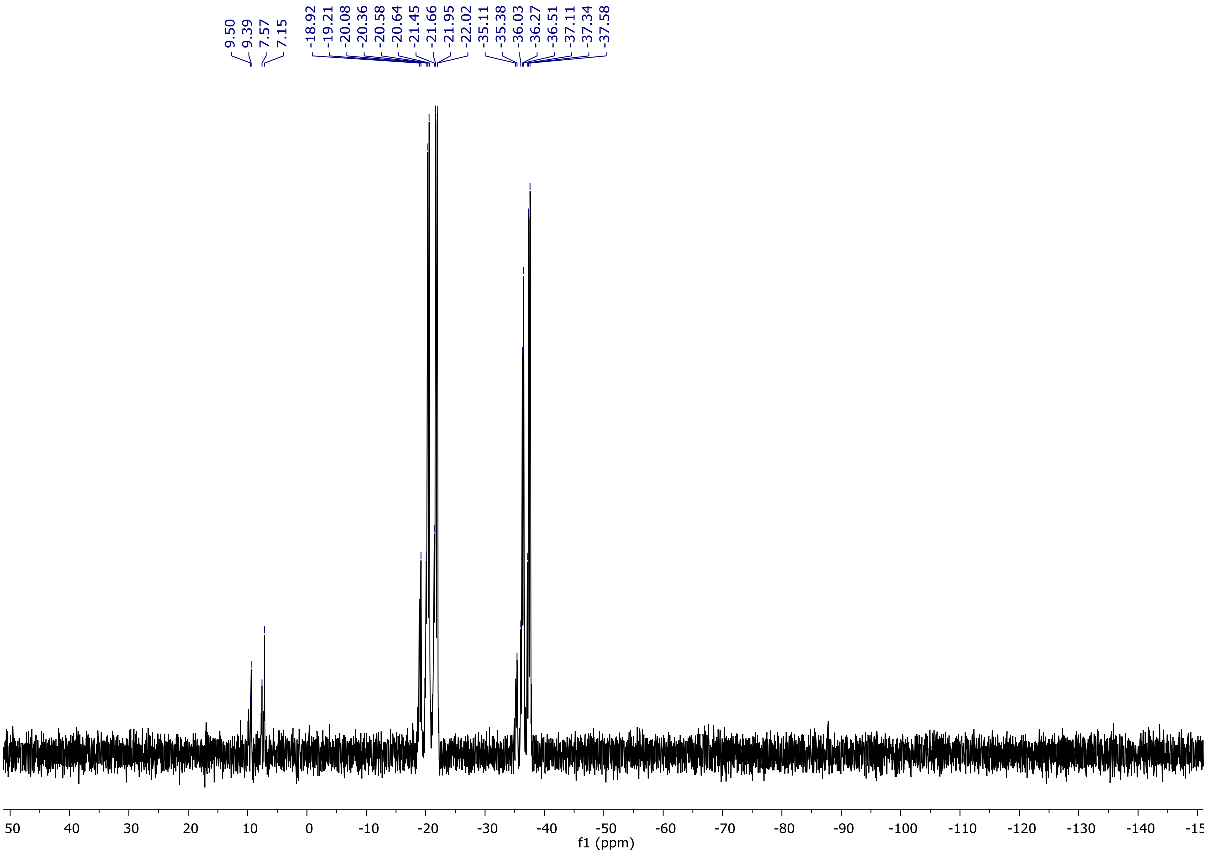

PS2 (1.1 mmol/g Si-H)

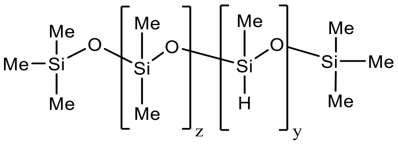

^{1}H NMR (300.2 MHz, $CDCl_3$, δ, ppm): 0.05-0.15 (m, -SiC***H_3***), 4.68 (s, -Si-***H***). **^{13}C NMR** (100.6 MHz, $CDCl_3$, δ, ppm): 0.97, 1.03, 1.14-1.21, 1.49, 1.96 (-Si***C***H_3). **^{29}Si NMR** (79.5 MHz, $CDCl_3$, δ, ppm): 7.24, -20.27, -20.56, -21.65, -21.86, -21.94 (-***Si***-CH_3), -37.58 (-***Si***-H).
FT-IR (cm^{-1}): 2962.21, 2904.18 (-CH_3), 2152.80 (Si-H), 1411.76 (-C-H), 1257.21 (Si-CH_3), 1078.08, 1009.60 (Si-O), 910.77 (Si-H).

The assignments are consistent with those in the literature.[1–3]

^{1}H NMR

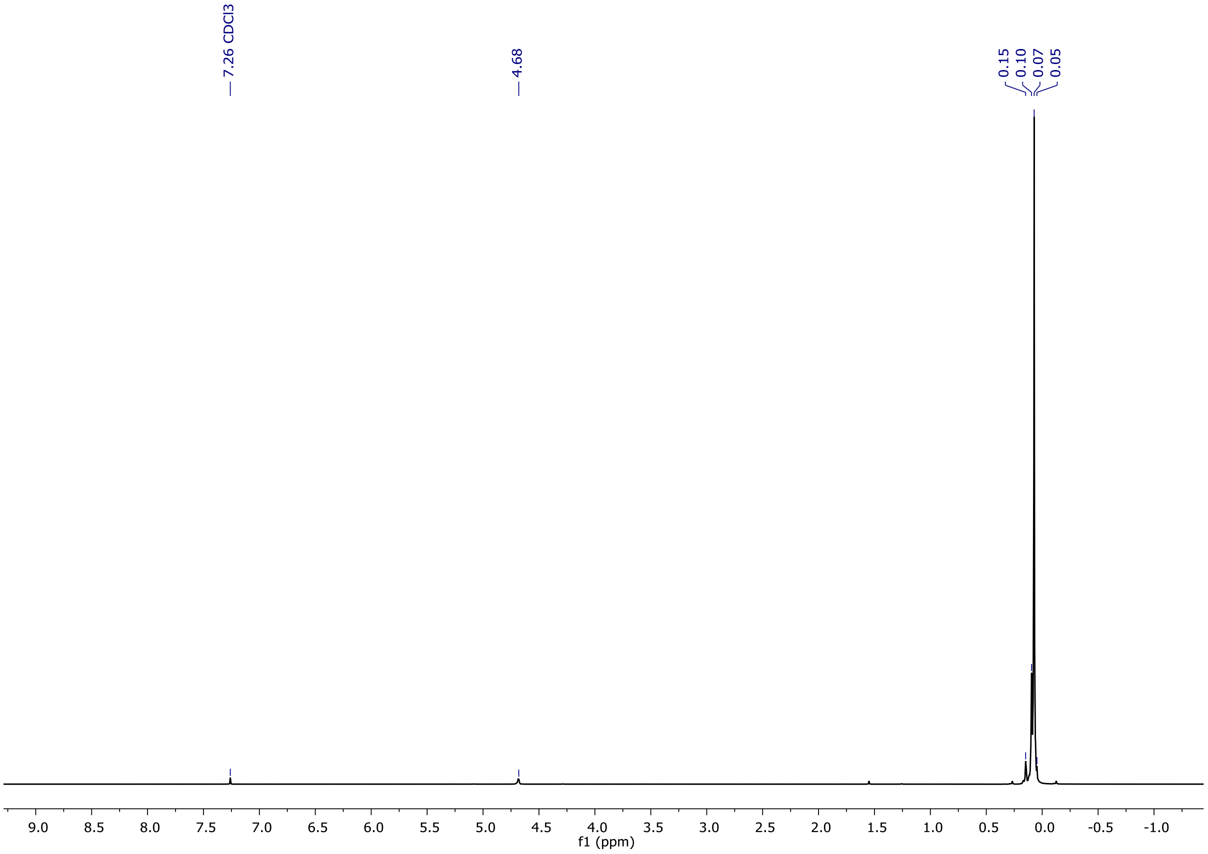

^{13}C NMR

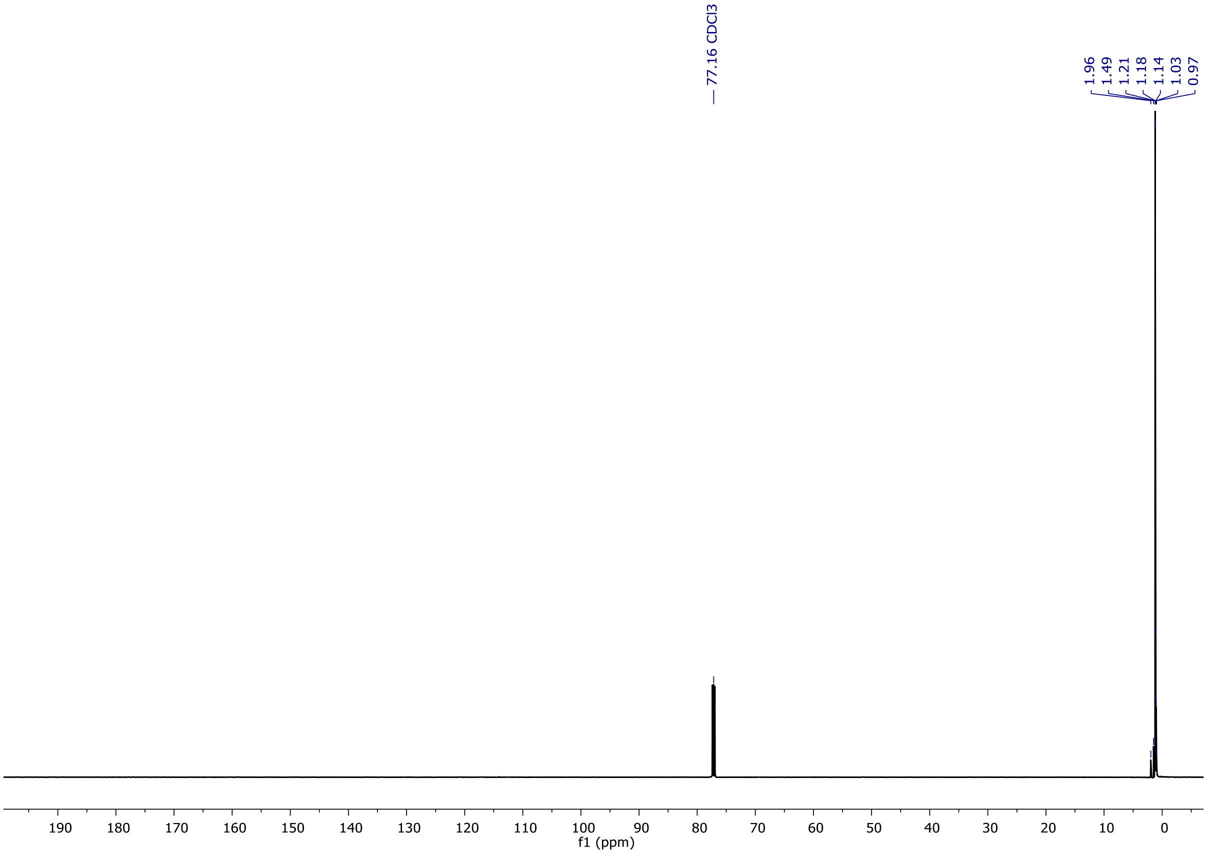

^{29}Si NMR

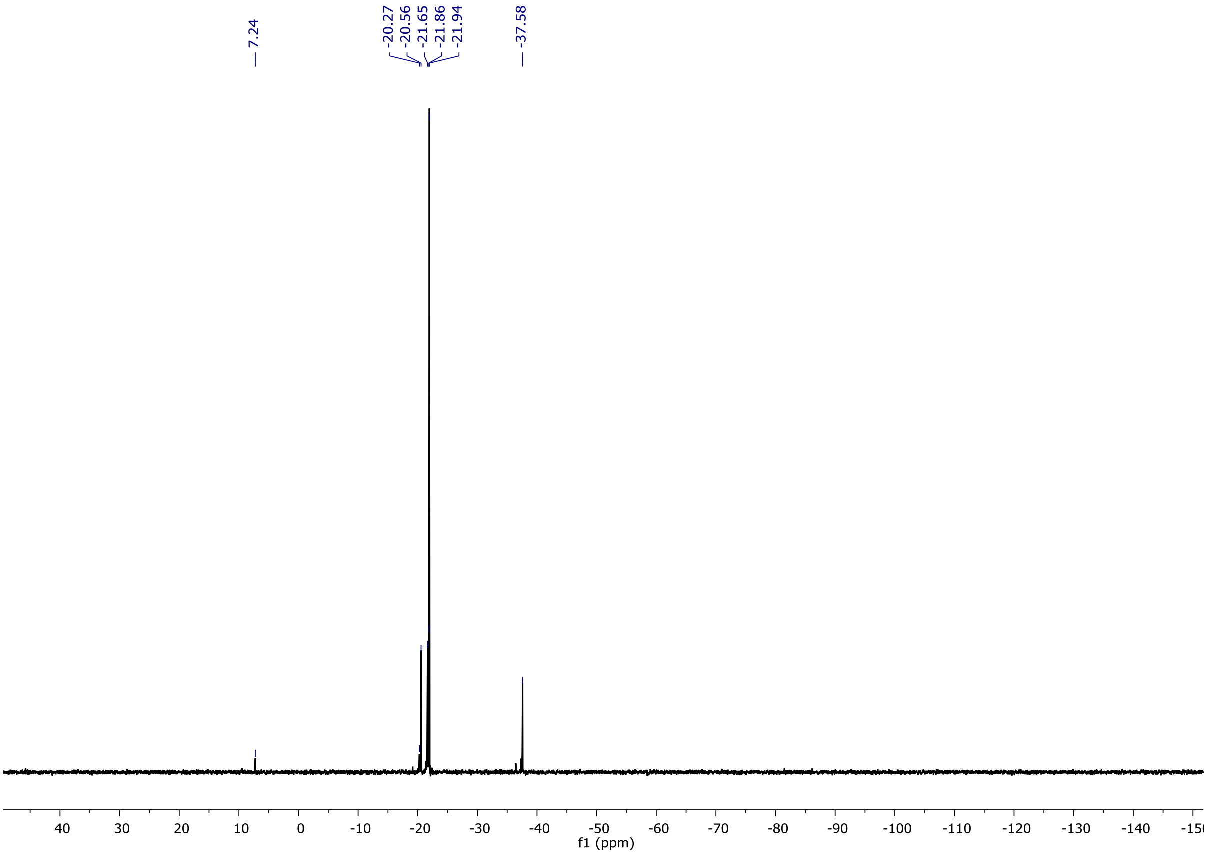

1-iBuT$_8$@PS

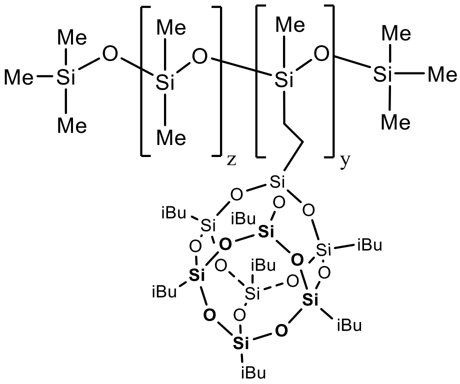

1-iBuT$_8$@PS1

^{1}H NMR (300.2 MHz, $CDCl_3$, δ, ppm): 0.04-0.09 (m, -SiC***H₃***), 0.51, 0.58-0.60 (m, -C***H₂***-, -C***H₂***- (iBu)), 0.94-0.96 (m, -C***H₃*** (iBu)), 1.81-1.90 (m, -C***H***- (iBu)). **^{13}C NMR** (100.6 MHz, $CDCl_3$, δ, ppm): 1.18 (-Si***C***H₃), 3.81, 8.52 (-***C***H₂-), 22.63-22.68, 24.02, 25.85 (iBu). **^{29}Si NMR** (79.5 MHz, $CDCl_3$, δ, ppm): -21.69, -21.94, -22.06, -22.28 (-***Si***CH₃), -66.87 (-***Si***-CH₂-CH₂-Si-), -67.42, -67.65, -67.94.
FT-IR (cm^{-1}): 2953.20, 2905,52, 2869.27 (-C-H), 1464.62 (-C-H), 1259.49, 1228.75 (Si-C), 1084.86 (Si-O).

^{1}H NMR

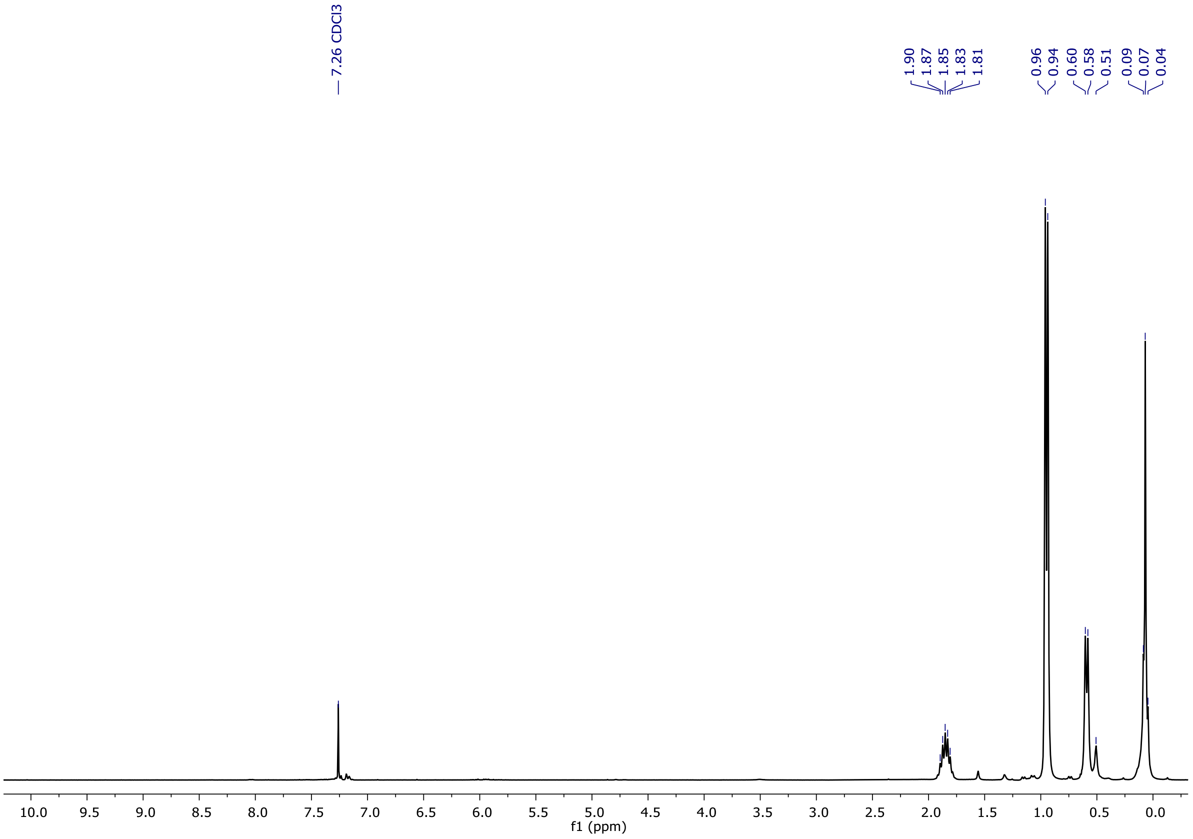

^{13}C NMR

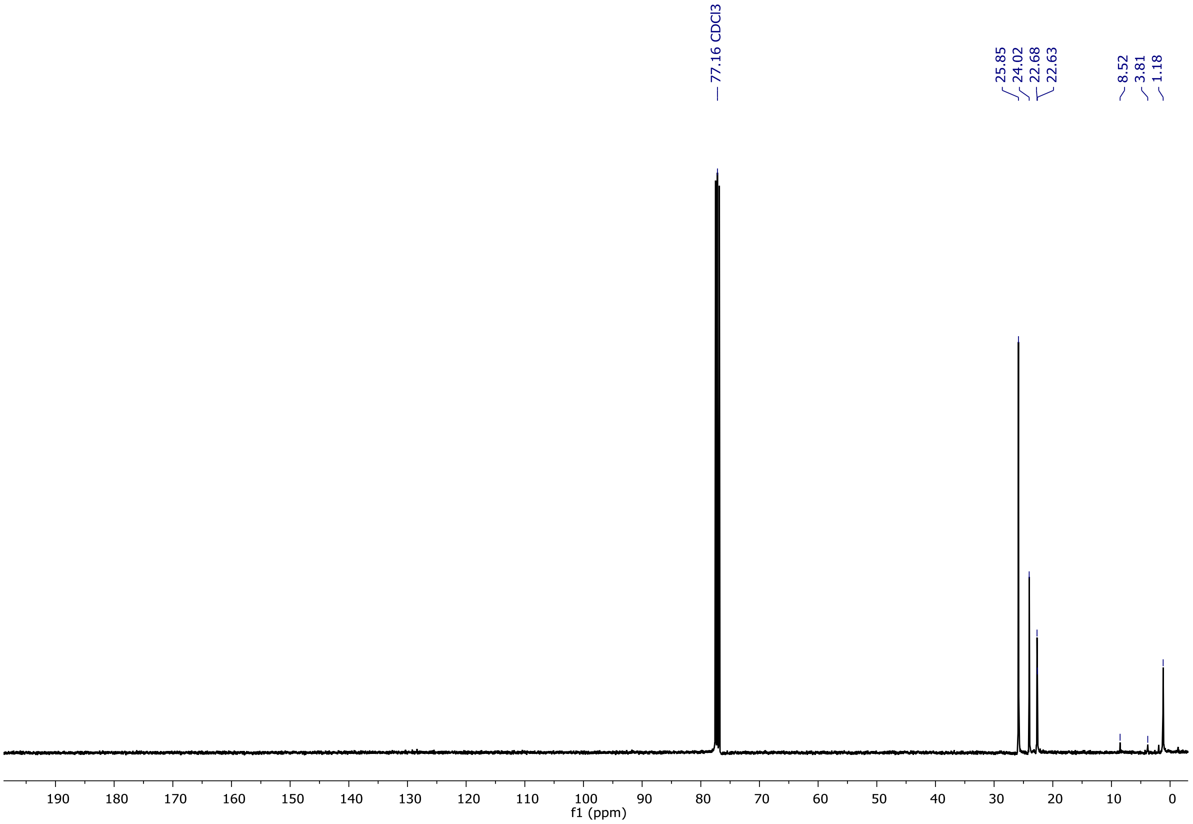

^{29}Si NMR

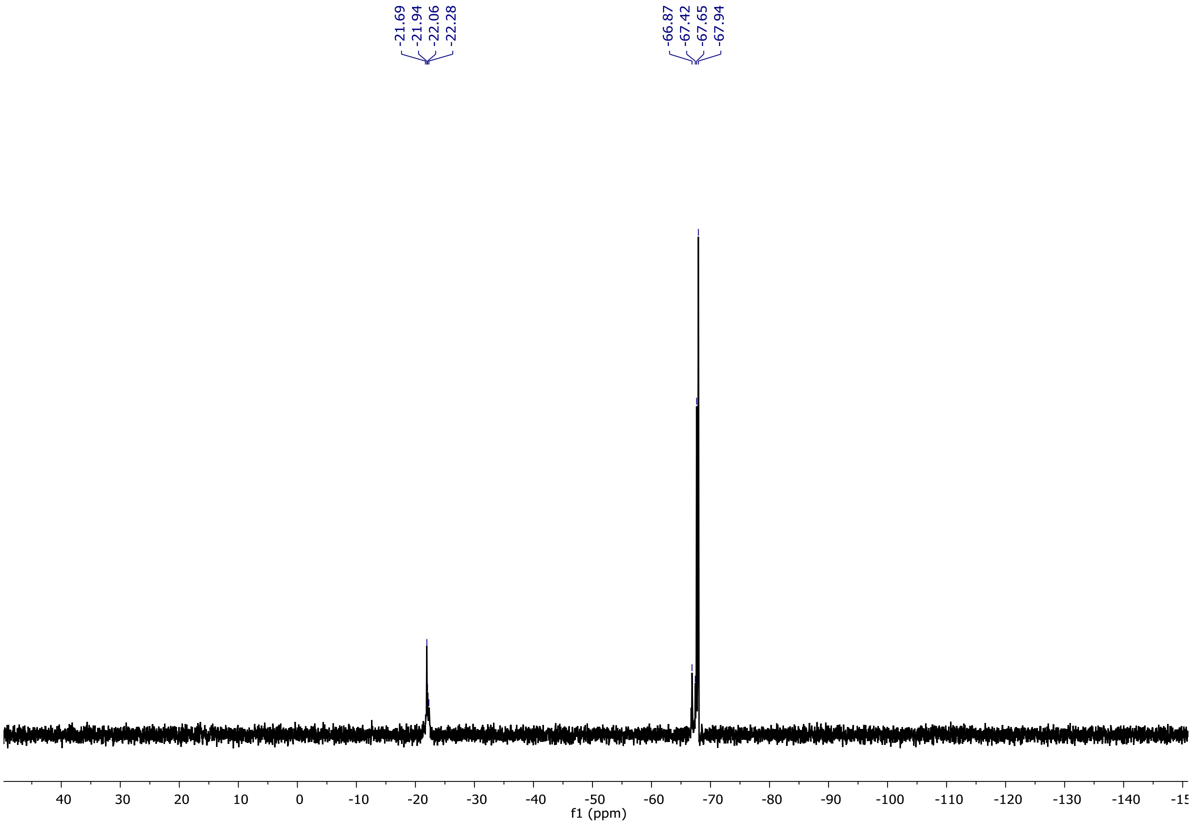

1-iBuT$_8$@PS2

^{1}H NMR (300.2 MHz, $CDCl_3$, δ, ppm): 0.05-0.14 (m, -SiC***H*$_3$**), 0.51, 0.58-0.64 (m, -C***H*$_2$**-, -C***H*$_2$**- (iBu)), 0.94-0.96 (m, -C***H*$_3$** (iBu)), 1.79-1.92 (m, -C***H***- (iBu)). **^{13}C NMR** (100.6 MHz, $CDCl_3$, δ, ppm): 1.21 (-Si***C***H$_3$), 3.85, 8.57 (-***C***H$_2$-), 22.62, 24.05, 25.87 (iBu). **^{29}Si NMR** (79.5 MHz, $CDCl_3$, δ, ppm): -21.94, -22.33 (-***Si***CH$_3$), -66.84 (-***Si***-CH$_2$-CH$_2$-Si-), -67.41, -67.63, -67.93.
FT-IR (cm^{-1}): 2957.69, 2905.43, 2870.93 (-C-H), 1465.31 (-C-H), 1258.18, 1228.59 (Si-C), 1084.43, 1009.79 (Si-O).

^{1}H NMR

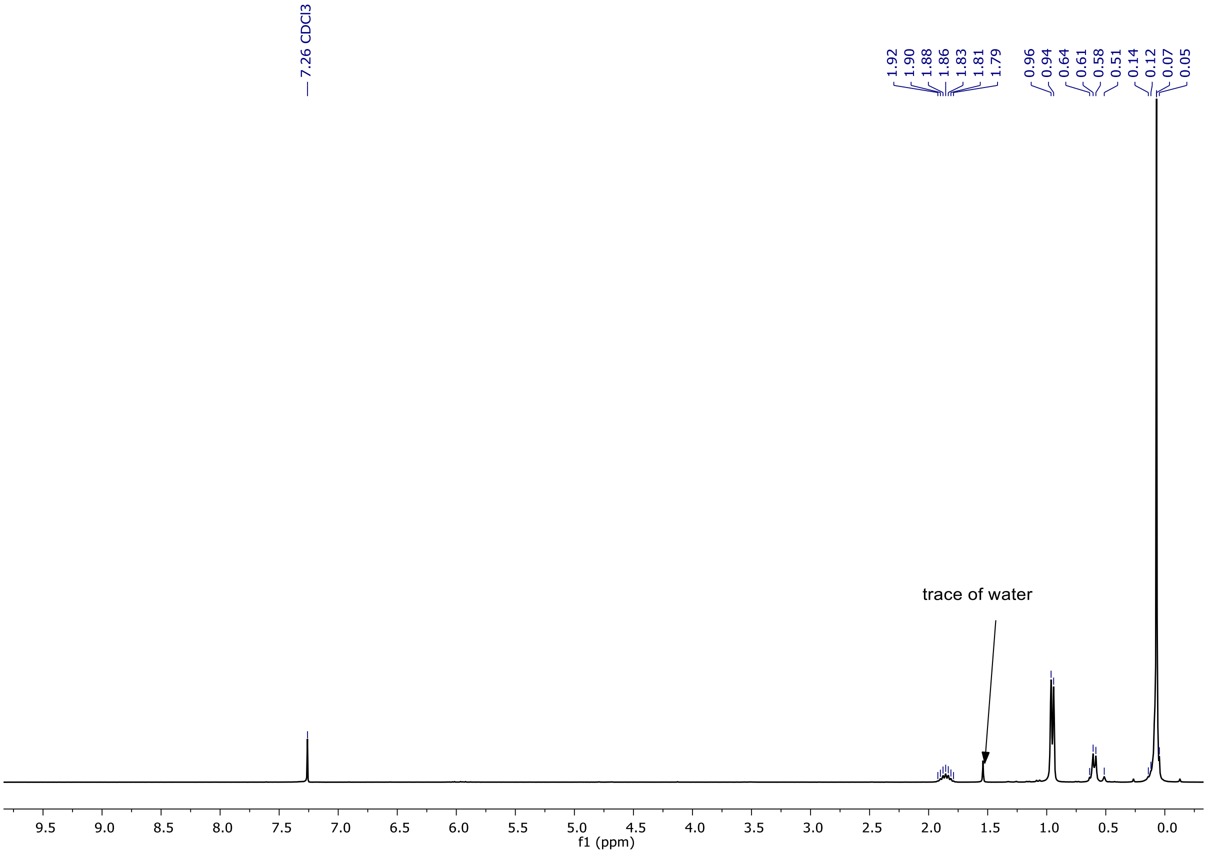

^{13}C NMR

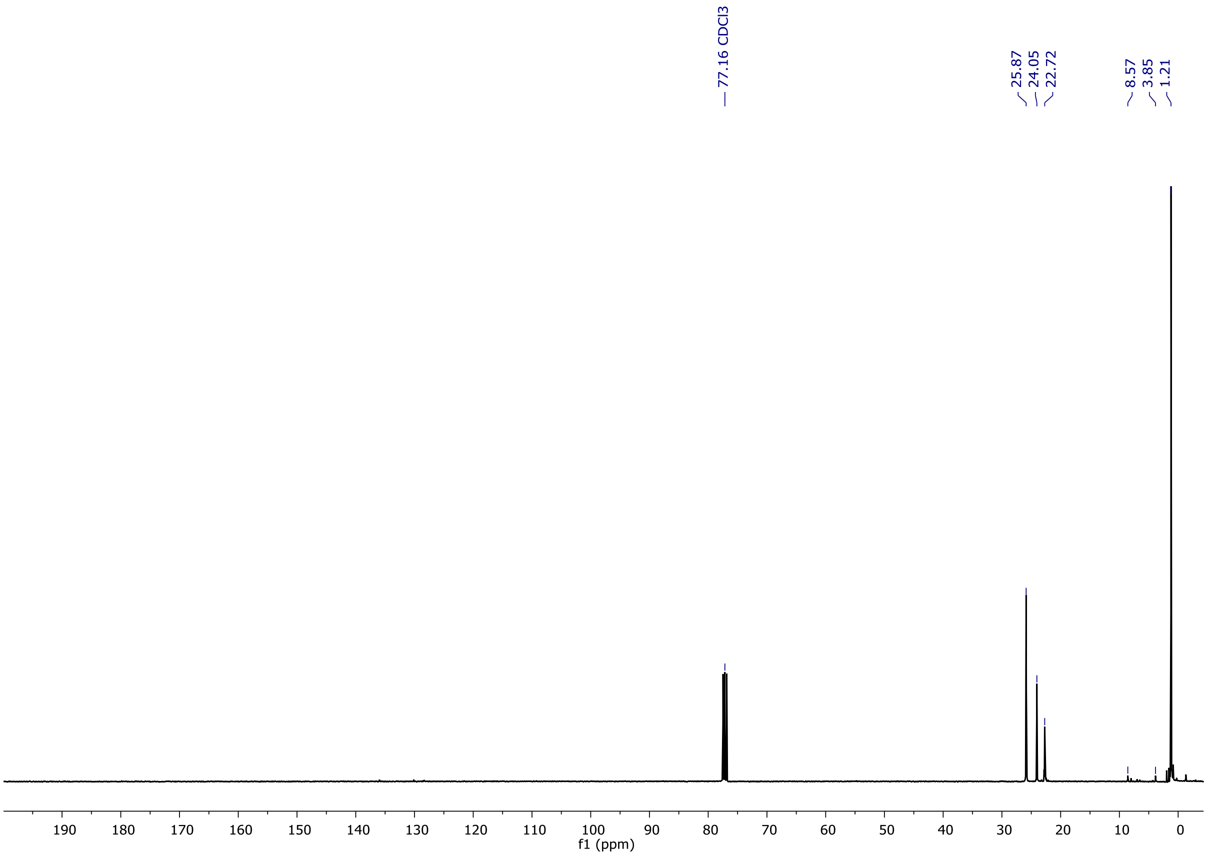

^{29}Si NMR

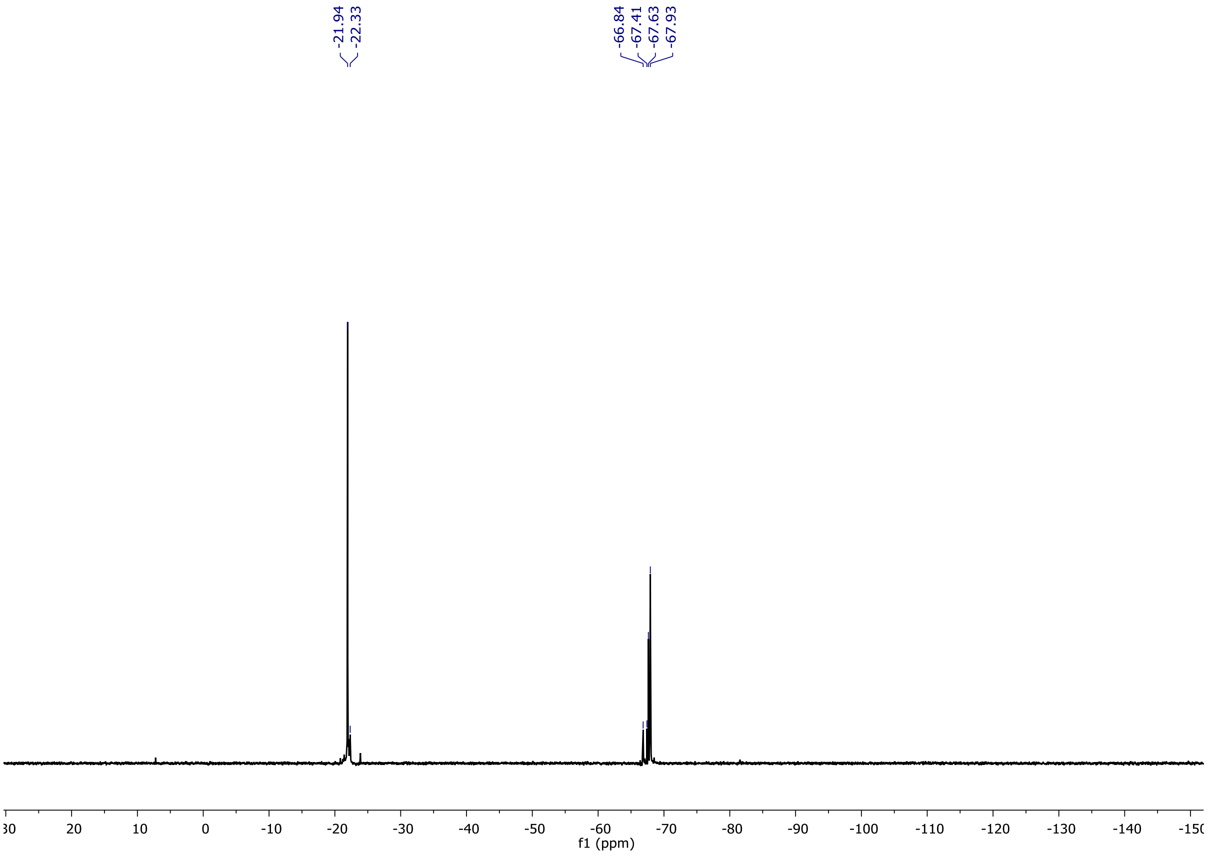

2-iBuT$_8$@PS

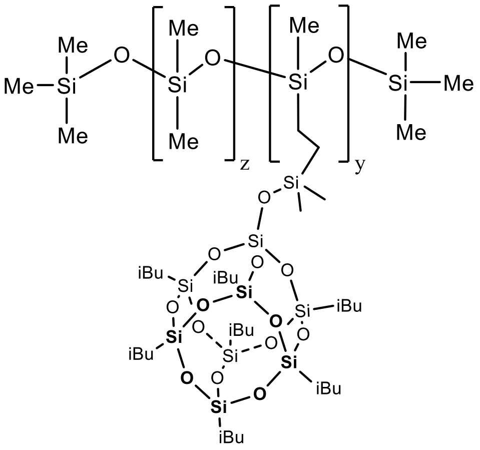

2-iBuT$_8$@PS1

^{1}H NMR (300.2 MHz, $CDCl_3$, δ, ppm): 0.07-0.09 (m, -SiC***H$_3$***), 0.39-0.41, 0.58-0.61 (m, -C***H$_2$***-, -C***H$_2$***-(iBu)), 0.94-0.96 (m, -C***H*$_3$** (iBu)), 1.83-1.88 (m, -C***H***- (iBu)). **^{13}C NMR** (100.6 MHz, $CDCl_3$, δ, ppm): -0.94, -0.87, 1.18 (-Si***C***H$_3$), 8.56, 8.95 (-***C***H$_2$-), 22.55-22.66, 23.97-24.01, 25.84 (iBu). **^{29}Si NMR** (79.5 MHz, $CDCl_3$, δ, ppm): 11.91(-***Si***-CH$_2$-CH$_2$-Si-), -22.03, -22.21, -22.60, (-***Si***CH$_3$), -67.11, -67.90, -109.66 (-***Si***O$_4$).
FT-IR (cm^{-1}): 2953.38, 2905.92, 2869.69 (-C-H), 1464.83 (-C-H), 1258.87, 1228.58 (Si-C), 1076.60 (Si-O).

^{1}H NMR

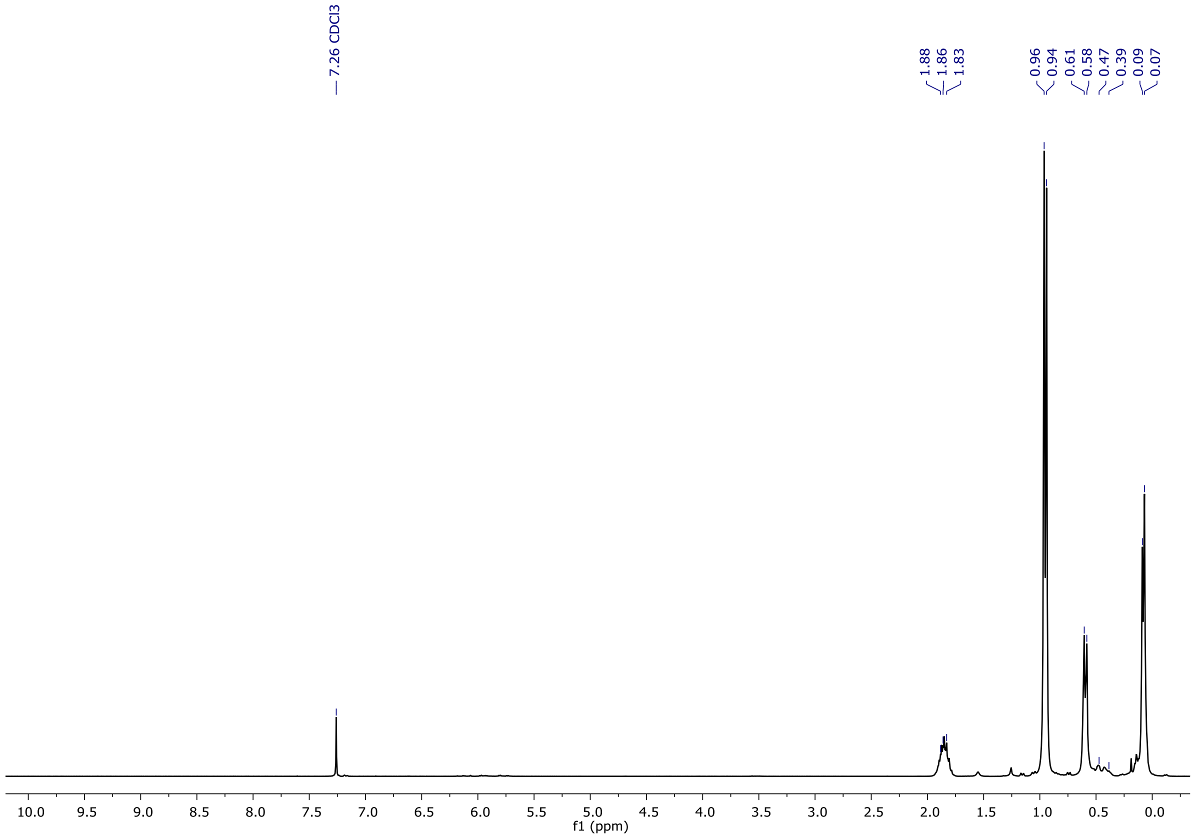

^{13}C NMR

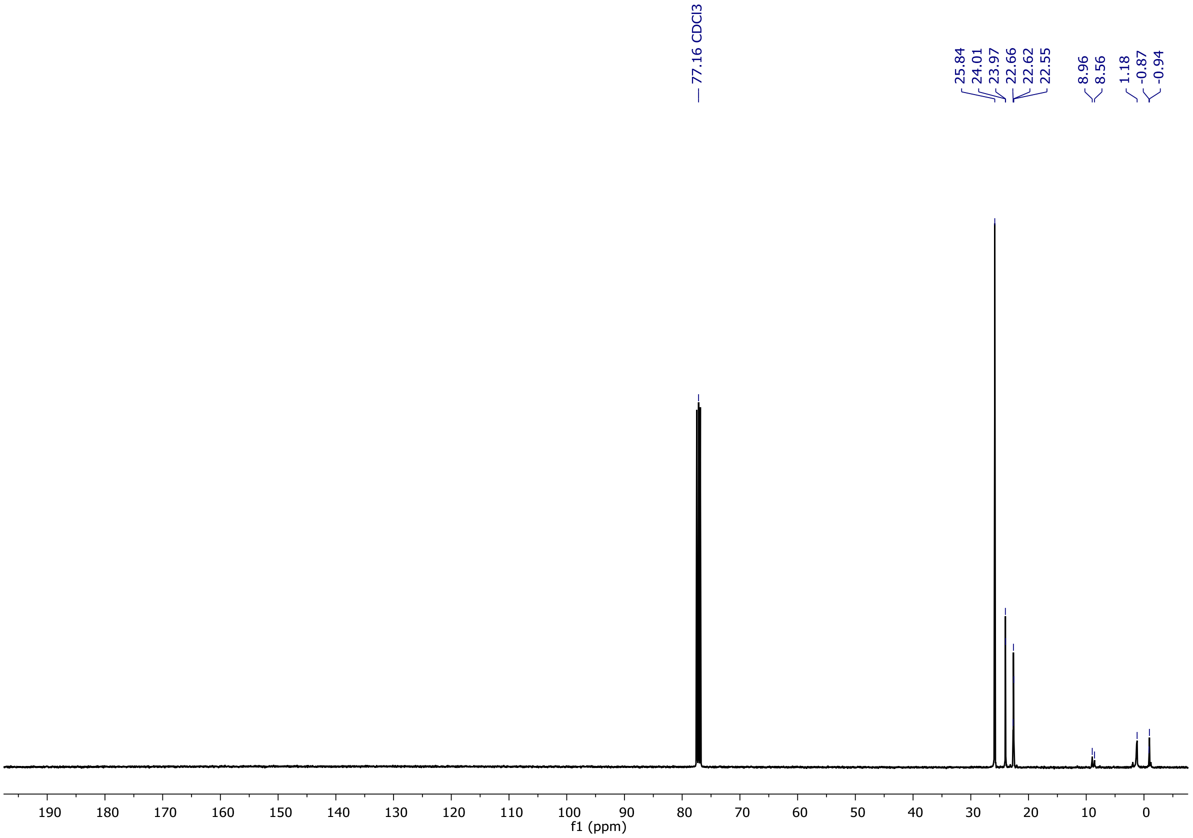

^{29}Si NMR

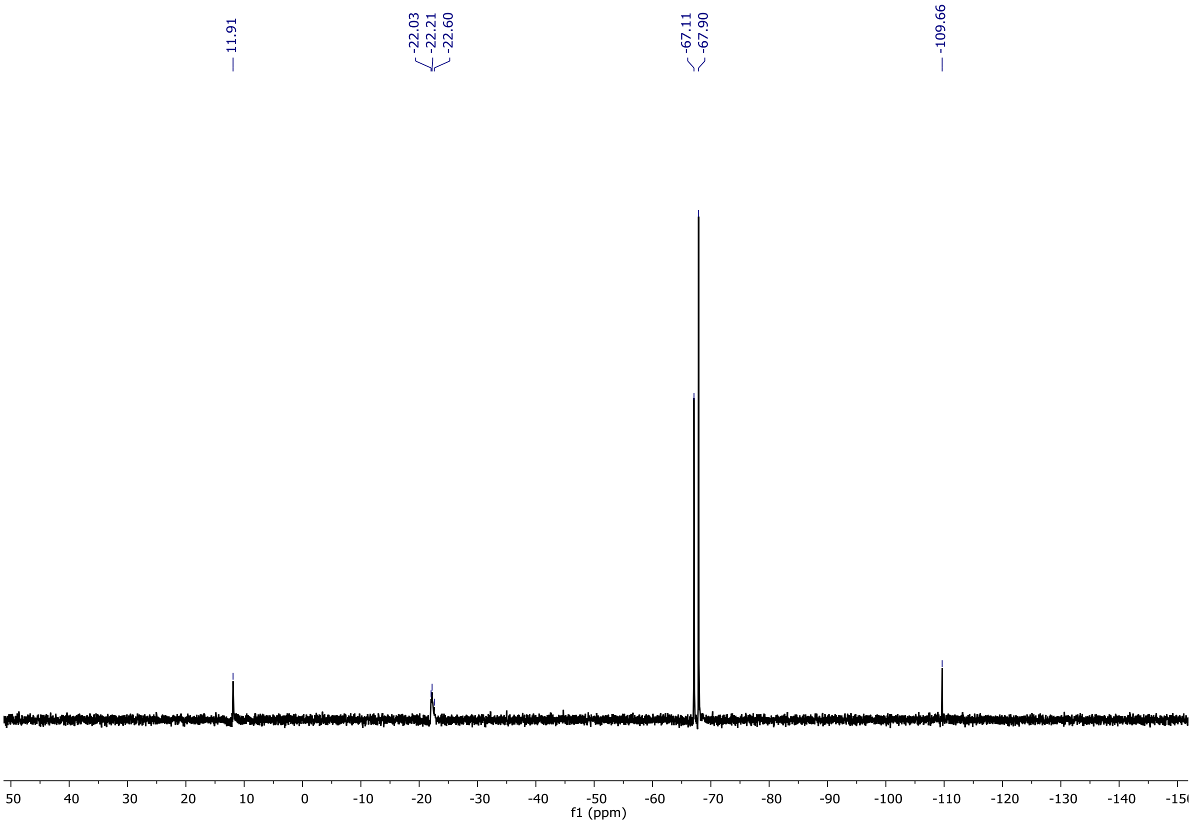

2-iBuT$_8$@PS2

^{1}H NMR (300.2 MHz, $CDCl_3$, δ, ppm): 0.07-0.13 (m, -SiC***H$_3$***), 0.49, 0.59-0.61 (m, -C***H$_2$***-, -C***H$_2$***- (iBu)), 0.95-0.96 (m, -C***H$_3$*** (iBu)), 1.82-1.90 (m, -C***H***- (iBu)). **^{13}C NMR** (100.6 MHz, $CDCl_3$, δ, ppm): -0.91, 1.21, 1.31 (-Si***C***H$_3$), 8.59, 9.01 (-***C***H$_2$-), 22.59-22.65, 24.04, 25.87 (iBu). **^{29}Si NMR** (79.5 MHz, $CDCl_3$, δ, ppm): 11.96 (-***Si***-CH$_2$-CH$_2$-Si-), -21.94, -22.16 (-***Si***CH$_3$), -67.10, -67.89, -109.66 (-***Si***O$_4$).
FT-IR (cm^{-1}): 2955.38, 2924.79, 2869.81 (-C-H), 1464.98 (-C-H), 1259.06, 1229.16 (Si-C), 1089.76, 1017.18 (Si-O).

^{1}H NMR

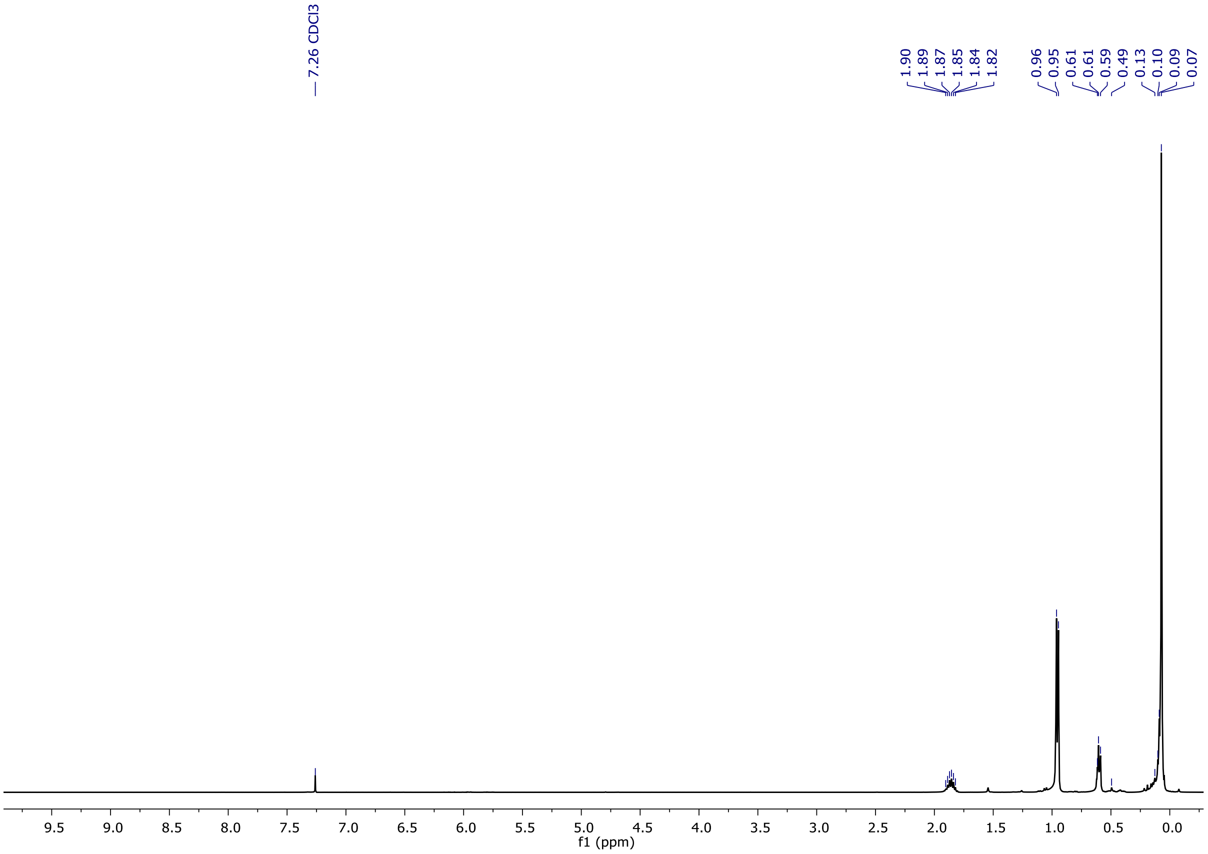

^{13}C NMR

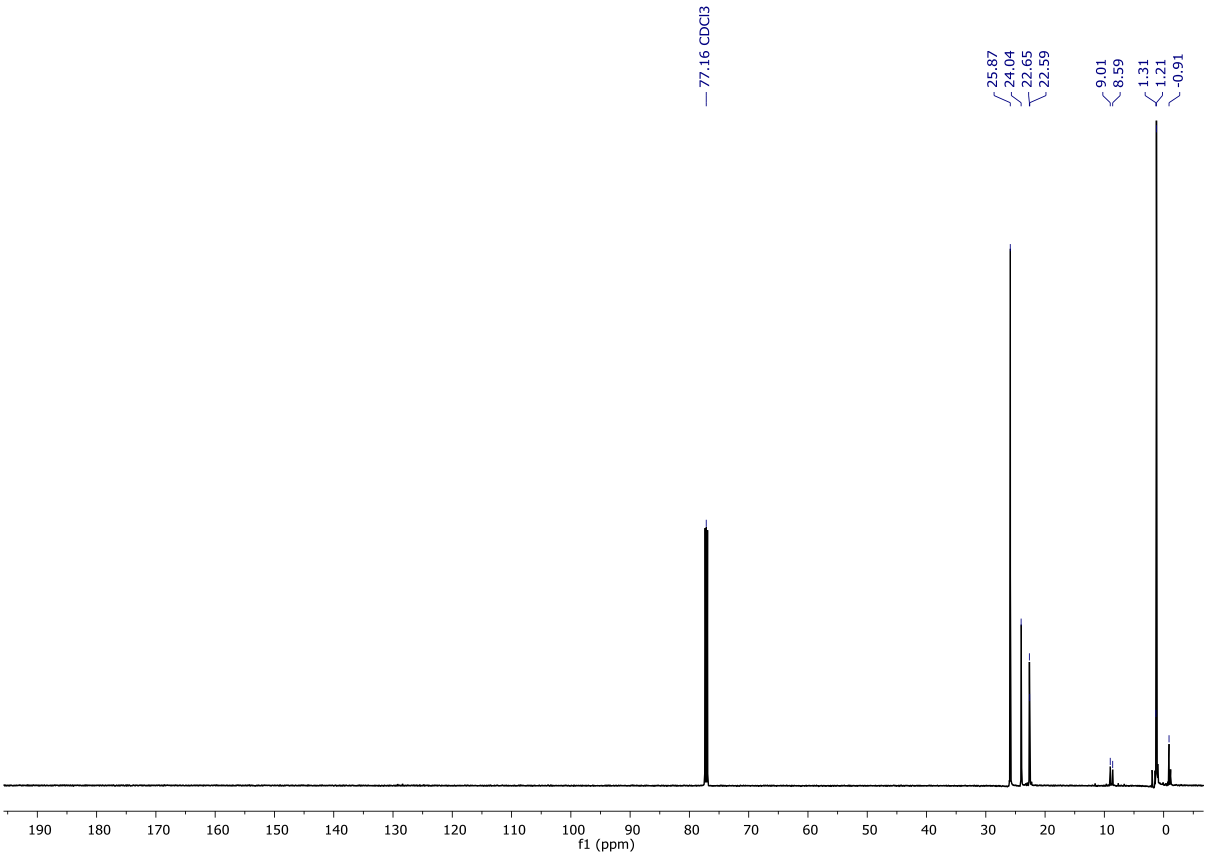

^{29}Si NMR

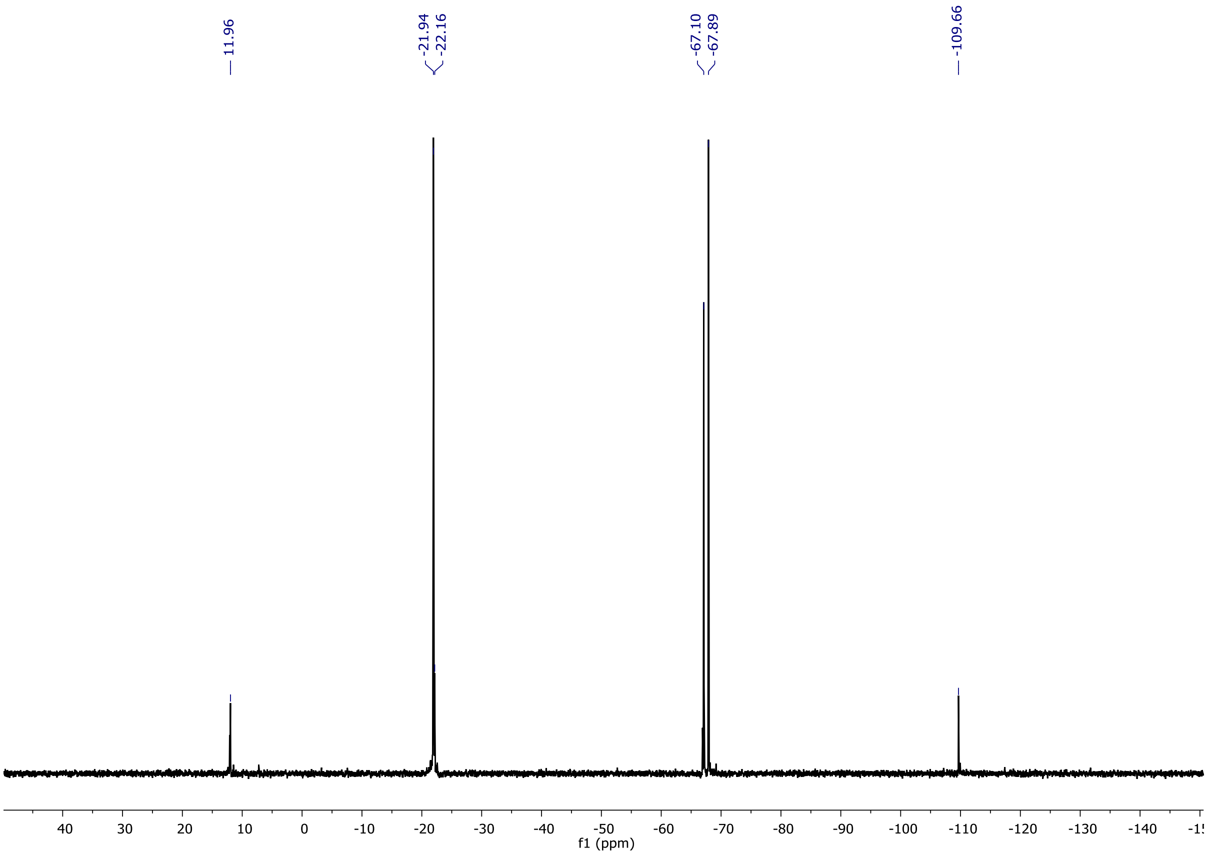

3-iBuT$_8$@PS

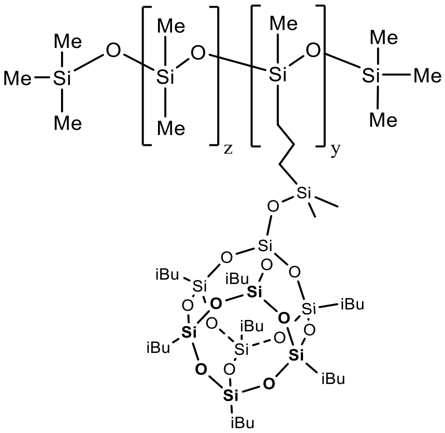

3-iBuT$_8$@PS1

^{1}H NMR (300.2 MHz, $CDCl_3$, δ, ppm): 0.07-0.09 (m, -SiC$\boldsymbol{H_3}$), 0.58-0.68 (m, -C$\boldsymbol{H_2}$-, -C$\boldsymbol{H_2}$- (iBu)), 0.94-0.96 (m, -C$\boldsymbol{H_3}$ (iBu)), 1.37-1.39 (m, -C$\boldsymbol{H_2}$-), 1.80-1.89 (m, -C***H***- (iBu)), 5.61-5.67 and 6.11-6.23 (m, -C***H***=C***H***- from dehydrogenative silylation by-product). **^{13}C NMR** (100.6 MHz, $CDCl_3$, δ, ppm): -0.11, 1.22 (-Si***C***H$_3$), 16.88, 22.02, 22.20 (-***C***H$_2$-), 22.59-22.69, 24.00-24.04, 25.88 (iBu), 130.30 and 143.60 (-***C***H=***C***H- from dehydrogenative silylation by-product). **^{29}Si NMR** (79.5 MHz, $CDCl_3$, δ, ppm): 10.87 (-***Si***-CH$_2$-CH$_2$-CH$_2$-Si-), -21.94, -22.21, -23.00, (-***Si***CH$_3$), -67.11, -67.90, -109.66 (-***Si***O$_4$).
FT-IR (cm^{-1}): 2953.32, 2924.65, 2869.56 (-C-H), 1464.78 (-C-H), 1259.04, 1228.62 (Si-C), 1077.49 (Si-O).

^{1}H NMR

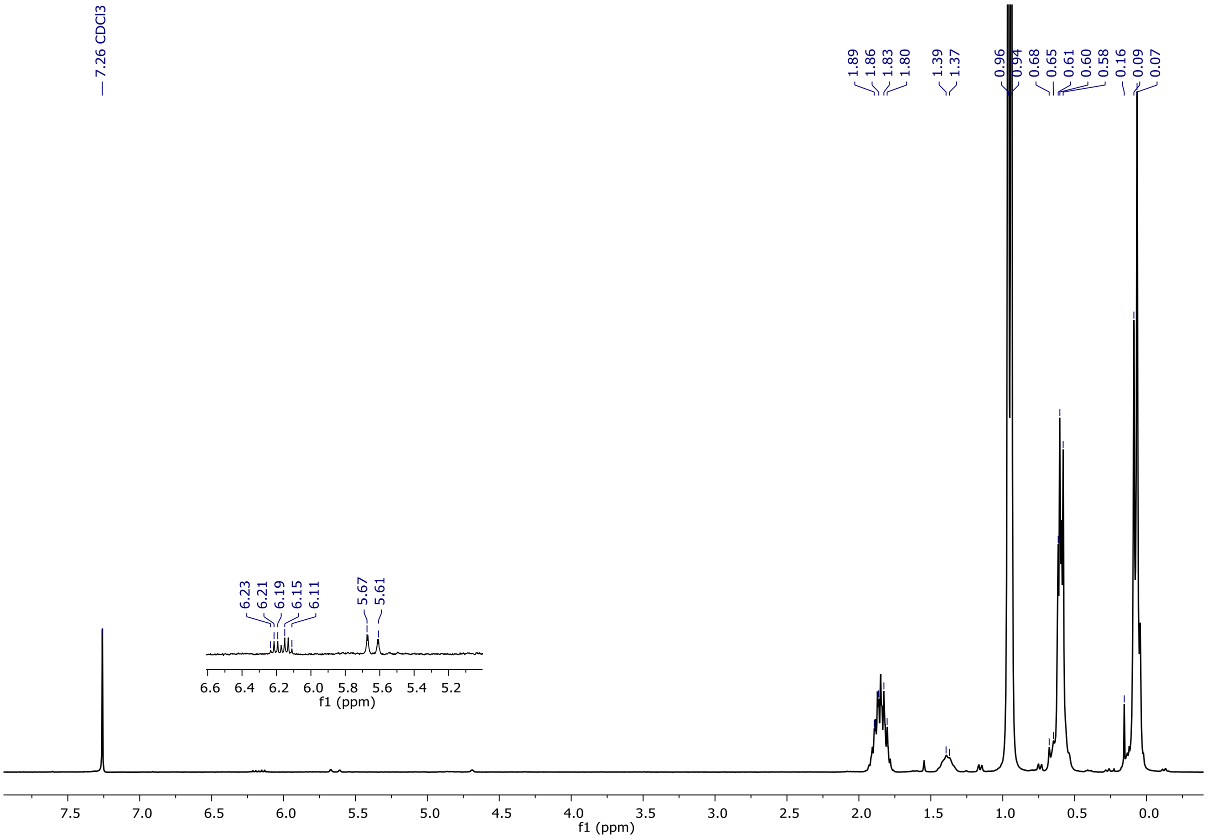

^{13}C NMR

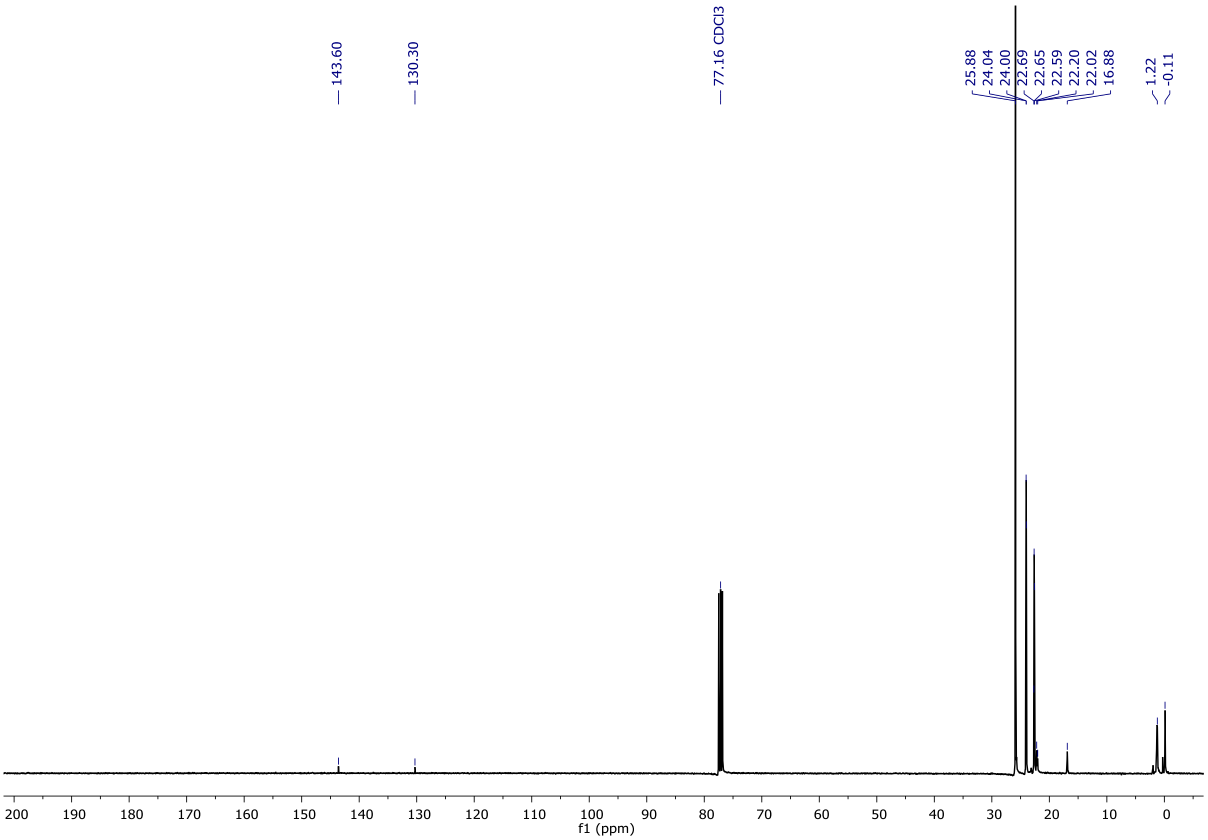

^{29}Si NMR

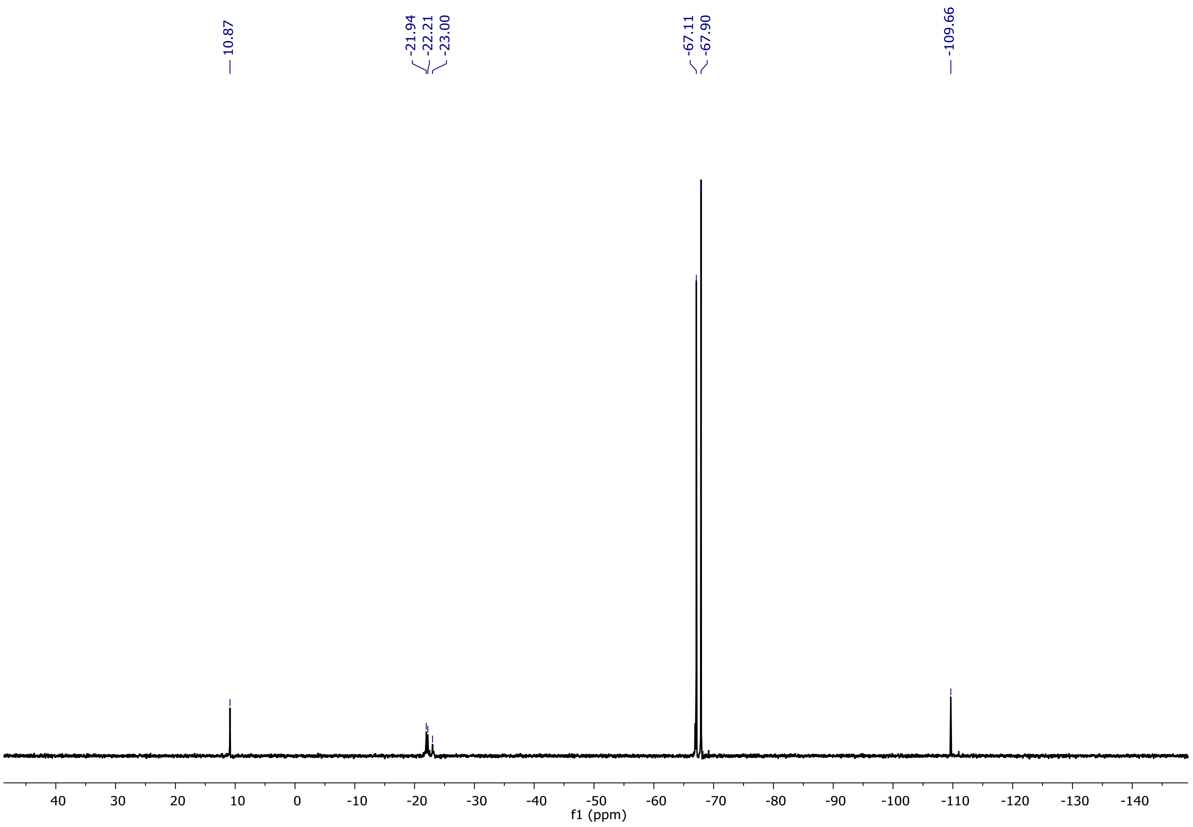

3-iBuT$_8$@PS2

^{1}H NMR (300.2 MHz, $CDCl_3$, δ, ppm): 0.07-0.09 (m, -SiC***H*₃**), 0.55-0.68 (m, -C***H*₂**-, -C***H*₂**- (iBu)), 0.94-0.96 (m, -C***H*₃** (iBu)), 1.38-1.43 (m, -C***H*₂**-), 1.81-1.91 (m, -C***H***- (iBu)), 5.61-5.68 and 6.13-6.24 (m, -C***H***=C***H***- from dehydrogenative silylation by-product). **^{13}C NMR** (100.6 MHz, $CDCl_3$, δ, ppm): -0.13, 1.20 (-Si***C***H_3), 16.87, 22.01, 22.19 (-***C***H_2-), 22.58-22.63, 23.99-24.03, 25.86 (iBu), 130.28 and 143.60 (-***C***H=***C***H- from dehydrogenative silylation by-product). **^{29}Si NMR** (79.5 MHz, $CDCl_3$, δ, ppm): 10.89 (-***Si***-CH_2-CH_2-CH_2-Si-), -21.94, -22.21, -22.98, (-***Si***CH_3), -67.10, -67.89, -109.65 (-***Si***O_4).
FT-IR (cm^{-1}): 2955.84, 2925.46, 2870.51 (-C-H), 1465.15 (-C-H), 1258.63, 1228.71 (Si-C), 1083.40, 1012.74 (Si-O).

^{1}H NMR

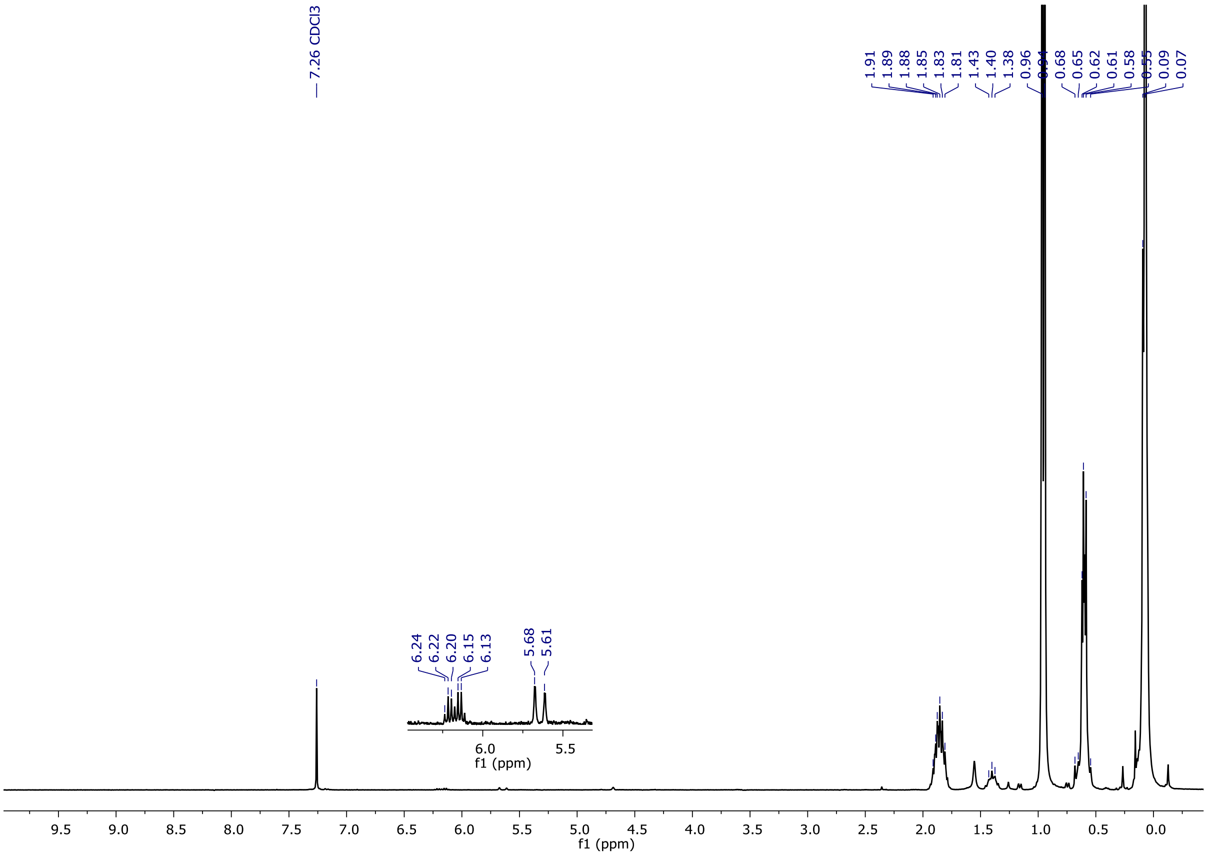

^{13}C NMR

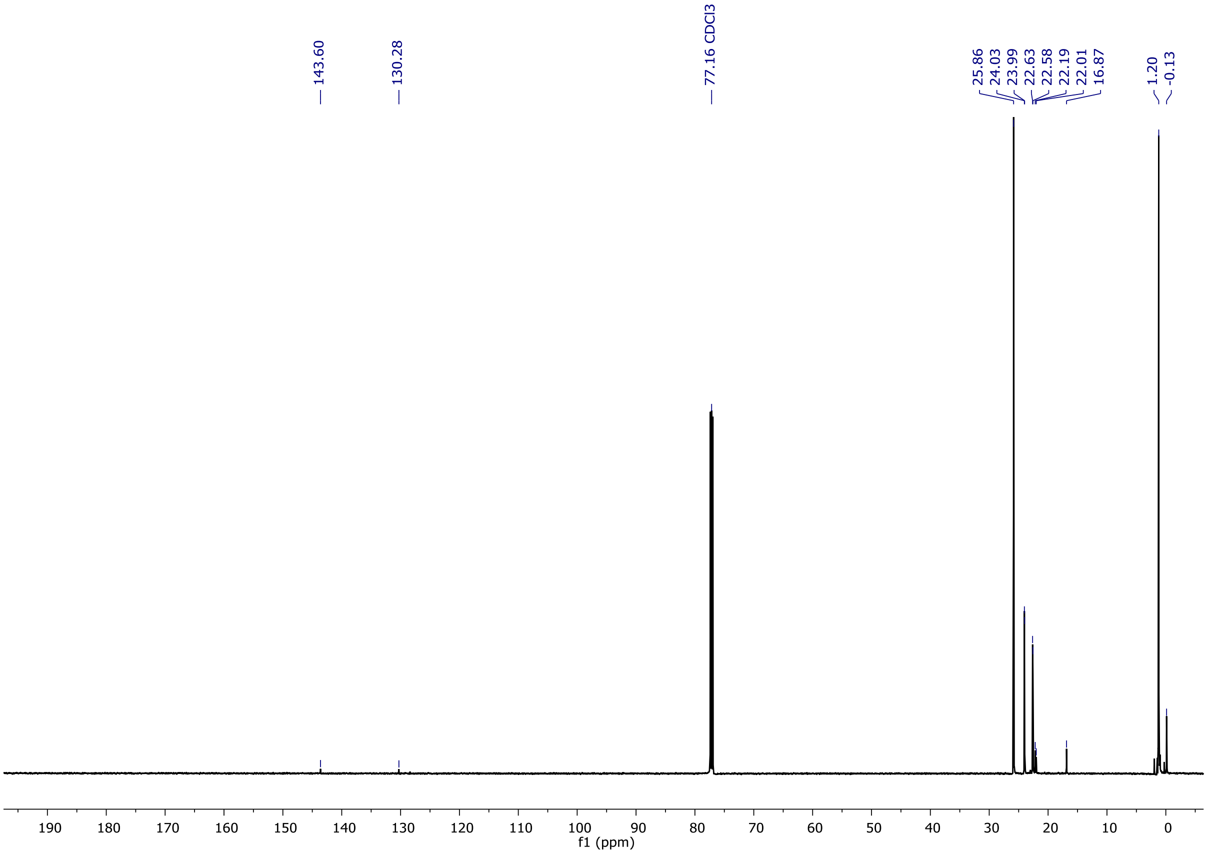

^{29}Si NMR

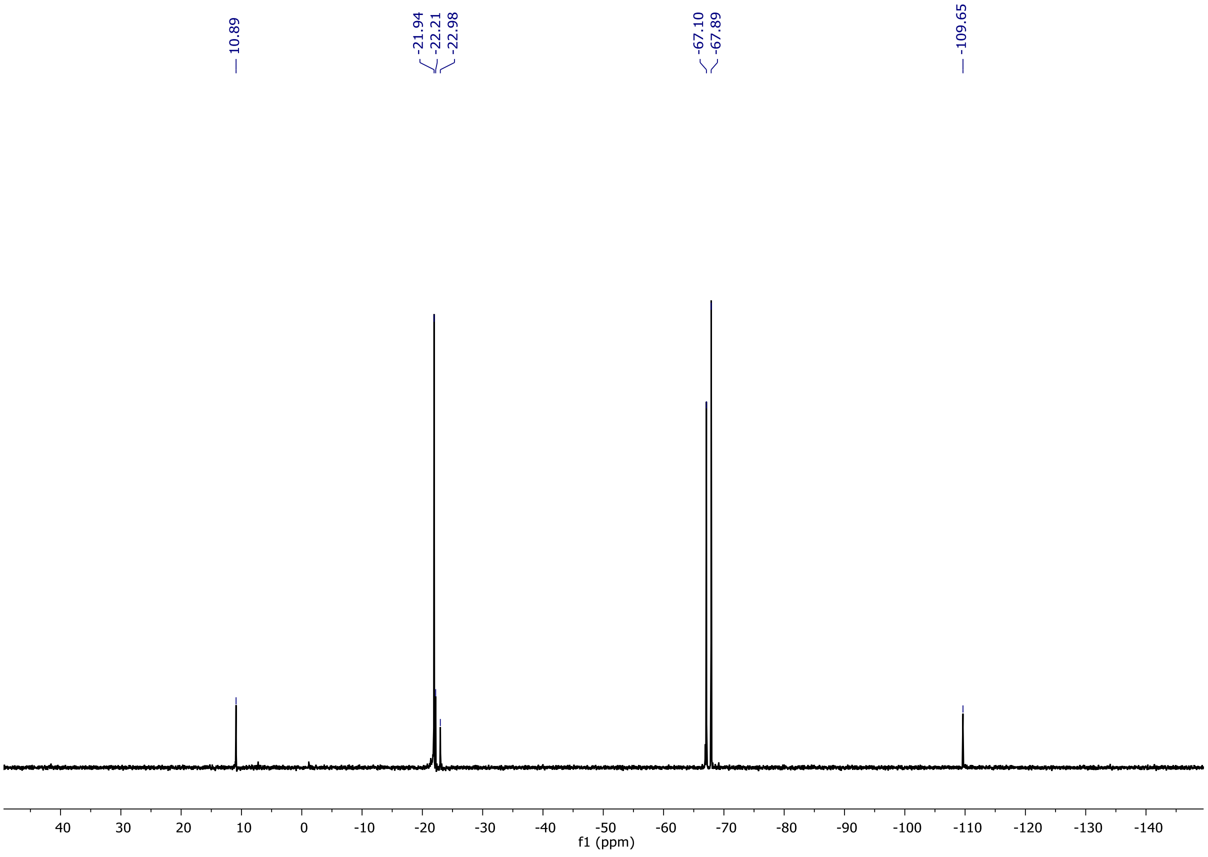

4-iBuT$_8$@PS

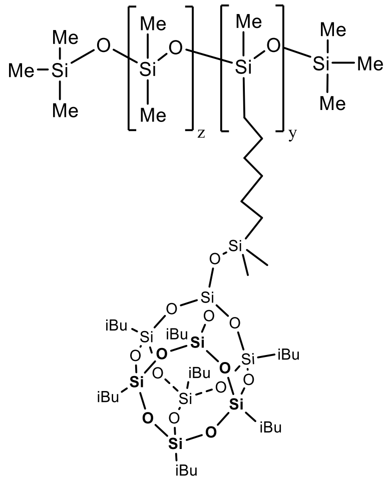

4-iBuT$_8$@PS1

^{1}H NMR (300.2 MHz, $CDCl_3$, δ, ppm): 0.05-0.09 (m, -SiC***H$_3$***), 0.50-0.62 (m, -C***H$_2$***, -C***H$_2$***- (iBu)), 0.94-0.97 (m, -C***H$_3$*** (iBu)), 1.30 (m, -C***H$_2$***-), 1.81-1.92 (m, -C***H***- (iBu)), 4.69 (s, -Si-***H***), 5.42-5.44 (m, -C***H***=C***H***- from by-product of bond isomerization). **^{13}C NMR** (100.6 MHz, $CDCl_3$, δ, ppm): -0.16, 1.22 (-Si***C***H$_3$), 17.76, 18.10, 23.21, 33.37, 33.52 (-***C***H$_2$-), 22.60-22.65, 24.00-24.04, 25.88 (iBu), 124.98 and 129.93 (-***C***H=***C***H- from by-product of bond isomerization). **^{29}Si NMR** (79.5 MHz, $CDCl_3$, δ, ppm): 11.36 (-***Si***-$(CH_2)_6$-Si-), -21.96, -22.23, -22.36 (-***Si***CH_3), -67.10, -67.89, -109.70 (-***Si***O_4).
FT-IR (cm^{-1}): 2953.14, 2923.48, 2868.95 (-C-H), 1464.53 (-C-H), 1258.85, 1228.75 (Si-C), 1077.23 (Si-O).

^{1}H NMR

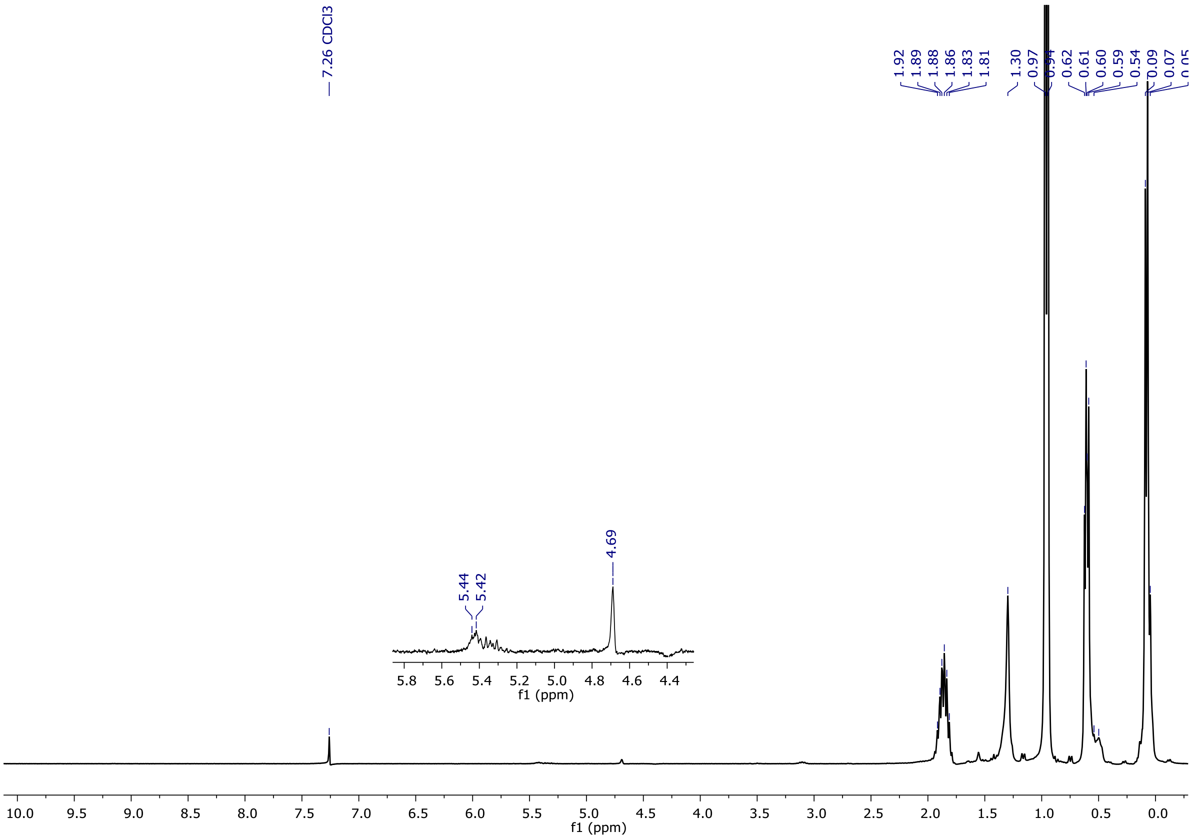

^{13}C NMR

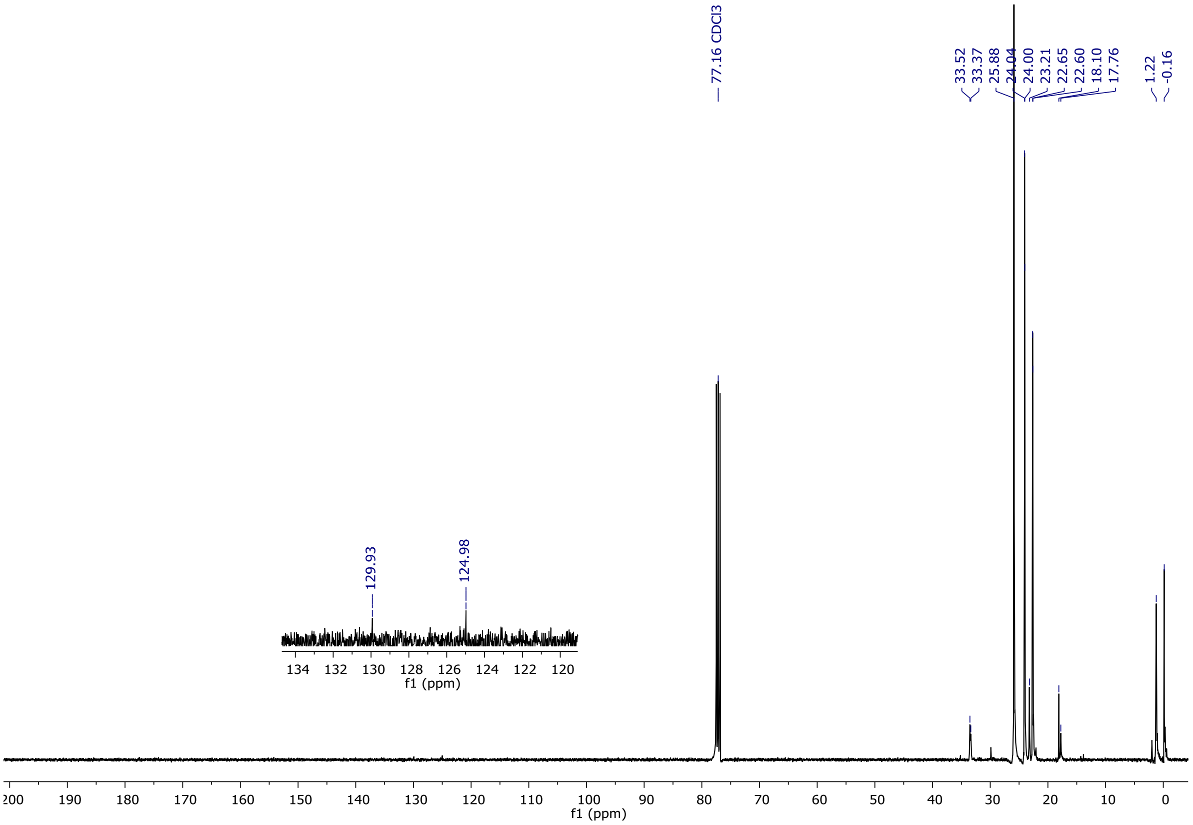

^{29}Si NMR

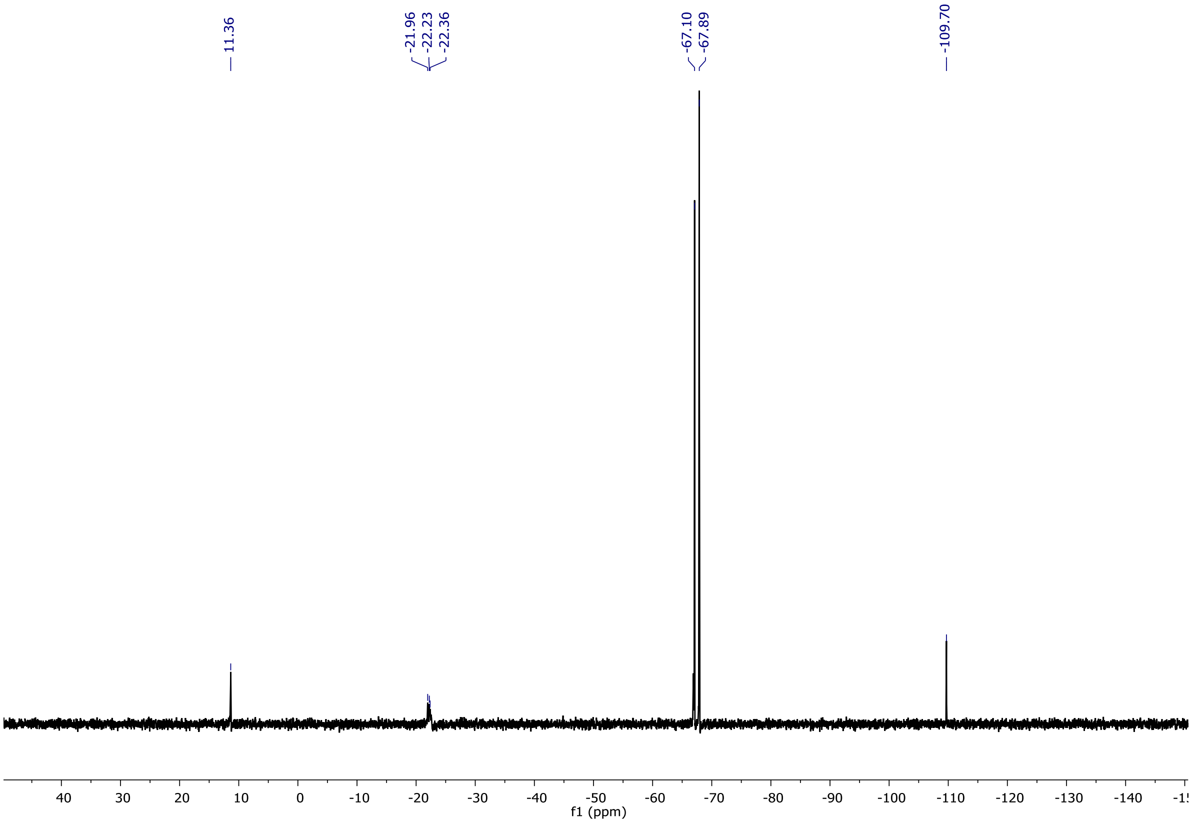

4-iBuT$_8$@PS2

^{1}H NMR (300.2 MHz, CDCl$_3$, δ, ppm): 0.07-0.09 (m, -SiC***H$_3$***), 0.47-0.62 (m, -C***H$_2$***, -C***H$_2$***- (iBu)), 0.94-0.97 (m, -C***H$_3$*** (iBu)), 1.30 (m, -C***H$_2$***-), 1.81-1.92 (m, -C***H***- (iBu)), 4.69 (s, -Si-***H***), 5.41-5.42 (m, -C***H***=C***H***- from by-product of bond isomerization). **^{13}C NMR** (100.6 MHz, CDCl$_3$, δ, ppm): -0.17, 1.21 (-Si***C***H$_3$), 17.75, 18.10, 23.21, 33.36, 33.52 (-***C***H$_2$-), 22.64-22.69, 24.00-24.04, 25.87 (iBu), 124.98 and 129.93 (-***C***H=***C***H- from by-product of bond isomerization). **^{29}Si NMR** (79.5 MHz, CDCl$_3$, δ, ppm): 11.37 (-***Si***-(CH$_2$)$_6$-Si-), -21.95, -22.01, -22.24, -22.37 (-***Si***CH$_3$), -67.09, -67.89, -109.69 (-***Si***O$_4$).
FT-IR (cm^{-1}): 2955.88, 2924.57, 2870.22 (-C-H), 1465.08 (-C-H), 1258.60, 1228.59 (Si-C), 1081.19, 1013.48 (Si-O).

^{1}H NMR

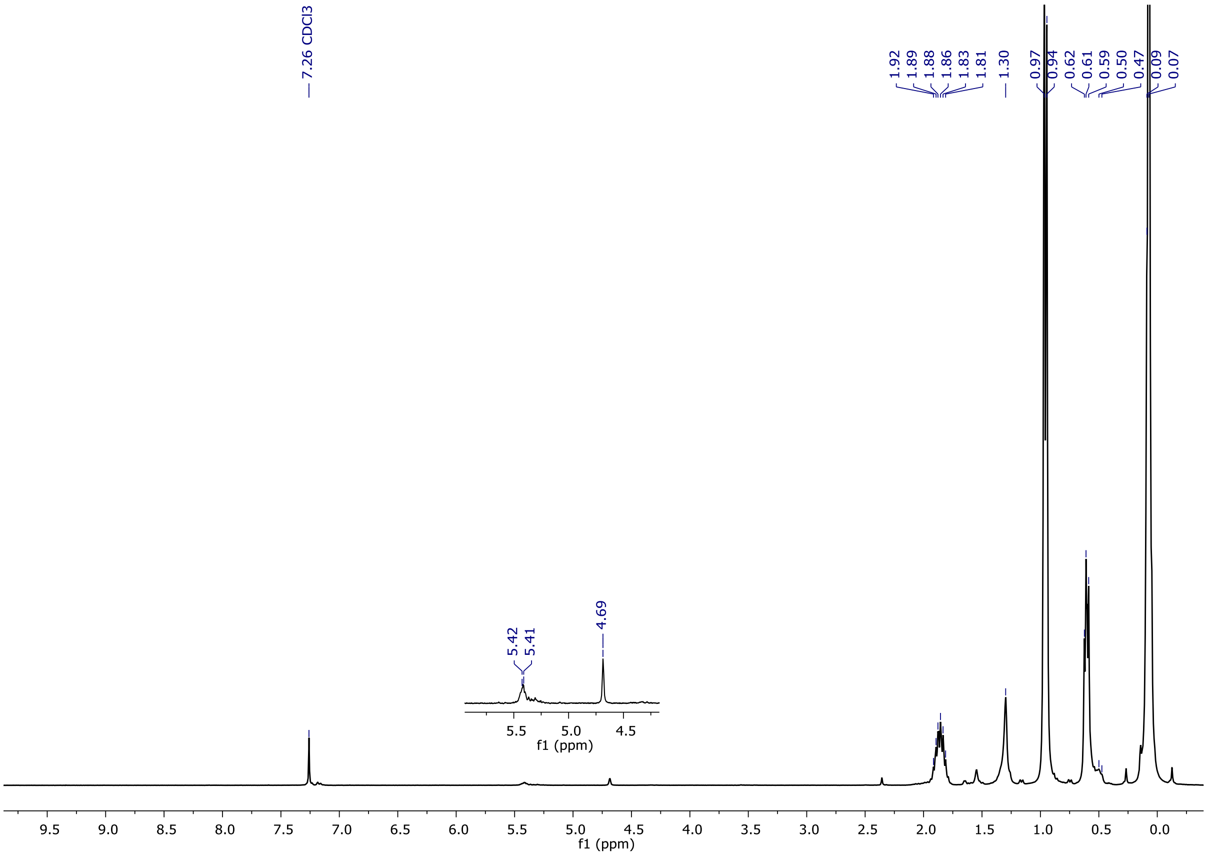

^{13}C NMR

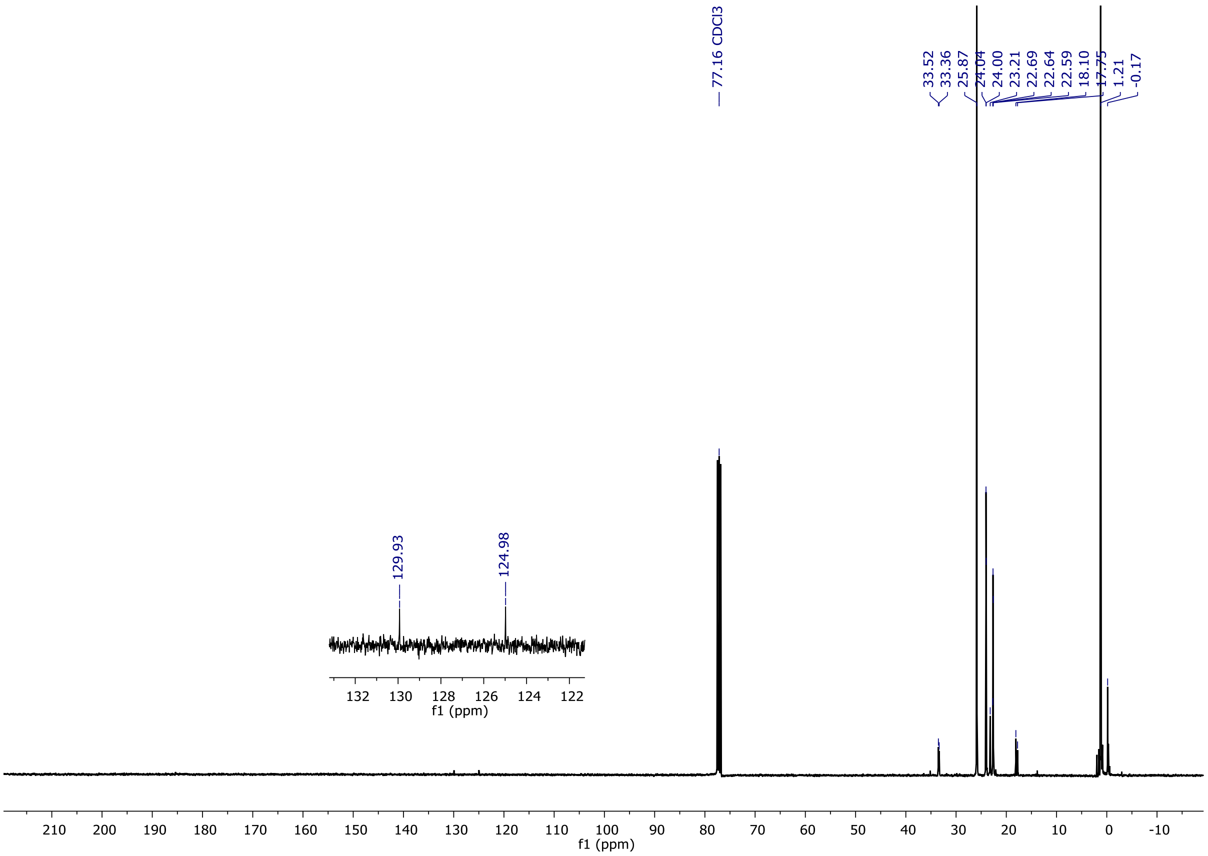

^{29}Si NMR

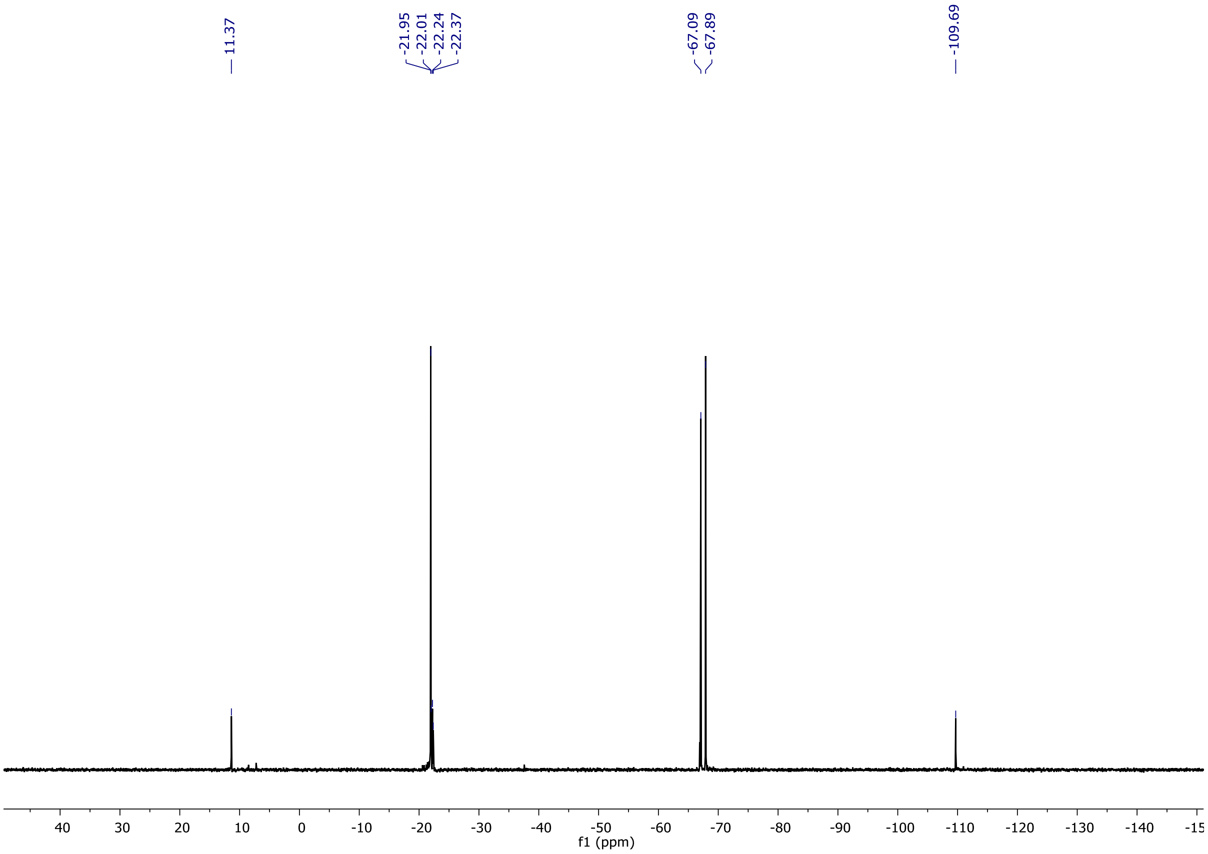

5-iBuT_8@PS

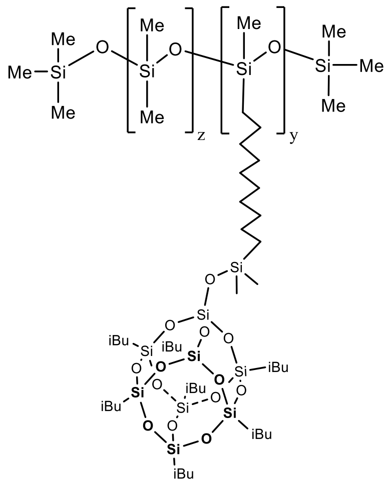

5-iBuT_8@PS1

^{1}H NMR (300.2 MHz, $CDCl_3$, δ, ppm): 0.07-0.09 (m, -SiC***H_3***), 0.49-0.62 (m, -C***H_2***-, -C***H_2***- (iBu)), 0.94-0.96 (m, -C***H_3*** (iBu)), 1.26-1.29 (m, -C***H_2***-), 1.81-1.91 (m, -C***H***- (iBu)), 4.68 (s, -Si-***H***), 5.38- 5.41 (m, -C***H***=C***H***- from by-product of bond isomerization). **^{13}C NMR** (100.6 MHz, $CDCl_3$, δ, ppm): -0.13, 1.22 (-Si***C***H_3), 18.07, 23.25, 29.99, 33.77 (-***C***H_2-), 22.60-22.65, 24.05, 25.88 (iBu), 125.48 and 129.20 (-***C***H=***C***H- from by-product of bond isomerization). **^{29}Si NMR** (79.5 MHz, $CDCl_3$, δ, ppm): 11.38 (-***Si***-$(CH_2)_{10}$-Si-), -21.94, -22.29, (-***Si***CH_3), -67.10, -67.89, -109.70 (-***Si***O_4).
FT-IR (cm^{-1}): 2953.13, 2923.21, 2868.37 (-C-H), 1464.53 (-C-H), 1258.84, 1228.88 (Si-C), 1081.40 (Si-O).

^{1}H NMR

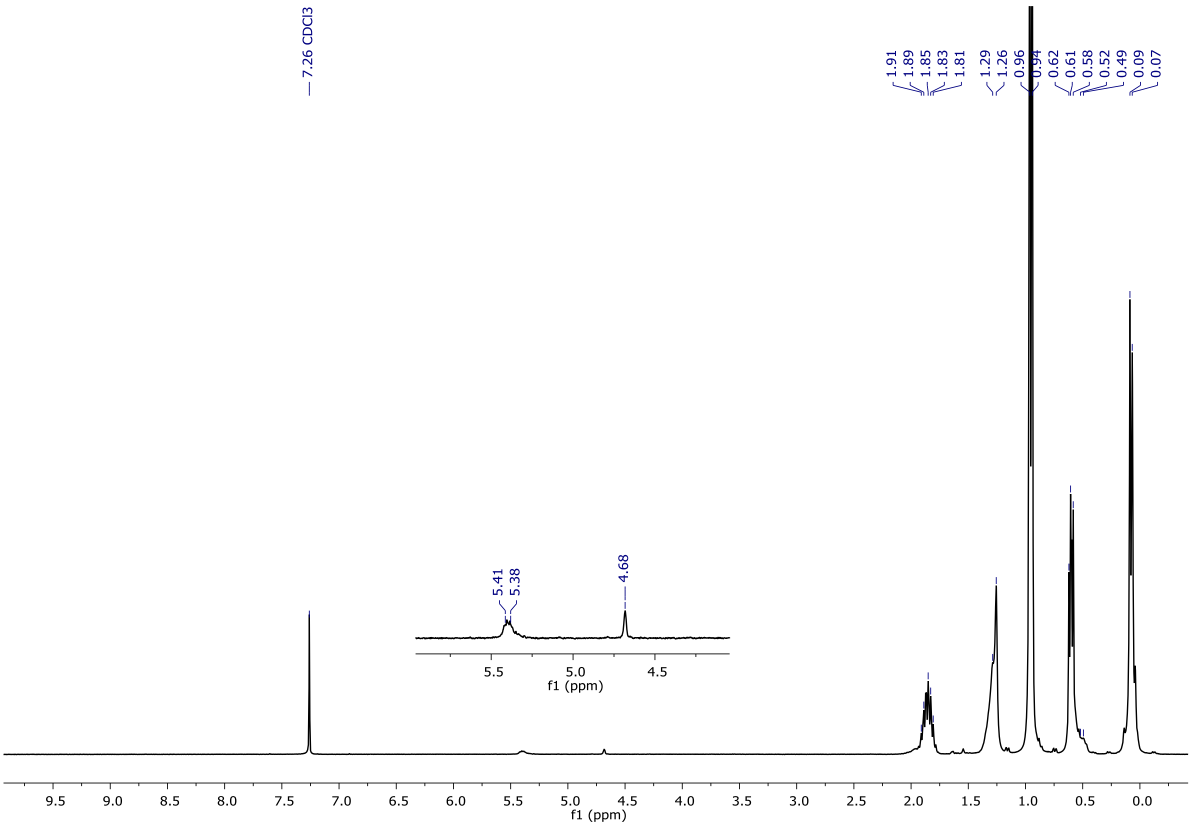

^{13}C NMR

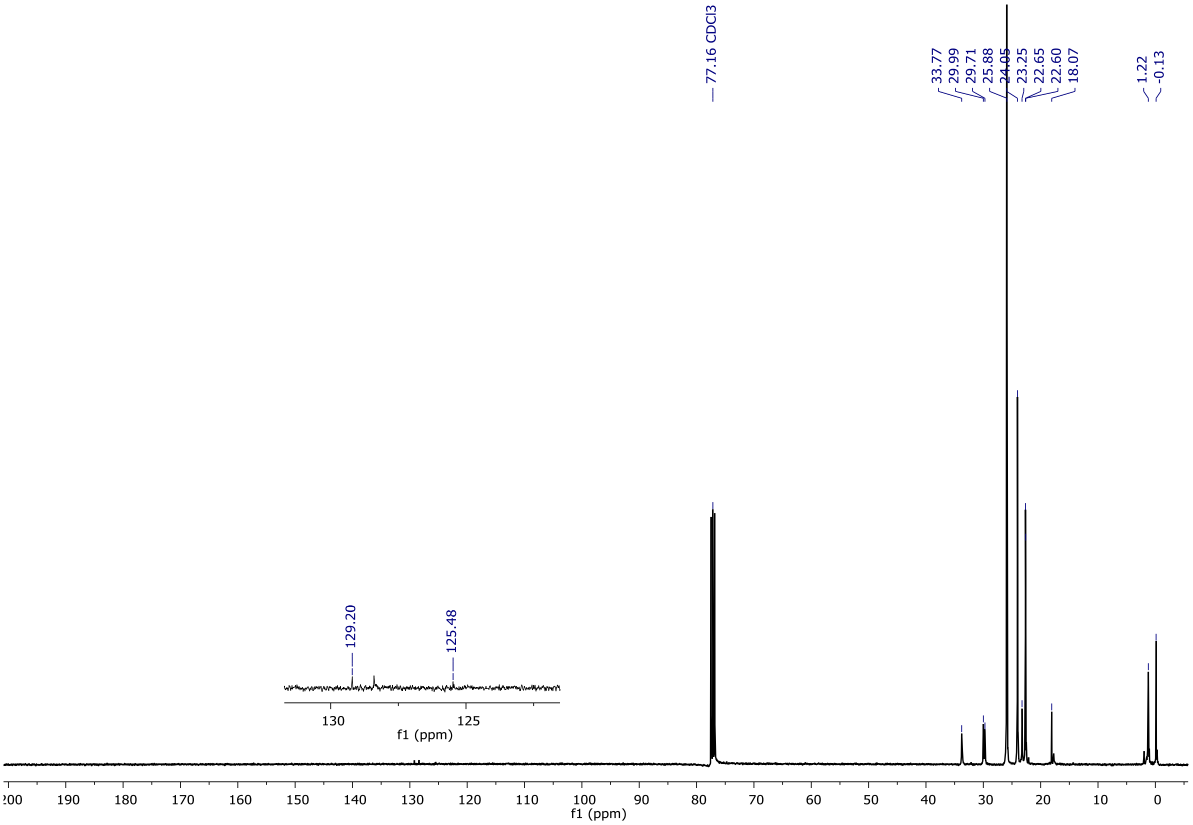

^{29}Si NMR

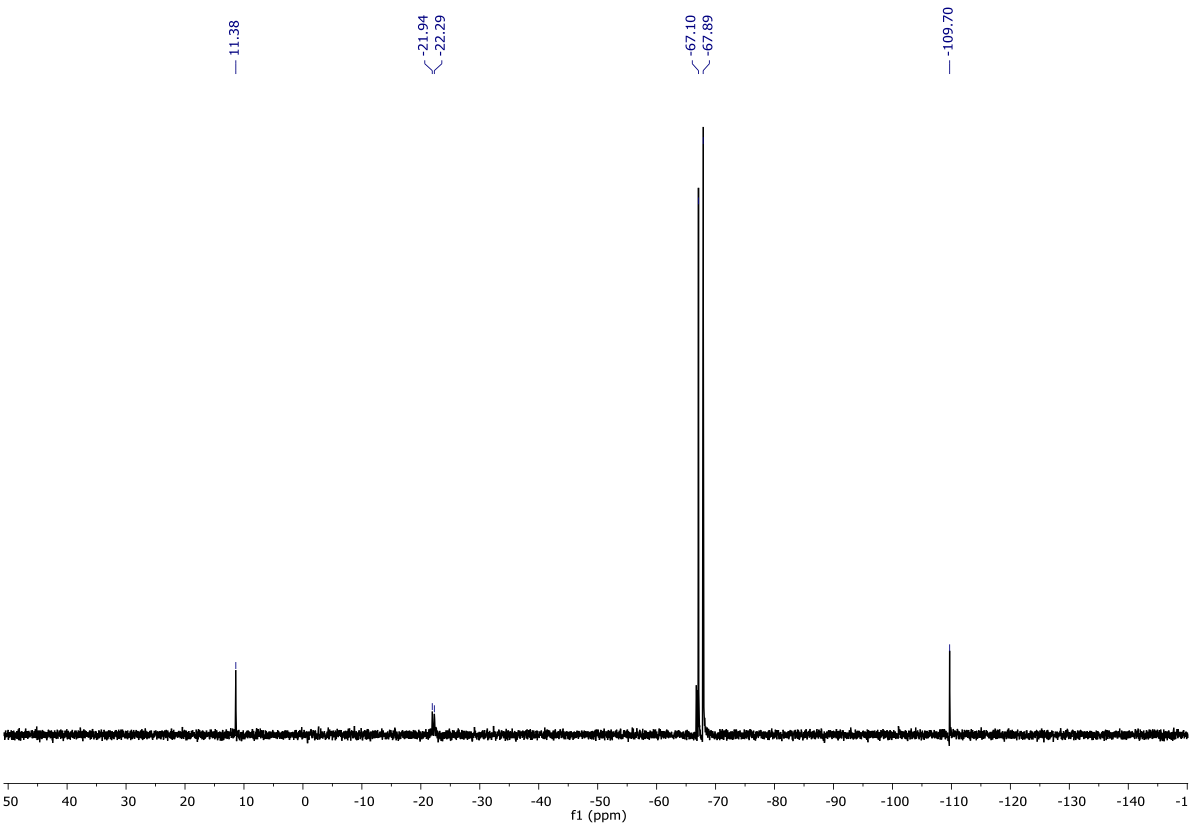

5-iBuT$_8$@PS2

^{1}H NMR (300.2 MHz, $CDCl_3$, δ, ppm): 0.07-0.09 (m, -SiC***H***$_3$), 0.59-0.63 (m, -C***H***$_2$-, -C***H***$_2$- (iBu)), 0.94-0.97 (m, -C***H***$_3$ (iBu)), 1.26-1.30 (m, -C***H***$_2$-), 1.81-1.92 (m, -C***H***- (iBu)), 4.68 (s, -Si-***H***), 5.39- 5.41 (m, -C***H***=C***H***- from by-product of bond isomerization). **^{13}C NMR** (100.6 MHz, $CDCl_3$, δ, ppm): -0.15, 1.20, 1.25 (-Si***C***H$_3$), 17.69, 18.05, 23.22, 29.71-29.96, 33.64-33.75 (-***C***H$_2$-), 22.58-22.67, 23.99-24.02, 25.85 (iBu), 123.21 and 129.54 (-***C***H=***C***H- from by-product of bond isomerization). **^{29}Si NMR** (79.5 MHz, $CDCl_3$, δ, ppm): 11.40 (-***Si***-$(CH_2)_{10}$-Si-), -21.94, -22.24, (-***Si***CH$_3$), -67.10, -67.89, -109.70 (-***Si***O$_4$).
FT-IR (cm^{-1}): 2955.17, 2923.92, 2855.19 (-C-H), 1464.88 (-C-H), 1258.66, 1228.51 (Si-C), 1083.35, 1012.68 (Si-O).

^{1}H NMR

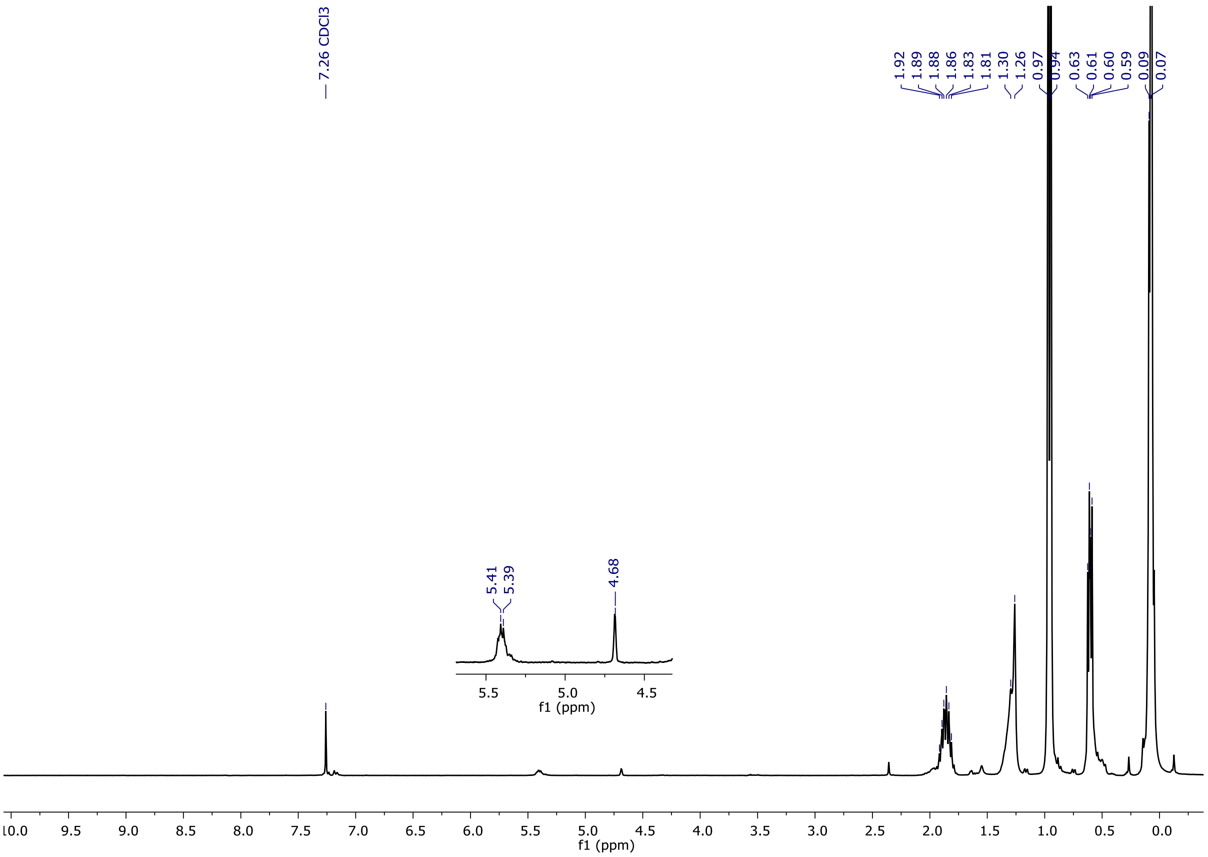

^{13}C NMR

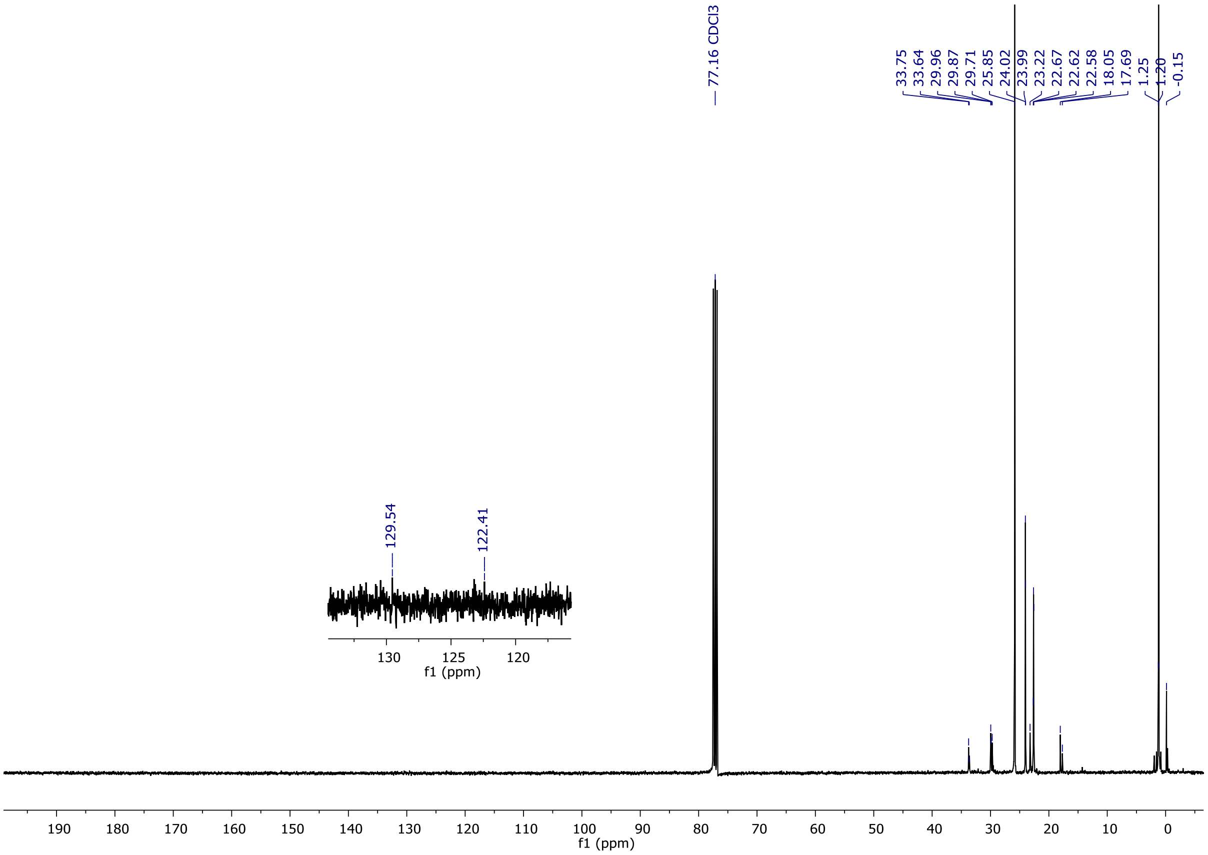

^{29}Si NMR

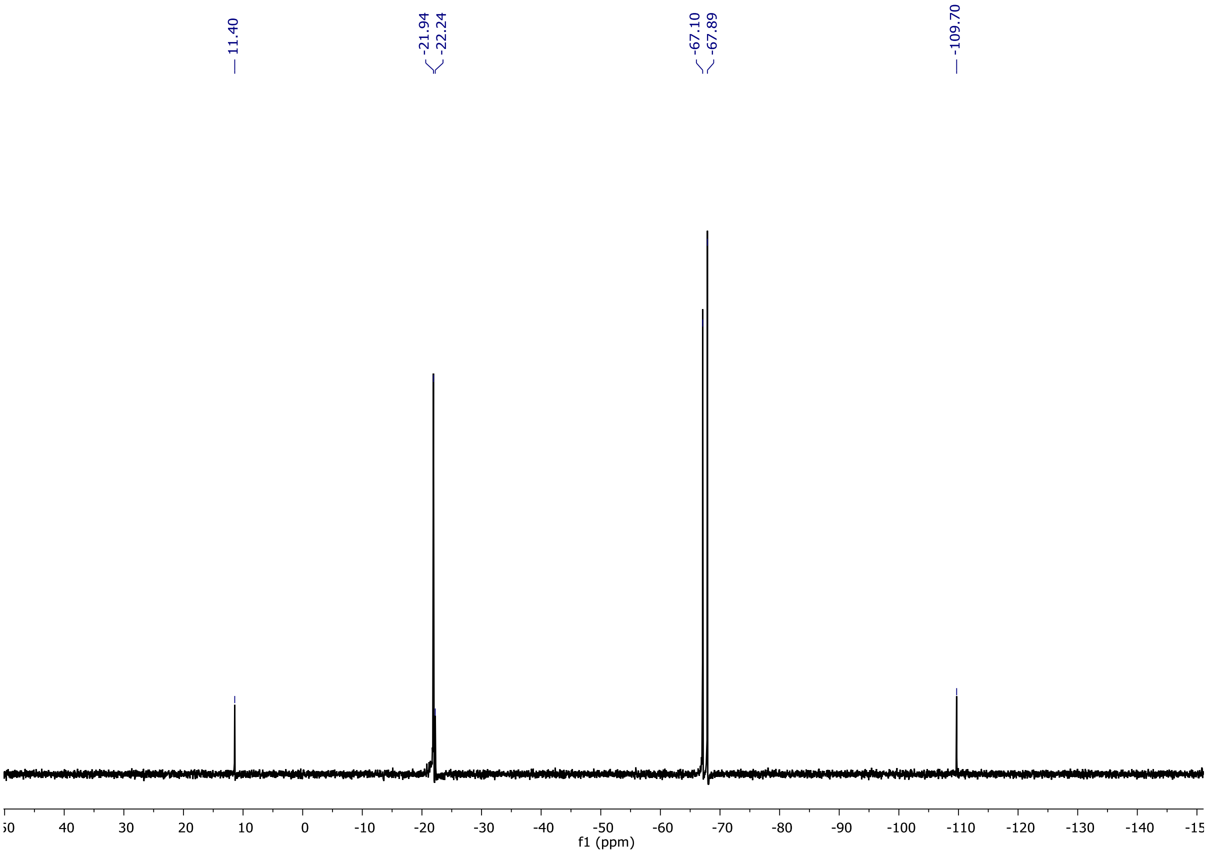

1-PhT$_8$@PS

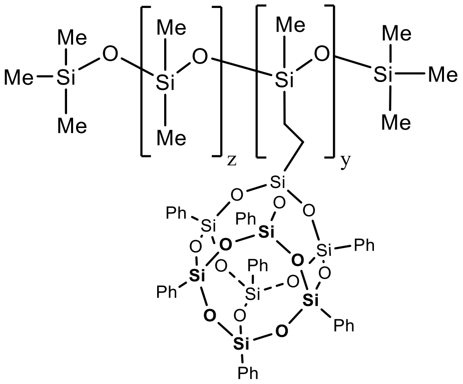

1-PhT$_8$@PS1

^{1}H NMR (300.2 MHz, $CDCl_3$, δ, ppm): -0.03, 0.00 (m, -SiC***H*$_3$**), 0.58-0.78 (m, -C***H*$_2$**-), 7.32-7.35, 7.72 (m, **Ph**). **^{13}C NMR** (100.6 MHz, $CDCl_3$, δ, ppm): -1.27, 1.21, 1.95 (-Si***C***H_3), 3.69, 8.58 (-***C***H_2-), 127.99, 130.45, 130.88, 134.35 (Ph). **^{29}Si NMR** (79.5 MHz, $CDCl_3$, δ, ppm): -21.82, -22.59 (-***Si***CH_3), -64.50 (-***Si***-CH_2-CH_2-Si-), -78.29, -78.57, -78.80.
FT-IR (cm^{-1}): 3073.39, 3051.43, 3028.85 (C-H phenyl), 2959.68 (-C-H), 1594.37 (C=C phenyl), 1489.64 (-C-H), 1430.53 (C=C phenyl), 1259.53 (Si-C), 1080.27, 1011.50 (Si-O), 996.80 (C-H phenyl).

^{1}H NMR

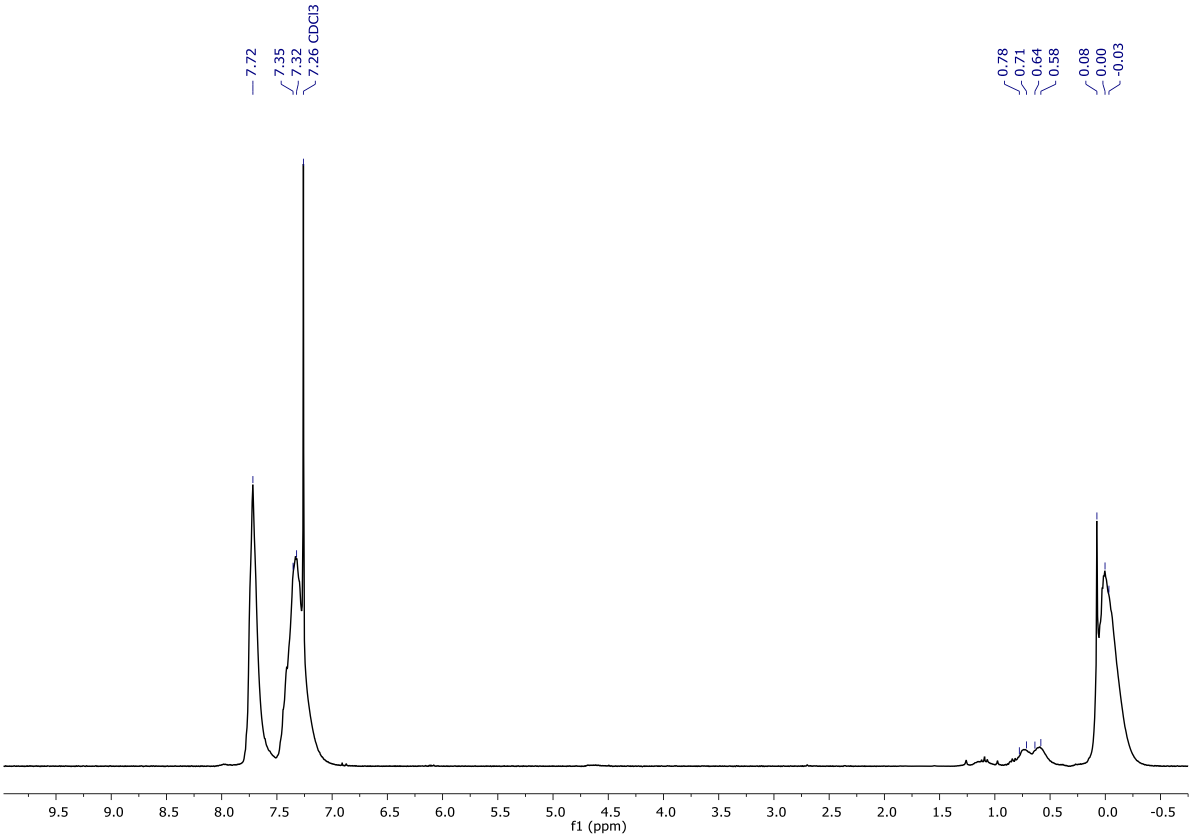

^{13}C NMR

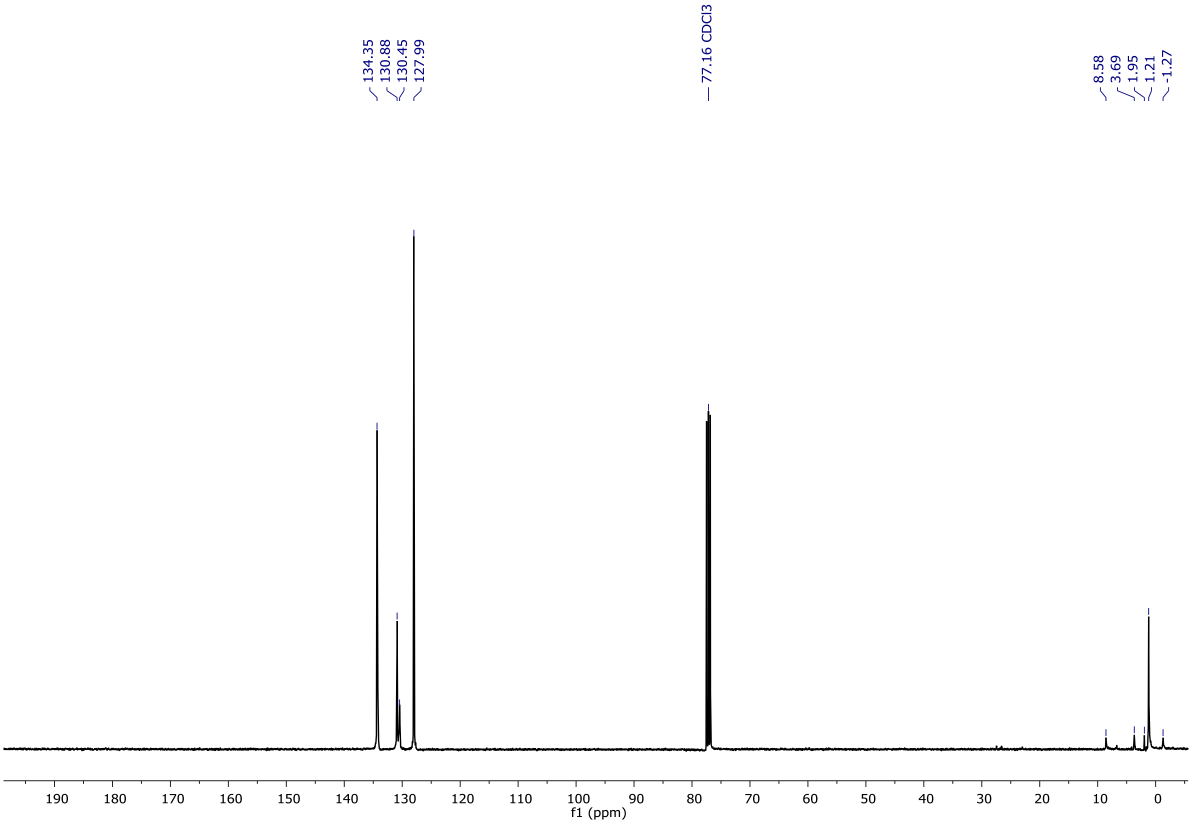

^{29}Si NMR

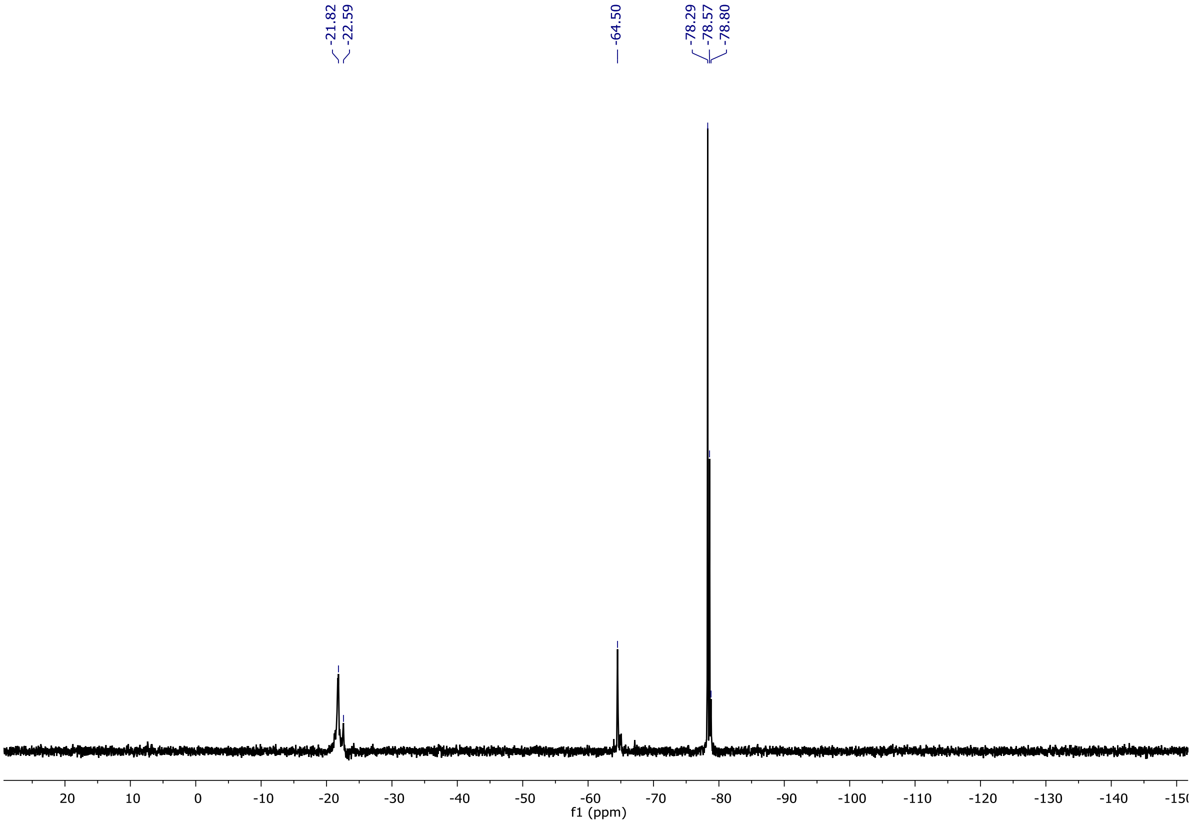

1-PhT$_8$@PS2

^{1}H NMR (300.2 MHz, $CDCl_3$, δ, ppm): 0.01-0.08 (m, -SiC***H$_3$***), 0.60-0.80 (m, -C***H$_2$***-), 7.34-7.42, 7.73-7.74 (m, **Ph**). **^{13}C NMR** (100.6 MHz, $CDCl_3$, δ, ppm): -1.28, 1.19 (-Si***C***H$_3$), 3.69, 8.59 (-***C***H$_2$-), 127.98, 130.48, 130.85, 134.30, 134.35 (Ph). **^{29}Si NMR** (79.5 MHz, $CDCl_3$, δ, ppm): -21.89 (-***Si***CH$_3$), -64.53 (-***Si***-CH$_2$-CH$_2$-Si-), -78.36, -78.67.
FT-IR (cm^{-1}): 3073.78, 3052.14, 3028.97 (C-H phenyl), 2961.02, 2924.80 (-C-H), 1594.64 (C=C phenyl), 1489.55 (-C-H), 1430.86 (C=C phenyl), 1259.35 (Si-C), 1134.63, 1092.26, 1017.15 (Si-O).

^{1}H NMR

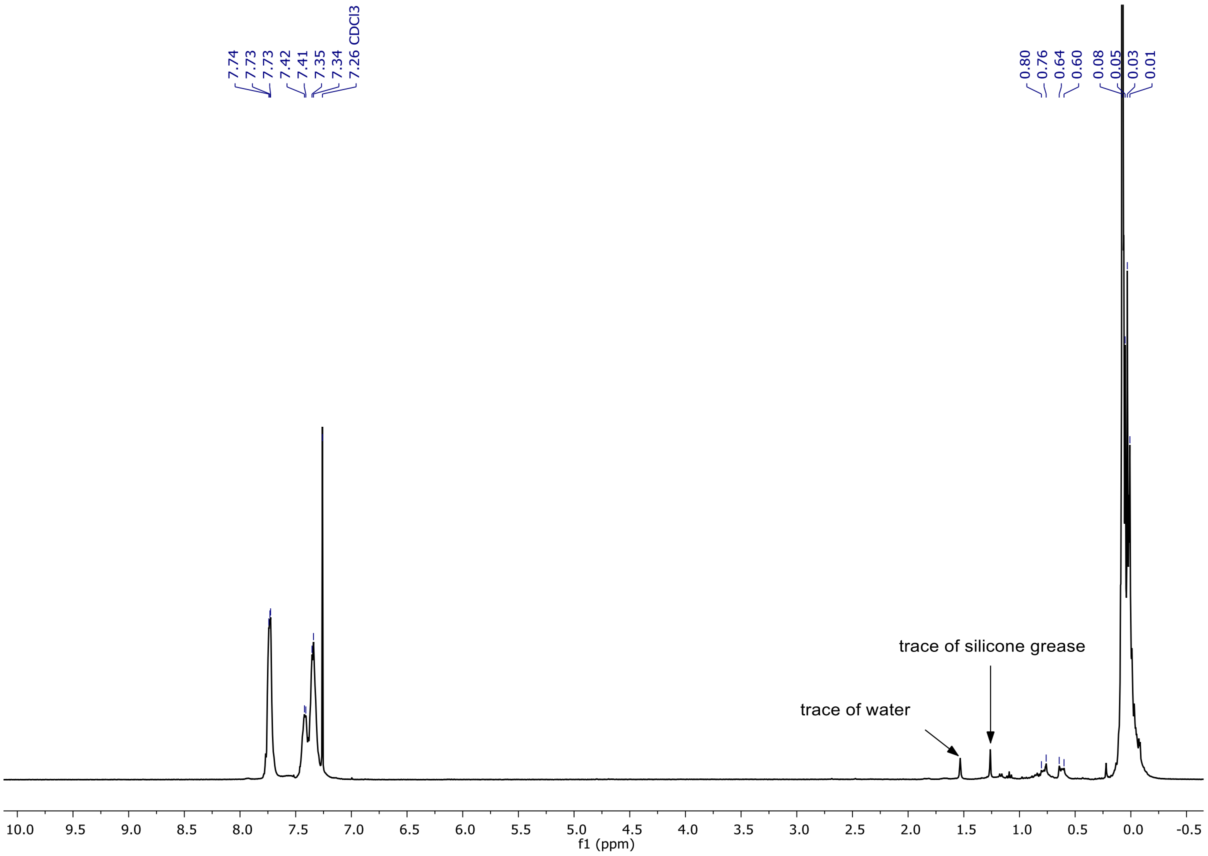

^{13}C NMR

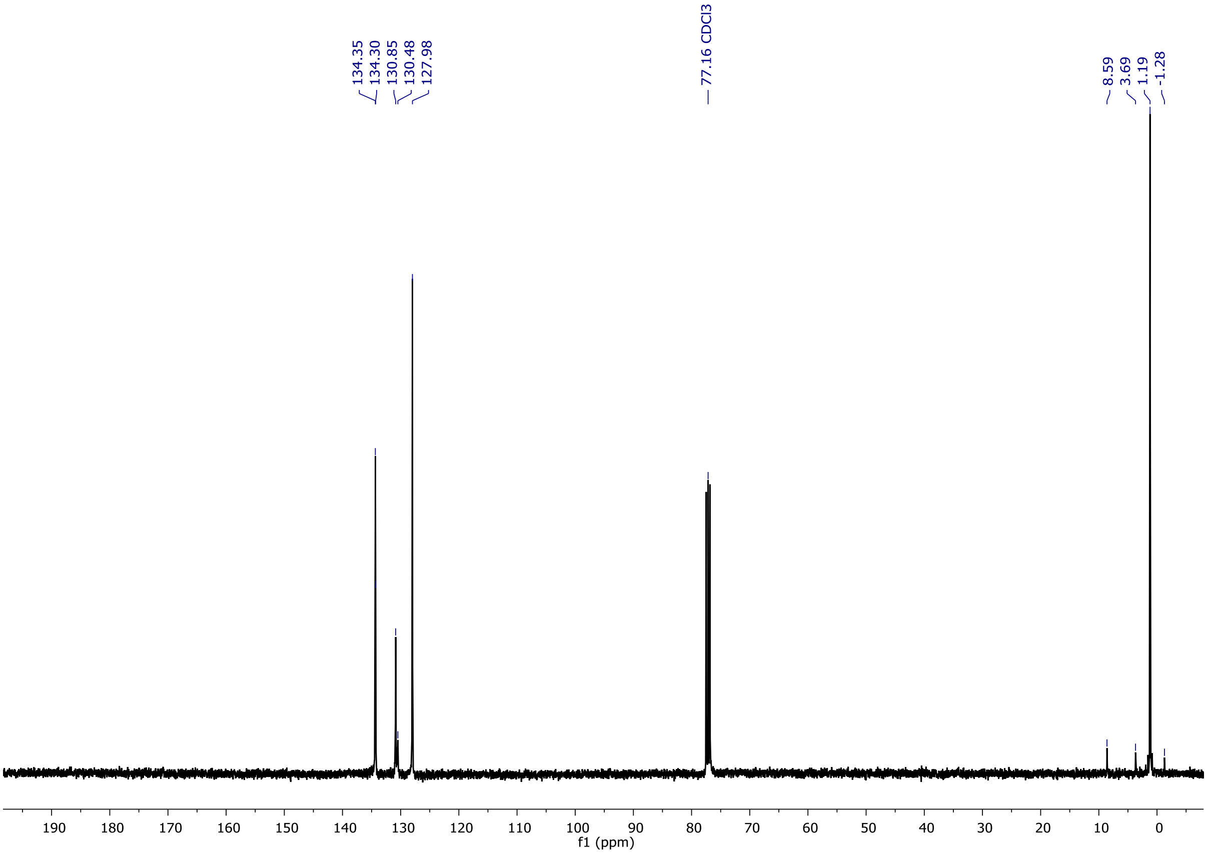

^{29}Si NMR

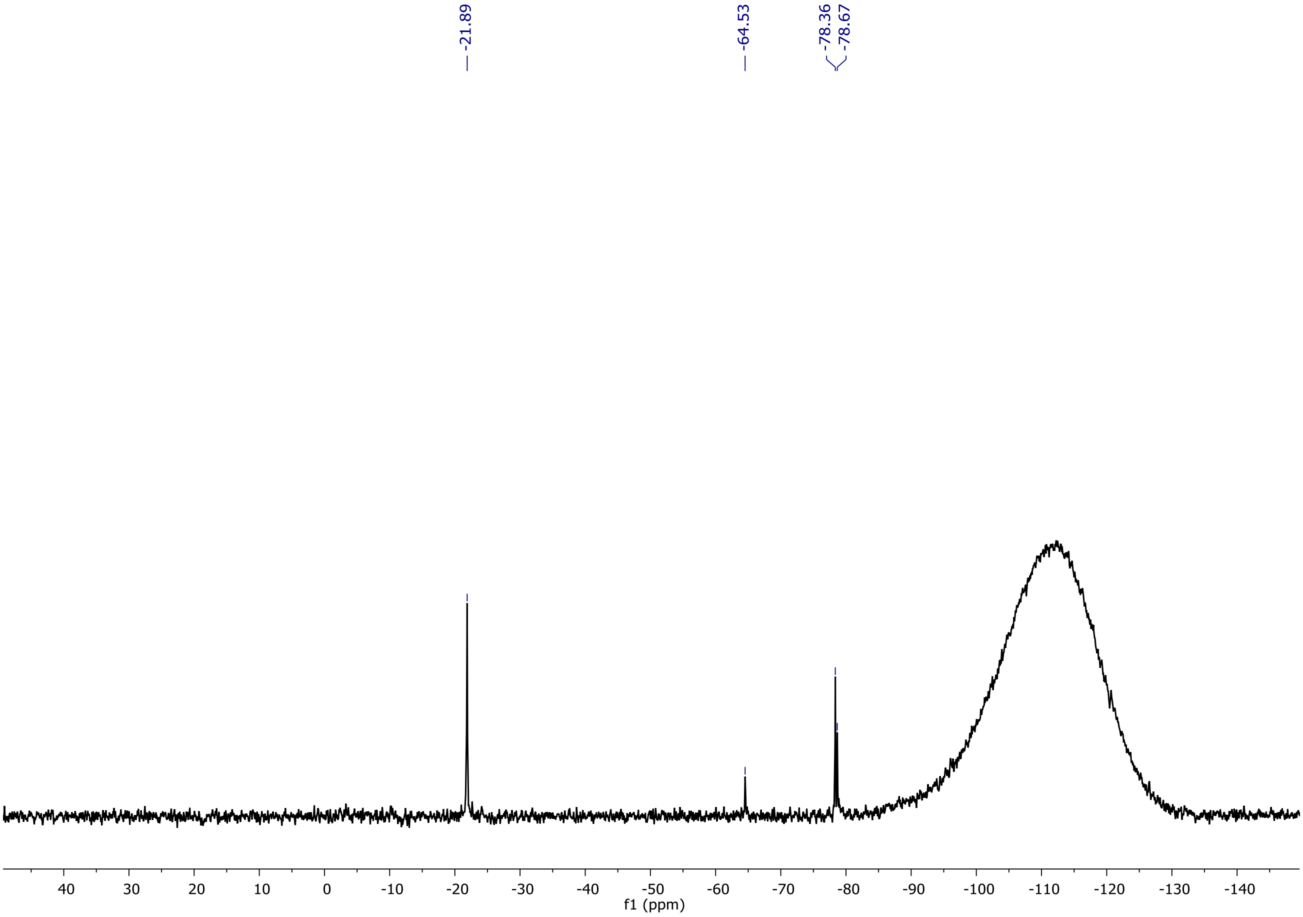

2-PhT$_8$@PS

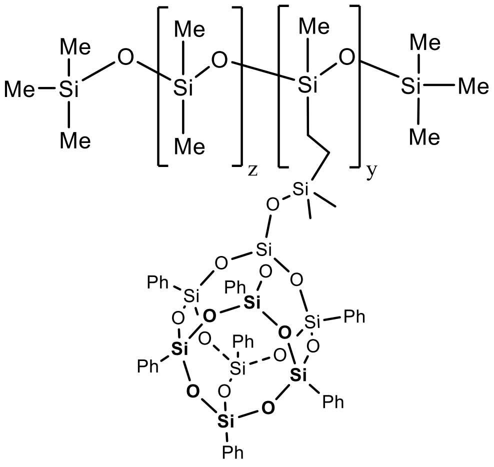

2-PhT$_8$@PS1

^{1}H NMR (300.2 MHz, $CDCl_3$, δ, ppm): -0.04-0.08 (m, -SiC***H$_3$***), 0.33-0.50 (m, -C***H$_2$***-), 7.32-7.38, 7.71-7.74 (m, **Ph**). **^{13}C NMR** (100.6 MHz, $CDCl_3$, δ, ppm): -0.93, 1.22, 1.96 (-Si***C***H$_3$), 8.58, 8.92 (-***C***H$_2$-), 128.00, 130.31, 130.91, 134.33 (Ph). **^{29}Si NMR** (79.5 MHz, $CDCl_3$, δ, ppm): 13.68 (-***Si***-CH$_2$-CH$_2$-Si-), -21.59, -21.87, -22.65 (-***Si***CH$_3$), -78.09, -78.26, -78.32, 108.82 (-***Si***O$_4$).
FT-IR (cm^{-1}): 3073.50, 3051.33 (C-H phenyl), 2959.22 (-C-H), 1594.37 (C=C phenyl), 1489.75 (-C-H), 1430.49 (C=C phenyl), 1258.99 (Si-C), 1131.72, 1079.97, 1026.73 (Si-O), 997.06 (C-H phenyl).

^{1}H NMR

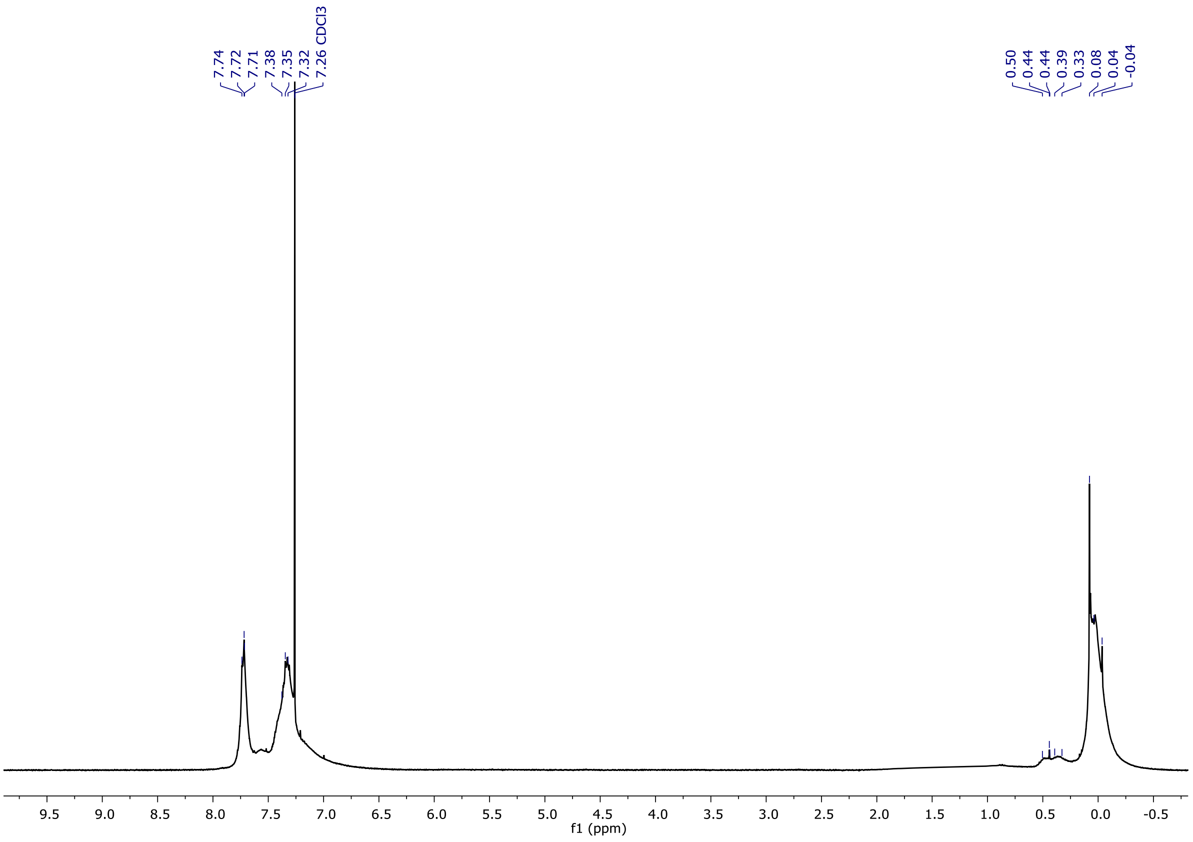

^{13}C NMR

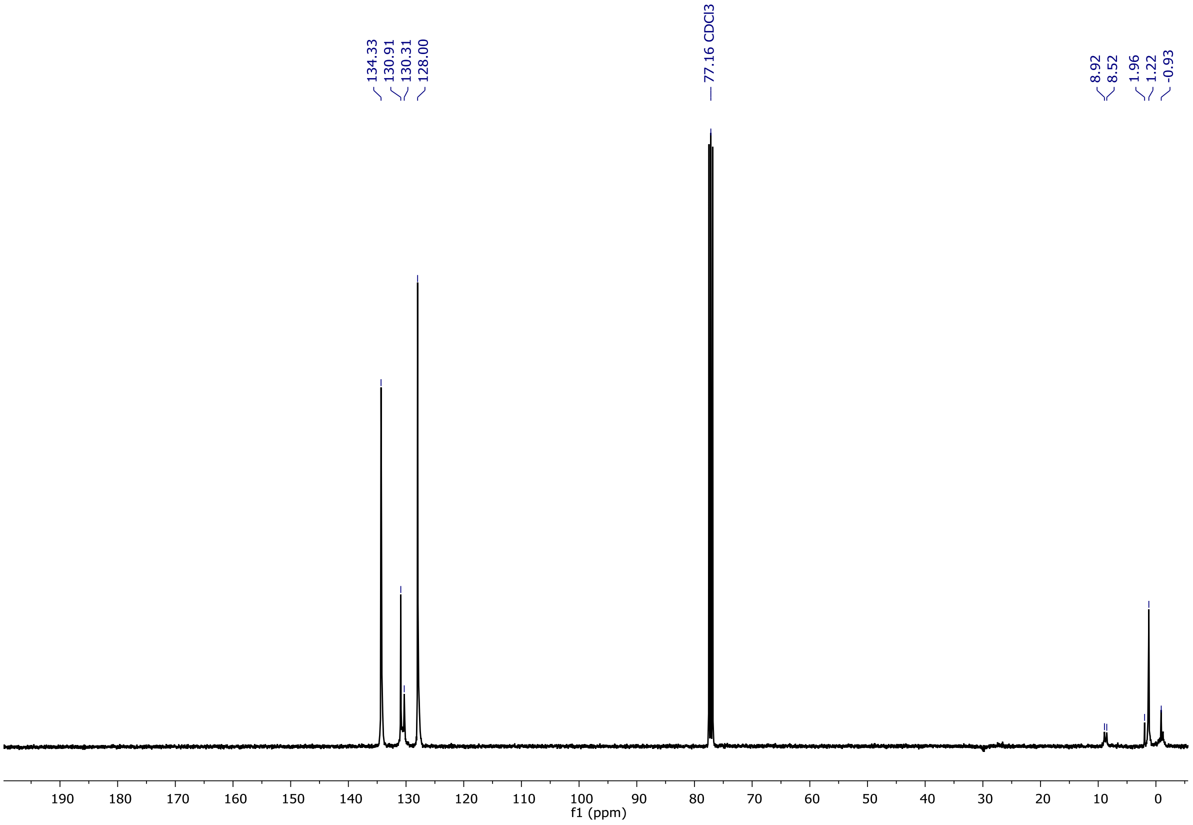

^{29}Si NMR

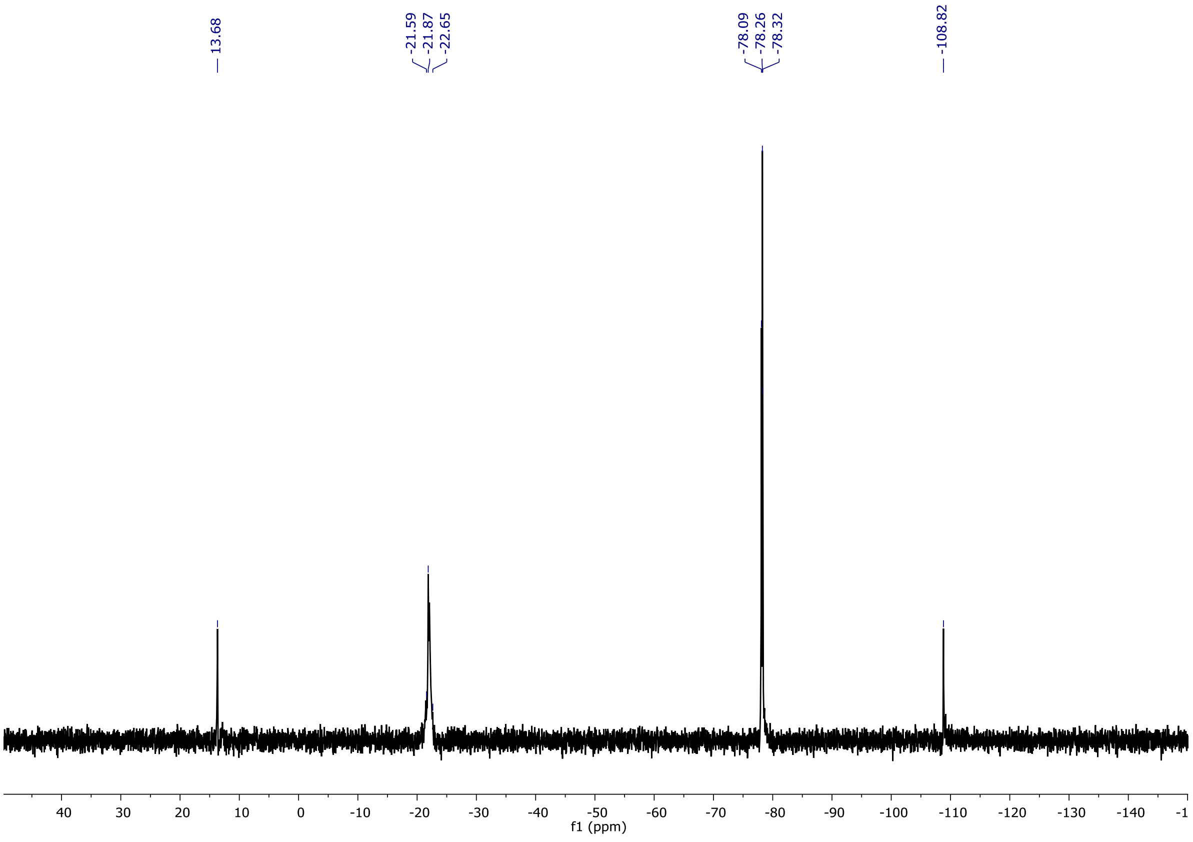

2-PhT$_8$@PS2

^{1}H NMR (300.2 MHz, $CDCl_3$, δ, ppm): -0.04-0.08 (m, -SiC***H$_3$***), 0.35-0.53 (m, -C***H$_2$***-), 7.32-7.43, 7.72-7.76 (m, **Ph**). **^{13}C NMR** (100.6 MHz, $CDCl_3$, δ, ppm): -0.91, 1.20, 1.95 (-Si***C***H$_3$), 8.51, 8.91 (-***C***H$_2$-), 128.00, 130.33, 130.90, 134.34 (Ph). **^{29}Si NMR** (79.5 MHz, $CDCl_3$, δ, ppm): 13.66 (-***Si***-CH$_2$-CH$_2$-Si-), -21.90, -22.06 (-***Si***CH$_3$), -78.14, -78.31, -78.36, 108.87 (-***Si***O$_4$).
FT-IR (cm^{-1}): 3073.90, 3051.76 (C-H phenyl), 2961.02 (-C-H), 1594.59 (C=C phenyl), 1430.90 (C=C phenyl), 1259.67 (Si-C), 1134.91, 1091.72, 1019.75 (Si-O).

^{1}H NMR

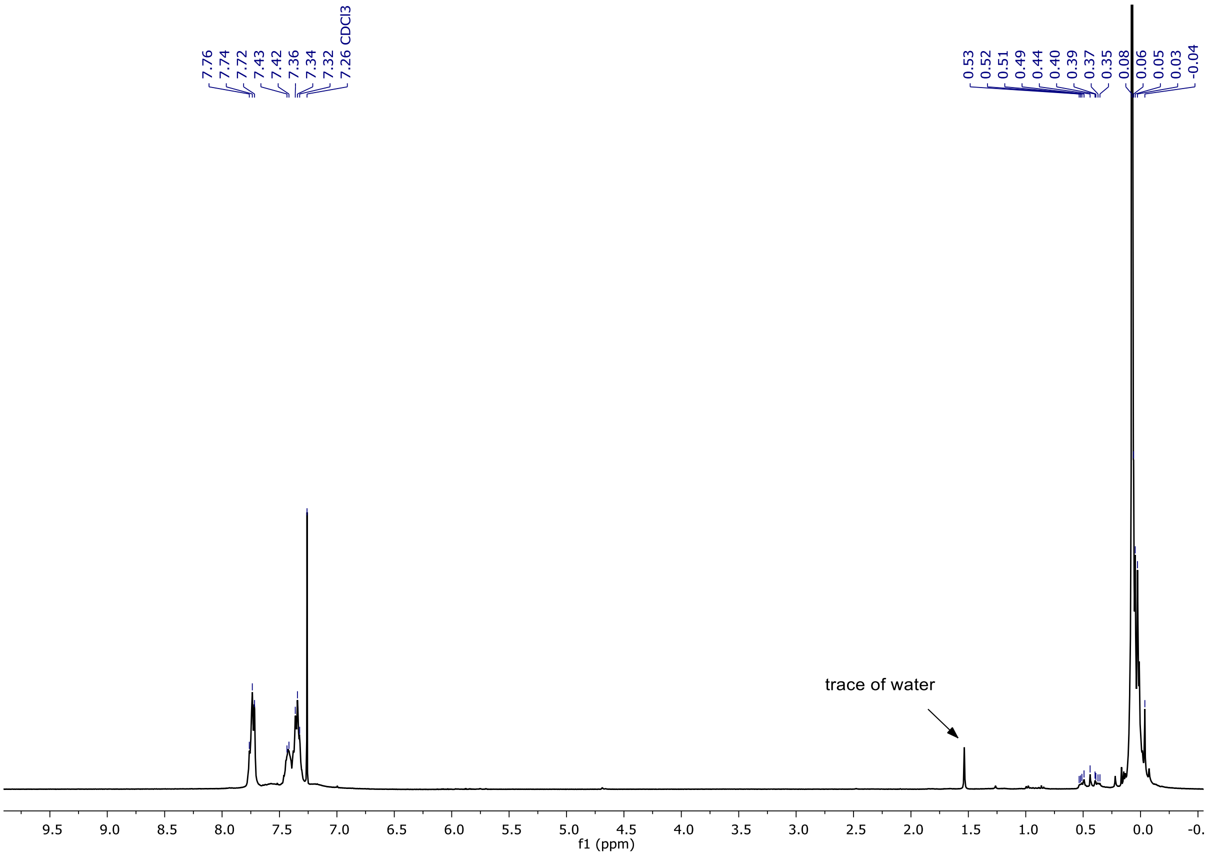

^{13}C NMR

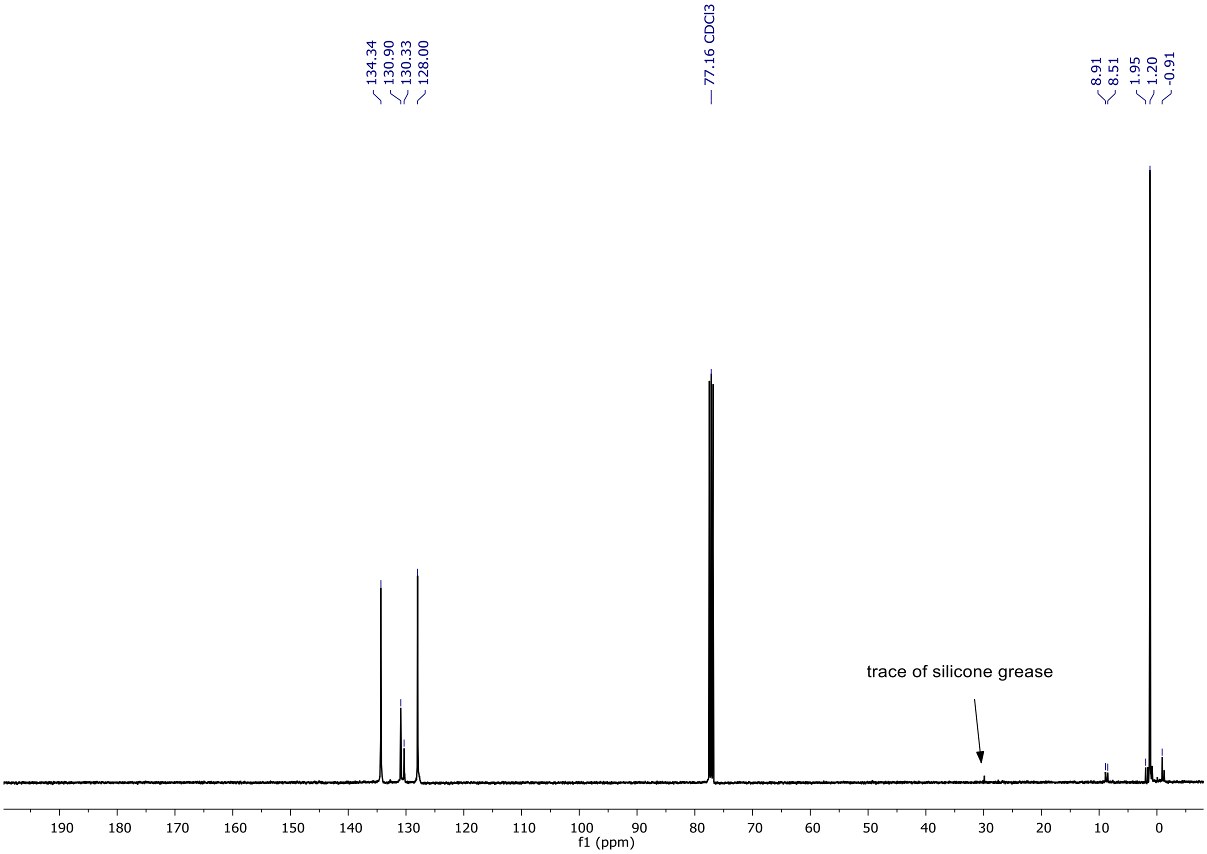

^{29}Si NMR

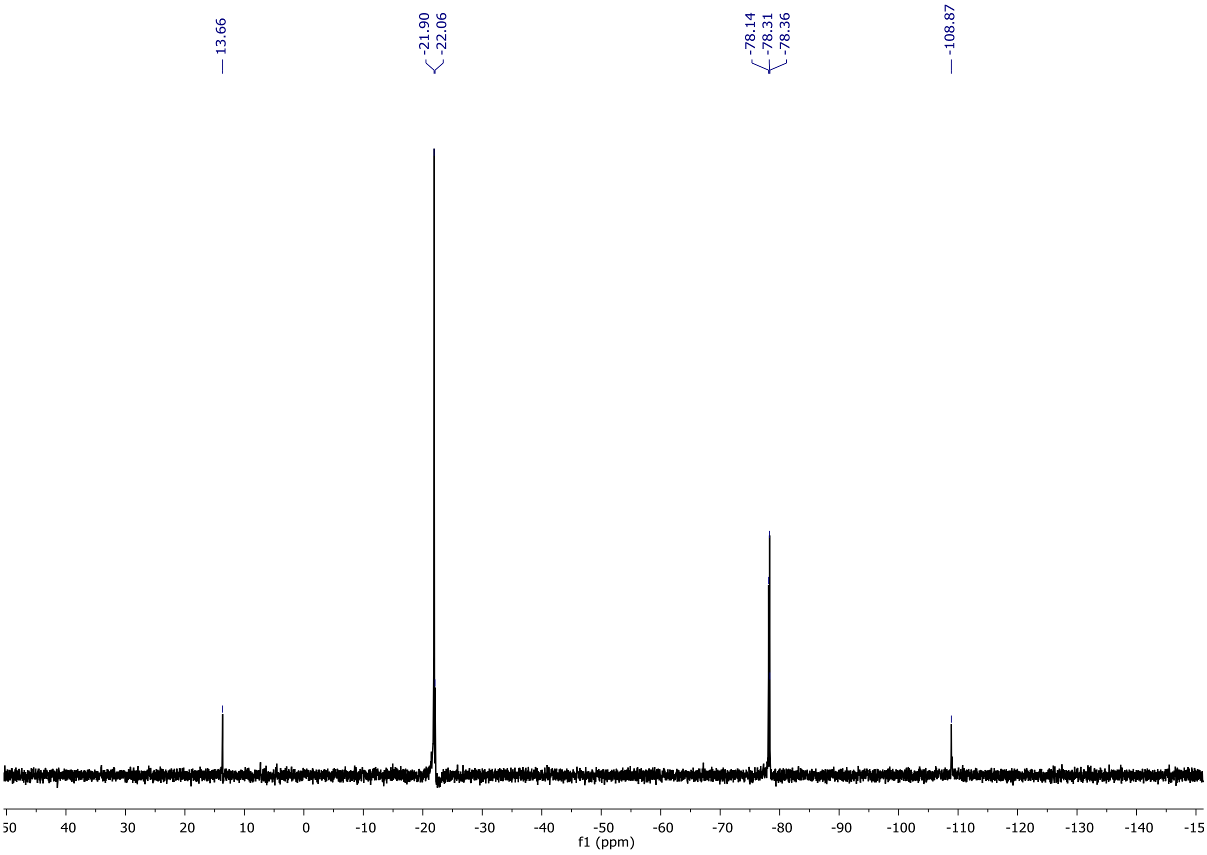

3-PhT$_8$@PS

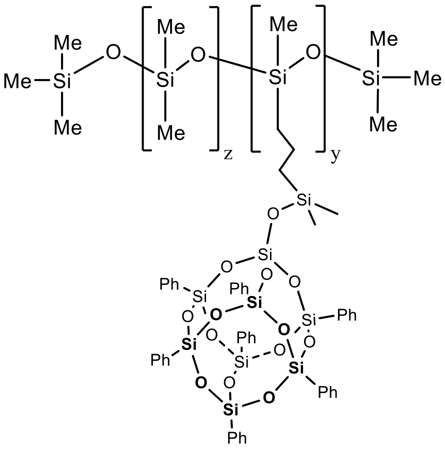

3-PhT$_8$@PS1

^{1}H NMR (300.2 MHz, $CDCl_3$, δ, ppm): 0.05-0.15 (m, -SiC***H$_3$***), 0.44-0.47, 0.62-0.64, 1.32-1.35 (m, -C***H$_2$***-), 1.66-1.69, 5.57-5.63 and 6.10-6.18 (m, -C***H$_2$***-C***H***=C***H***- from dehydrogenative silylation by-product), 7.33-7.45, 7.72-7.79 (m, **Ph**). **^{13}C NMR** (100.6 MHz, $CDCl_3$, δ, ppm): -0.12, 0.27, 1.20, 1.96 (-Si***C***H_3), 16.81, 21.81, 22.01 (-***C***H_2-), 128.01, 130.30, 130.92, 134.34 (Ph), 26.86, 129.76 and 144.24 (-***C***H_2-***C***H=***C***H- from dehydrogenative silylation by-product). **^{29}Si NMR** (79.5 MHz, $CDCl_3$, δ, ppm): 12.64 (-***Si***-CH_2-CH_2-CH_2-Si-), -21.89, -21.16, -22.65 (-***Si***CH_3), -78.13, -78.29, -78.35, 108.88 (-***Si***O_4). **FT-IR** (cm^{-1}): 3073.49, 3051.39 (C-H phenyl), 2959.76, 2919.00 (-C-H), 1594.29 (C=C phenyl), 1489.68 (-C-H), 1430.59 (C=C phenyl), 1259.11 (Si-C), 1132.30, 1081.01, 1026.41 (Si-O), 997.01 (C-H phenyl).

^{1}H NMR

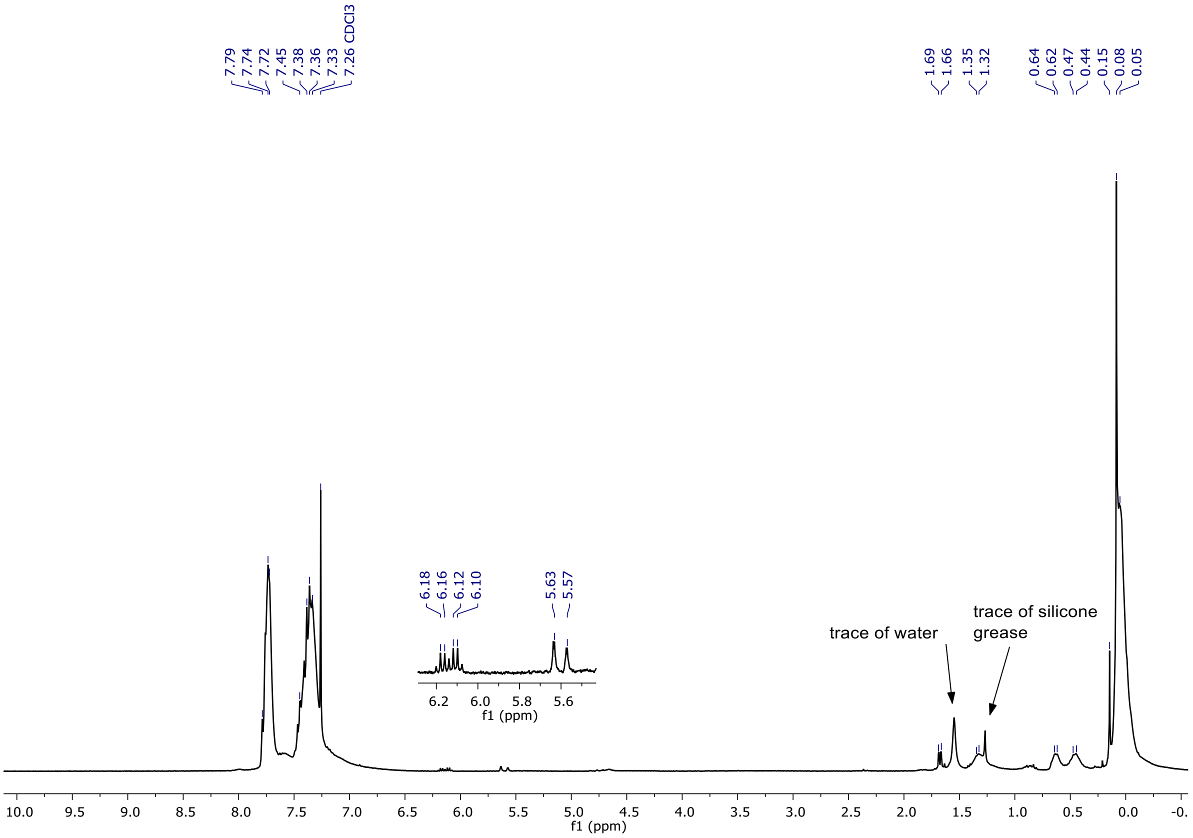

^{13}C NMR

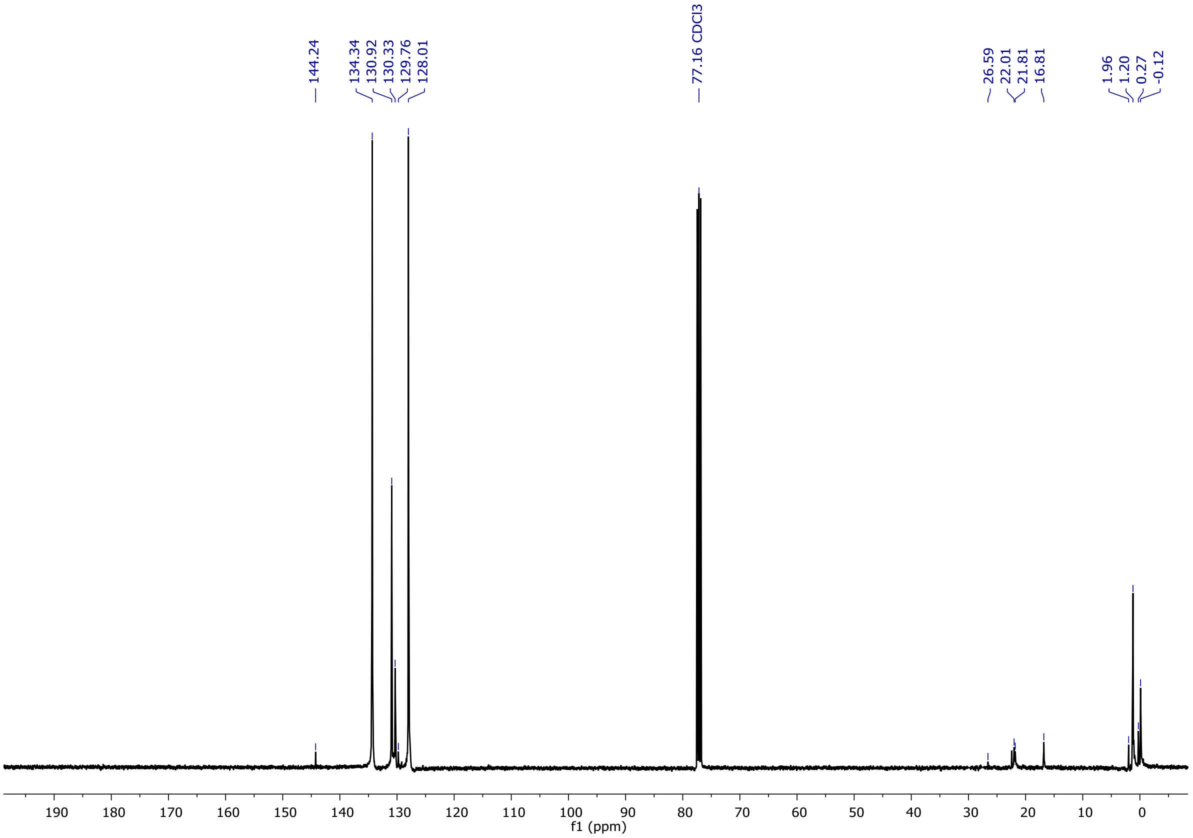

^{29}Si NMR

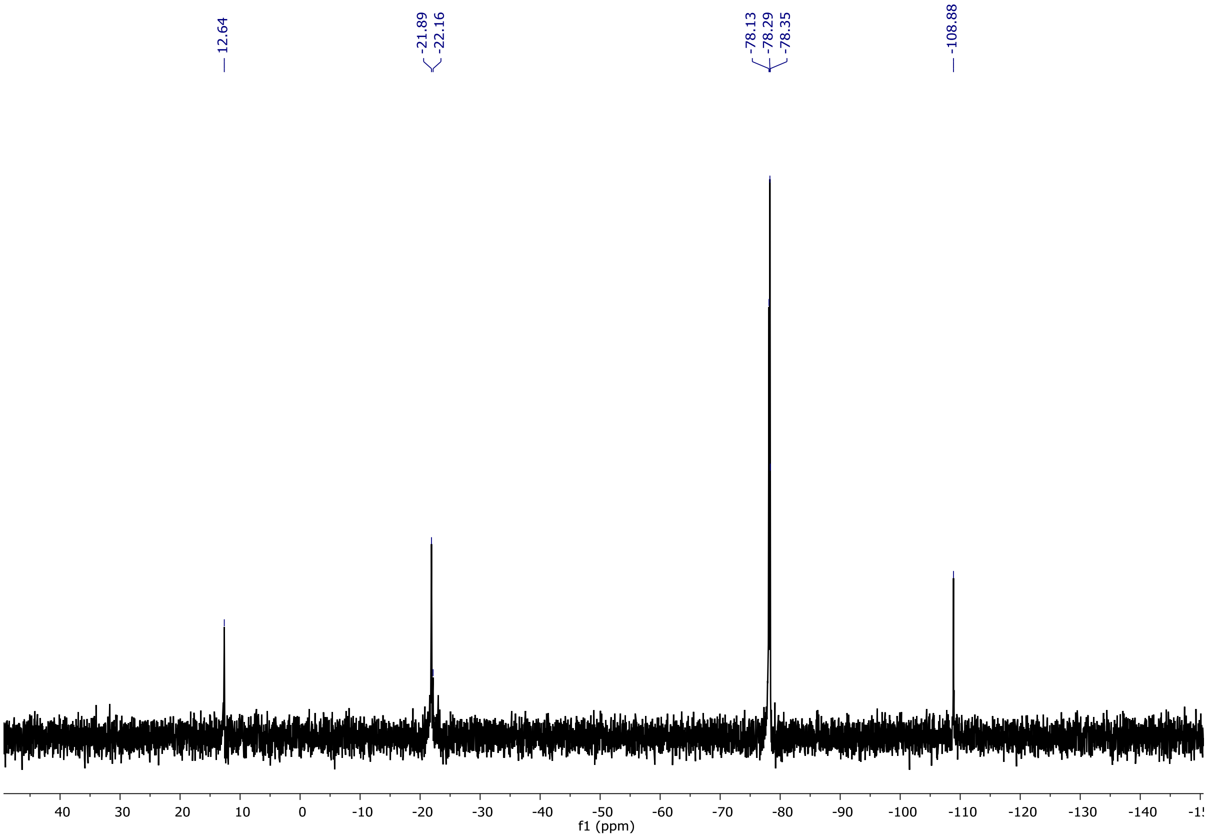

3-PhT$_8$@PS

^{1}H NMR (300.2 MHz, CDCl$_3$, δ, ppm): -0.12-0.14 (m, -SiC***H$_3$***), 0.43-0.49, 0.61-0.66, 1.33-1.33 (m, -C***H$_2$***-), 1.66-1.68, 5.57-5.63 and 6.09-6.17 (m, -C***H$_2$***-C***H***=C***H***- from dehydrogenative silylation by-product), 7.36-7.44, 7.72-7.74 (m, **Ph**). **^{13}C NMR** (100.6 MHz, CDCl$_3$, δ, ppm): -0.14, 1.20, 1.95 (-Si***C***H$_3$), 16.81, 21.80, 22.43 (-***C***H$_2$-), 128.00, 130.34, 130.91, 134.34 (Ph), 129.76 and 144.23 (-***C***H=***C***H- from dehydrogenative silylation by-product). **^{29}Si NMR** (79.5 MHz, CDCl$_3$, δ, ppm): 12.65 (-***Si***-CH$_2$-CH$_2$-CH$_2$-Si-), -21.90, -23.00 (-***Si***CH$_3$), -78.16, -78.30, -78.36, 108.90 (-***Si***O$_4$).
FT-IR (cm^{-1}): 3073.82, 3052.39 (C-H phenyl), 2961.13, 2920.27 (-C-H), 1594.49 (C=C phenyl), 1430.83 (C=C phenyl), 1259.33 (Si-C), 1134.50, 1091.58, 1018.79 (Si-O).

^{1}H NMR

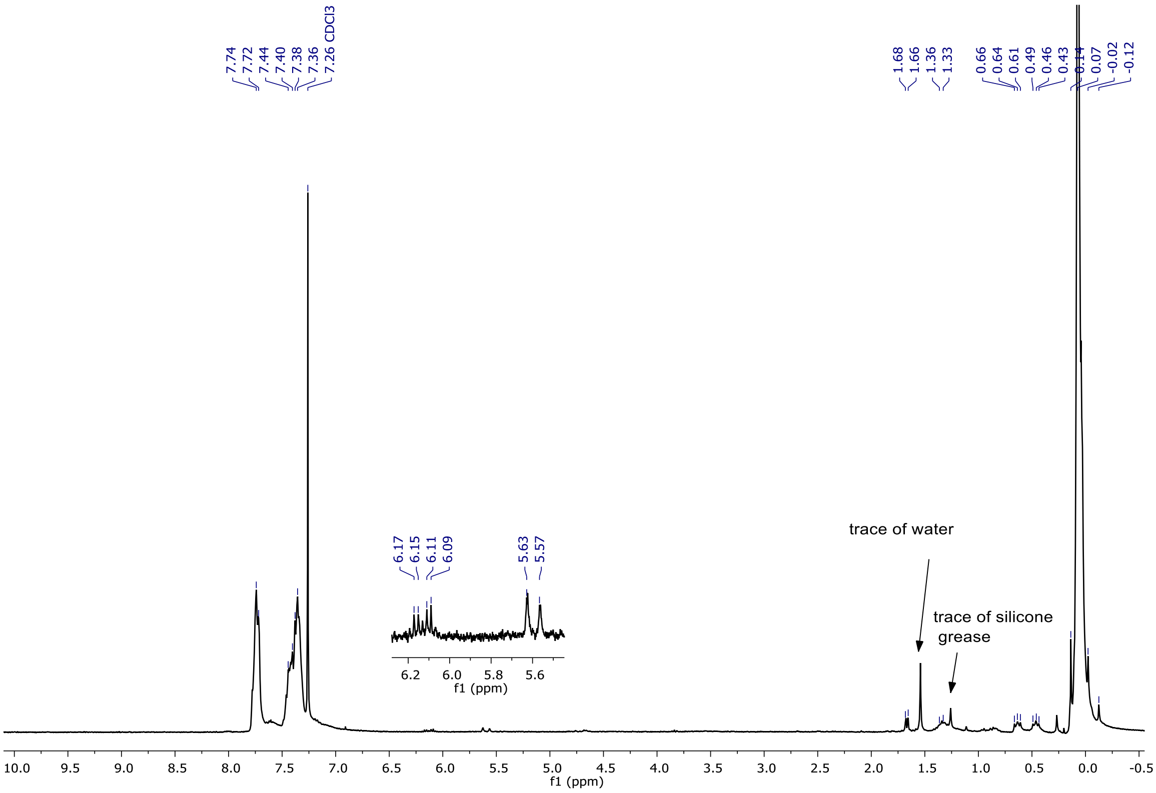

^{13}C NMR

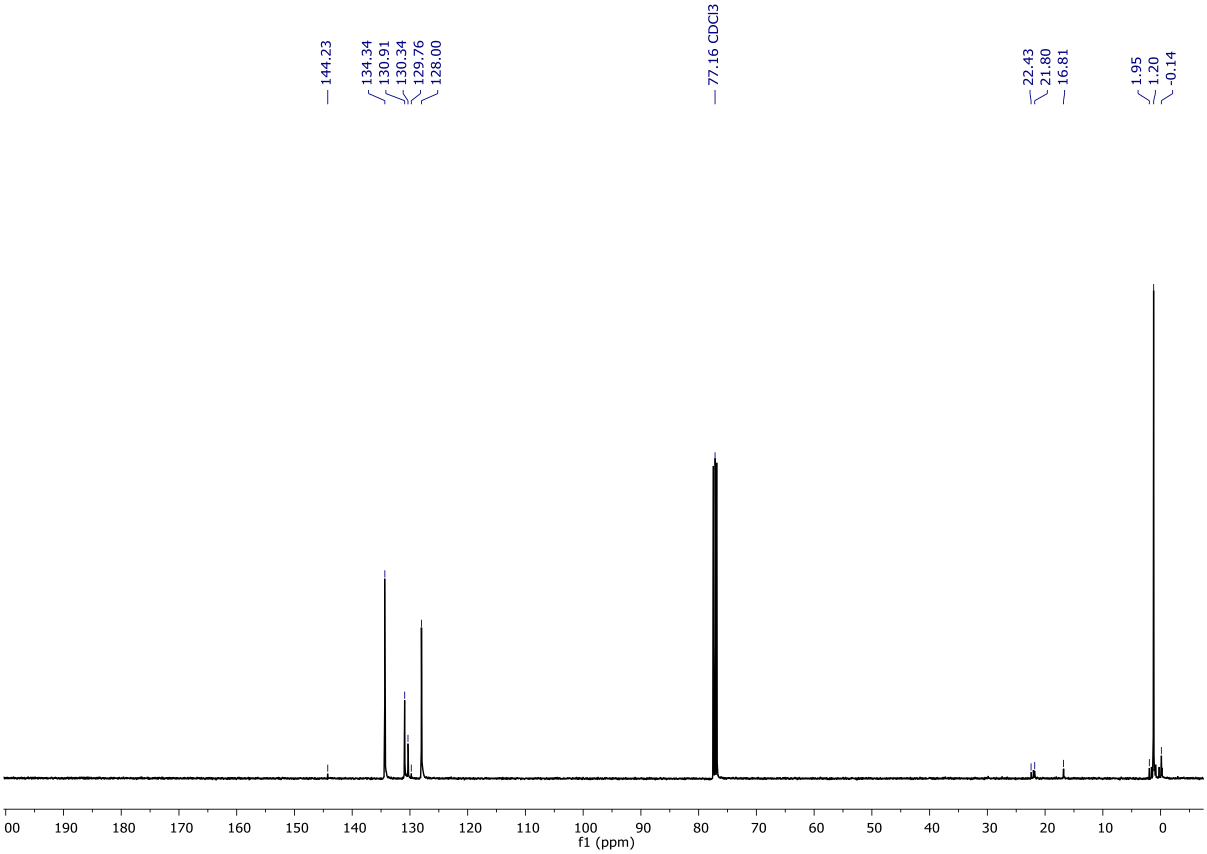

^{29}Si NMR

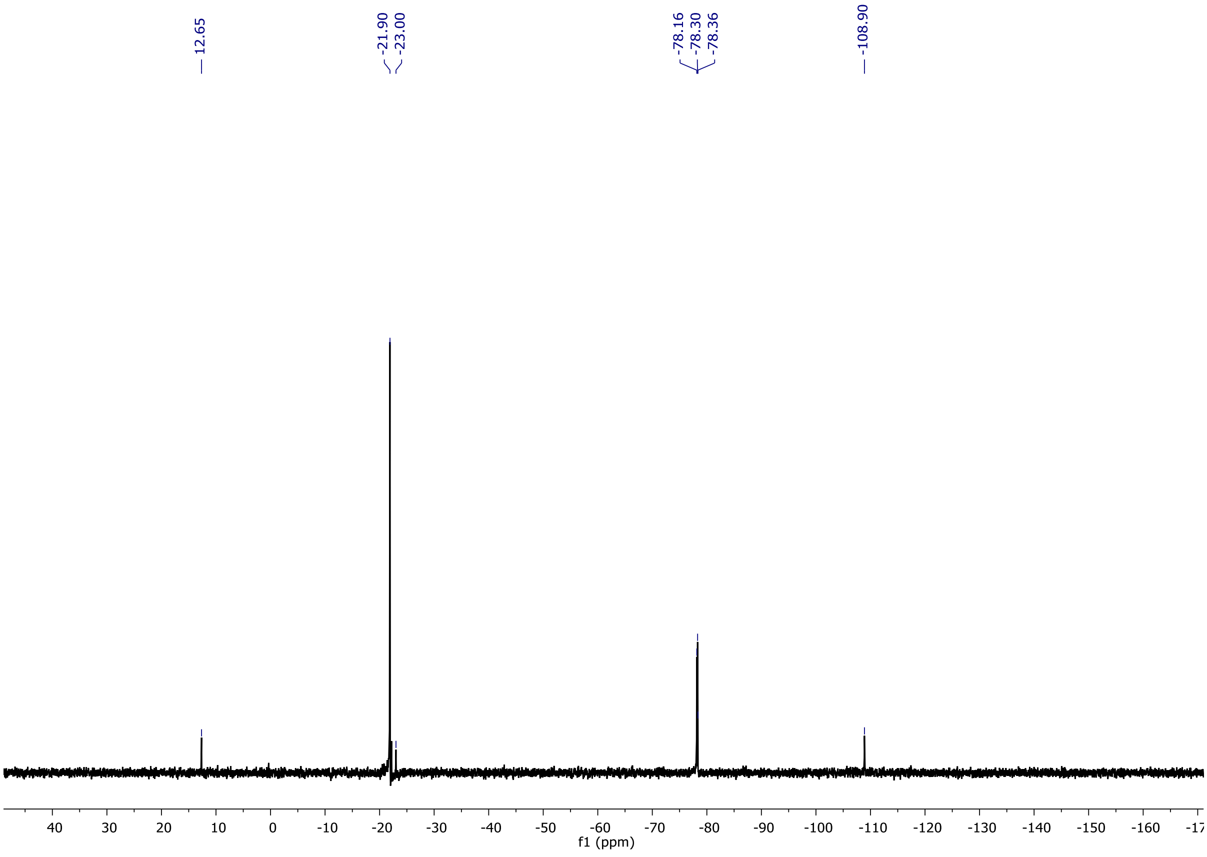

4-PhT$_8$@PS

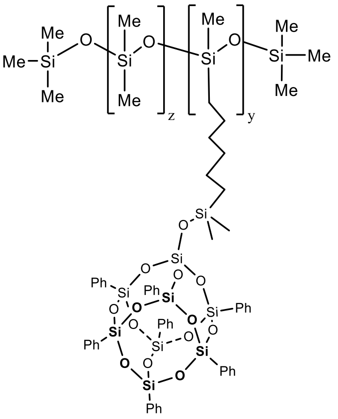

4-PhT$_8$@PS1

^{1}H NMR (300.2 MHz, $CDCl_3$, δ, ppm): 0.09 (m, -SiC***H₃***), 0.46-0.54, 1.17-1.27, 1.56-1.59 (m, -C$\boldsymbol{H_2}$-), 4.71 (s, -Si-***H***), 5.30 (m, -C***H***=C***H***- from by-product of bond isomerization), 7.37-7.45, 7.74-7.79 (m, **Ph**). **^{13}C NMR** (100.6 MHz, $CDCl_3$, δ, ppm): -0.19, 1.21, 1.95 (-Si***C***H_3), 17.70, 17.97, 23.12, 23.20, 33.23, 33.34 (-***C***H_2-), 128.00, 130.32, 130.91, 134.33 (Ph), 124.97 and 130.65 (-***C***H=***C***H- from by-product of bond isomerization). **^{29}Si NMR** (79.5 MHz, $CDCl_3$, δ, ppm): 13.12 (-***Si***-$(CH_2)_6$-Si-), -21.87, -22.15, -22.25 (-***Si***CH_3), -78.12, -78.26, -78.32, 108.95 (-***Si***O_4).

FT-IR (cm^{-1}): 3073.46, 3051.35 (C-H phenyl), 2959.22, 2921.80 (-C-H), 1594.38 (C=C phenyl), 1489.81 (-C-H), 1430.55 (C=C phenyl), 1258.86 (Si-C), 1131.73, 1076.04, 1026.74 (Si-O), 997.73 (C-H phenyl).

^{1}H NMR

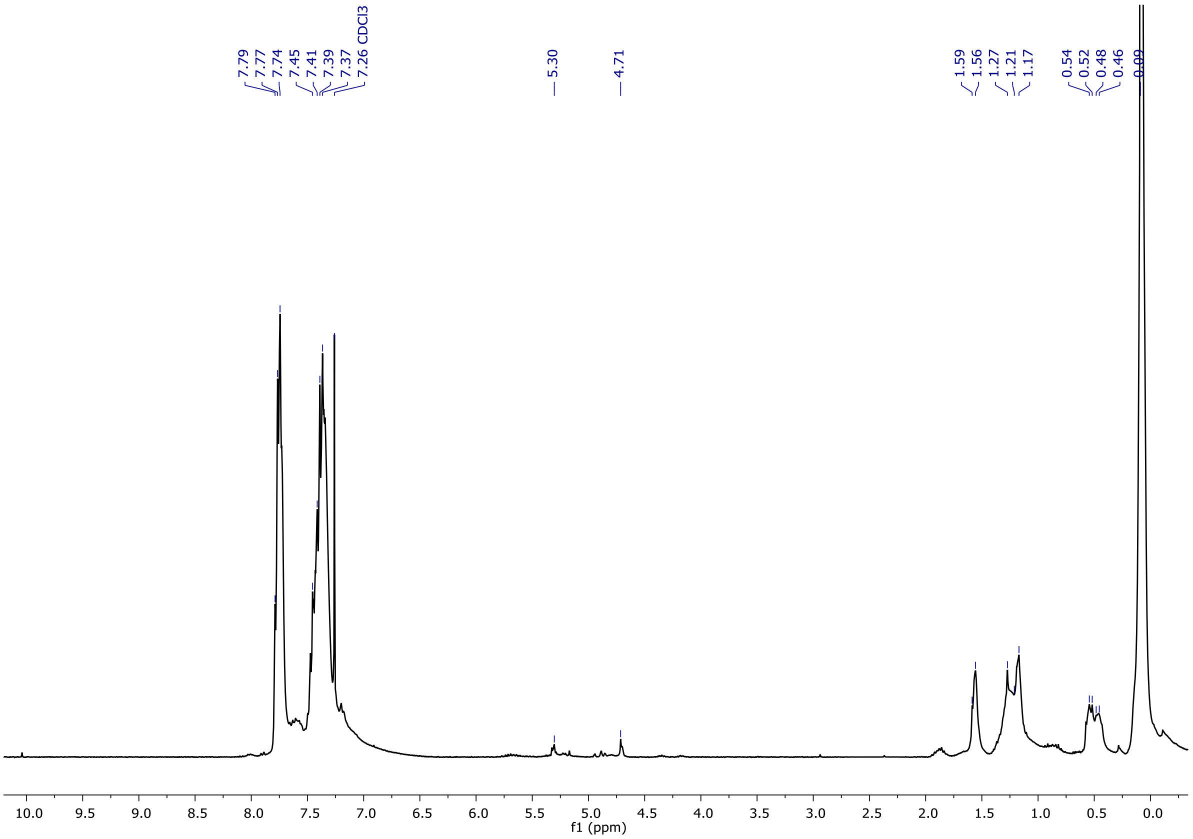

^{13}C NMR

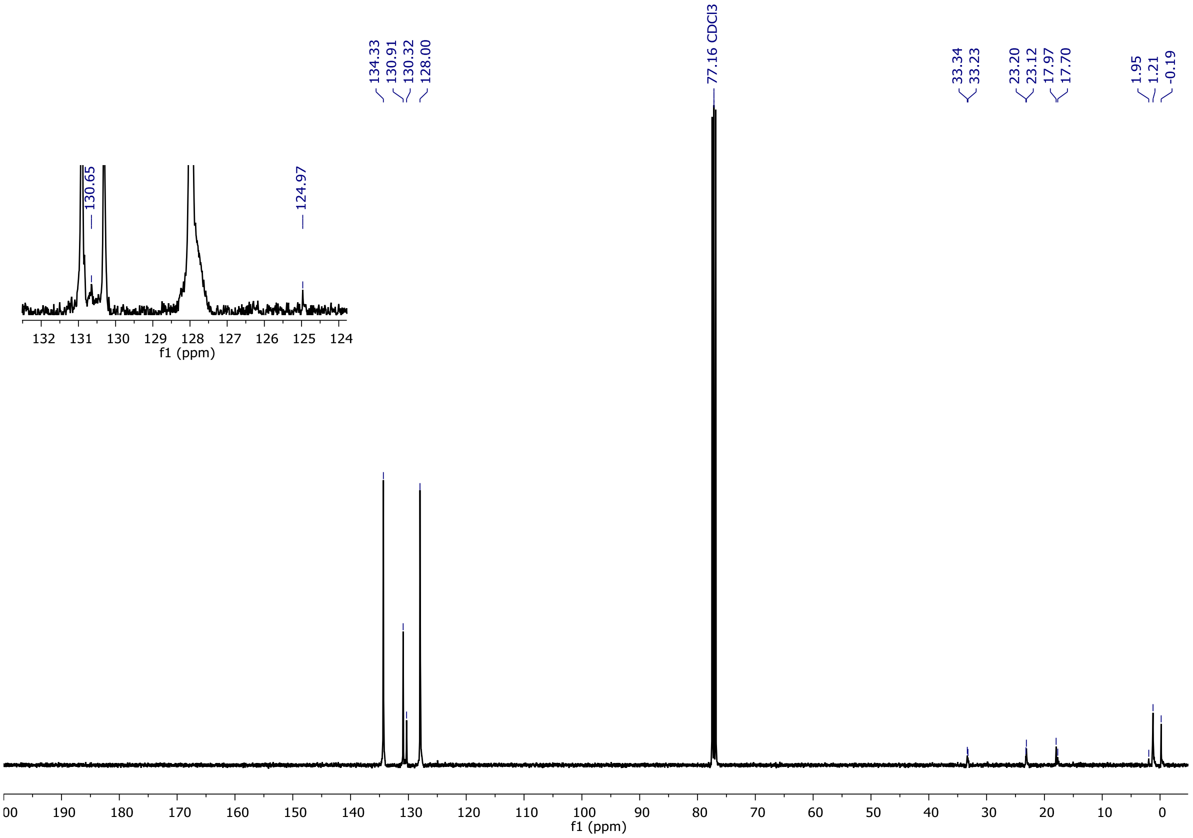

^{29}Si NMR

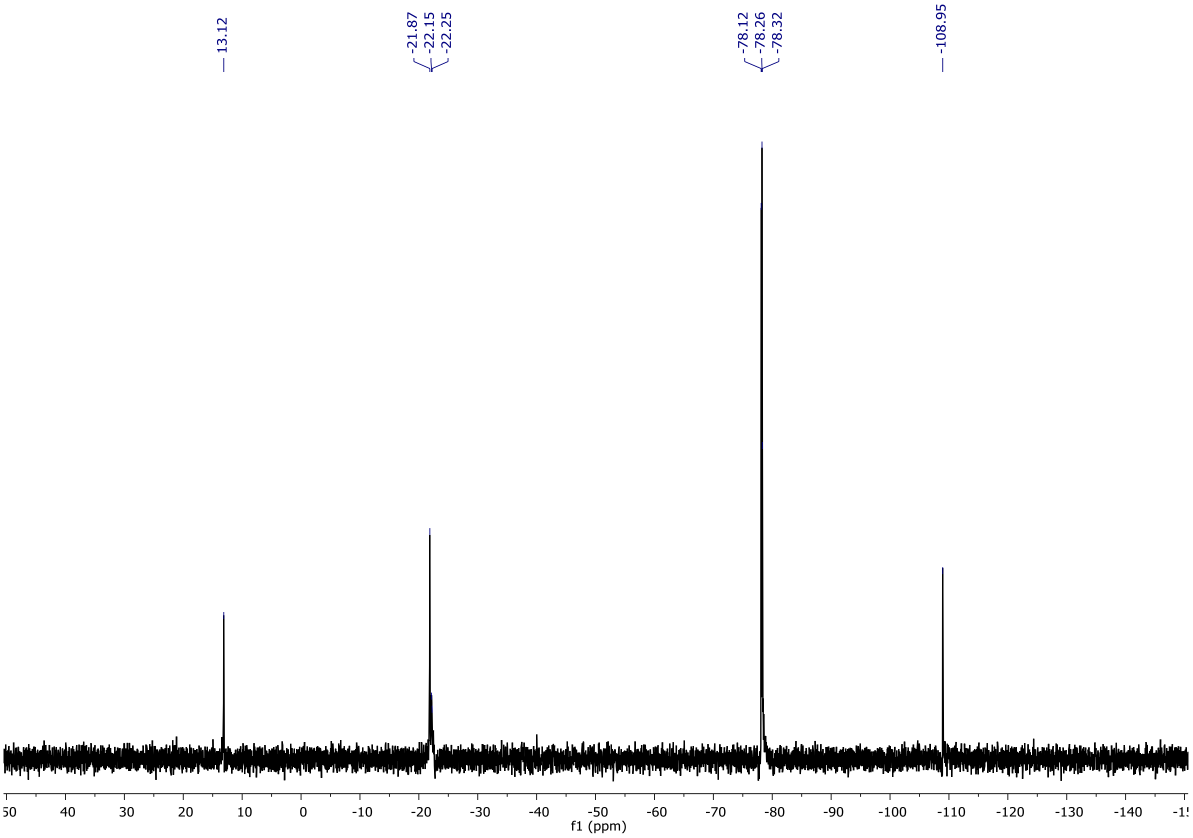

4-PhT$_8$@PS2

^{1}H NMR (300.2 MHz, $CDCl_3$, δ, ppm): 0.09 (m, -SiC***H$_3$***), 0.43-0.57, 1.18-1.27, 1.52-1.58 (m, -C***H$_2$***-), 4.70 (s, -Si-***H***), 5.30-5.32 (m, -C***H***=C***H***- from by-product of bond isomerization), 7.36-7.45, 7.74-7.78 (m, **Ph**). **^{13}C NMR** (100.6 MHz, $CDCl_3$, δ, ppm): -0.20, 1.19, 1.94 (-Si***C***H$_3$), 17.51, 17.68, 17.97, 18.04, 23.12, 23.20, 33.21, 33.34 (-***C***H$_2$-), 128.00, 130.33, 130.91, 134.35 (Ph), 124.96 and 130.40 (-***C***H=***C***H- from by-product of bond isomerization). **^{29}Si NMR** (79.5 MHz, $CDCl_3$, δ, ppm): 13.07 (-***Si***-$(CH_2)_6$-Si-), -21.92, -22.21 (-***Si***CH_3), -78.18, -78.32, -78.38, 109.00 (-***Si***O_4).
FT-IR (cm^{-1}): 3073.56, 3051.52 (C-H phenyl), 2960.80, 2923.08 (-C-H), 1594.29 (C=C phenyl), 1430.64 (C=C phenyl), 1258.86 (Si-C), 1133.70, 1087.75, 1015.68 (Si-O).

^{1}H NMR

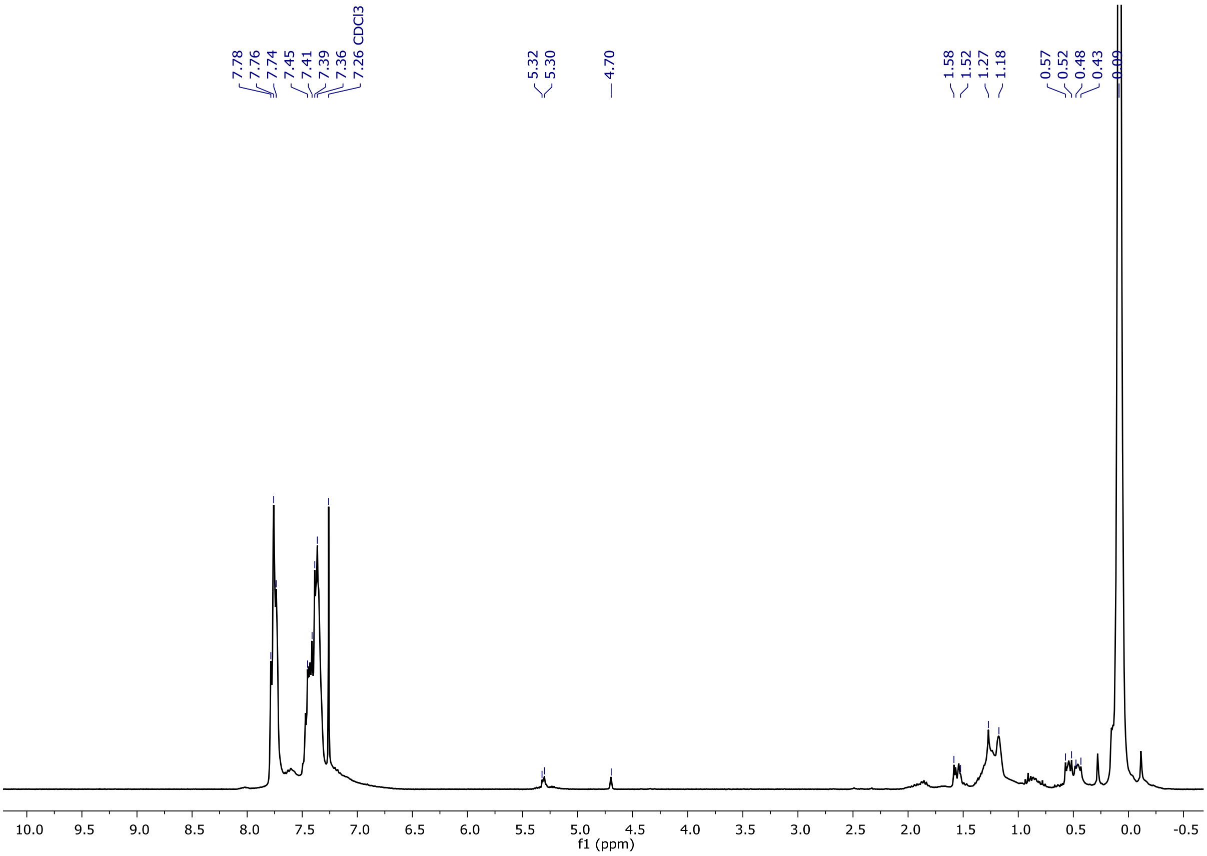

^{13}C NMR

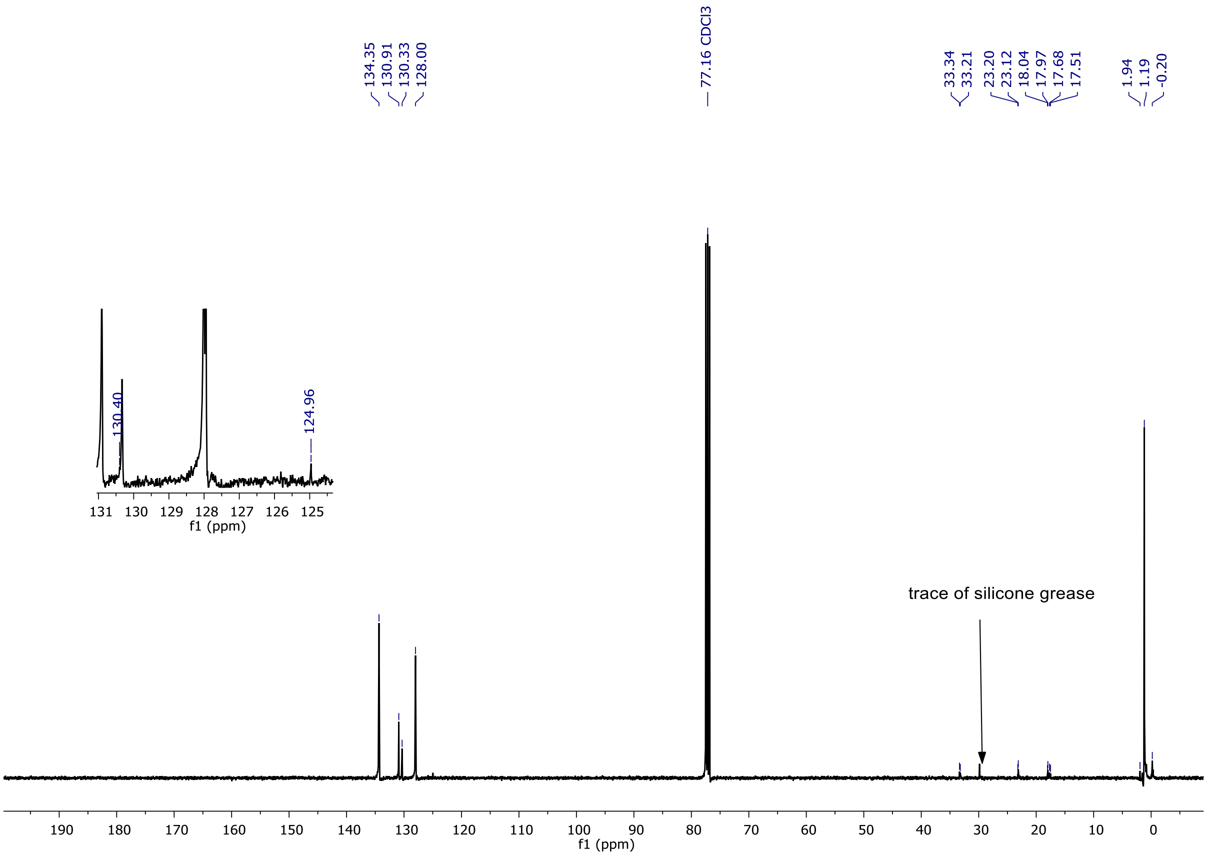

^{29}Si NMR

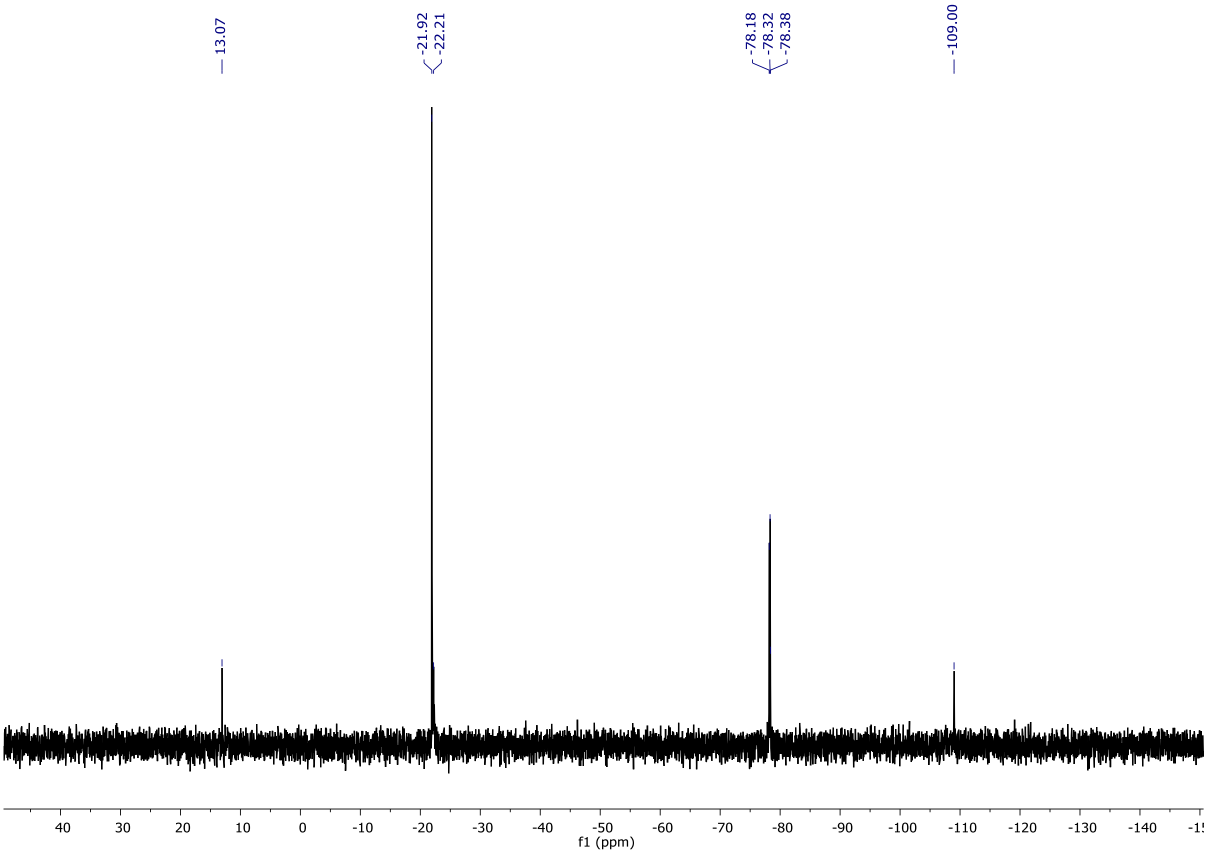

5-PhT$_8$@PS

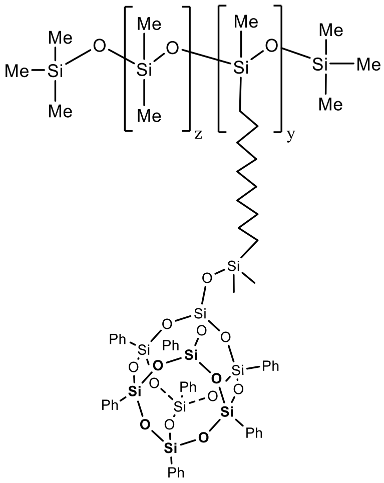

5-PhT$_8$@PS1

^{1}H NMR (300.2 MHz, CDCl$_3$, δ, ppm): 0.08 (m, -SiC***H$_3$***), 0.51-0.54, 1.16-1.27 (m, -C***H$_2$***-), 4.70 (m, -Si-***H***), 5.39 (m, -C***H***=C***H***- from by-product of bond isomerization), 7.36-7.47, 7.73-7.75 (m, **Ph**). **^{13}C NMR** (100.6 MHz, CDCl$_3$, δ, ppm): -0.15, 1.22, 1.96 (-Si***C***H$_3$), 17.51, 17.95, 23.17, 29.51, 29.93, 33.61 (-***C***H$_2$-), 128.00, 130.33, 130.91, 134.35 (Ph). **^{29}Si NMR** (79.5 MHz, CDCl$_3$, δ, ppm): 13.15 (-***Si***-(CH$_2$)$_{10}$-Si-), -21.87, -22.26 (-***Si***CH$_3$), -78.12, -78.26, -78.32, 108.94 (-***Si***O$_4$).
FT-IR (cm^{-1}): 3073.45, 3051.40 (C-H phenyl), 2922.46, 2852.81 (-C-H), 1594.37 (C=C phenyl), 1489.78 (-C-H), 1430.65 (C=C phenyl), 1259.11 (Si-C), 1132.85, 1091.30, 1028.59 (Si-O), 998.07 (C-H phenyl).

^{1}H NMR

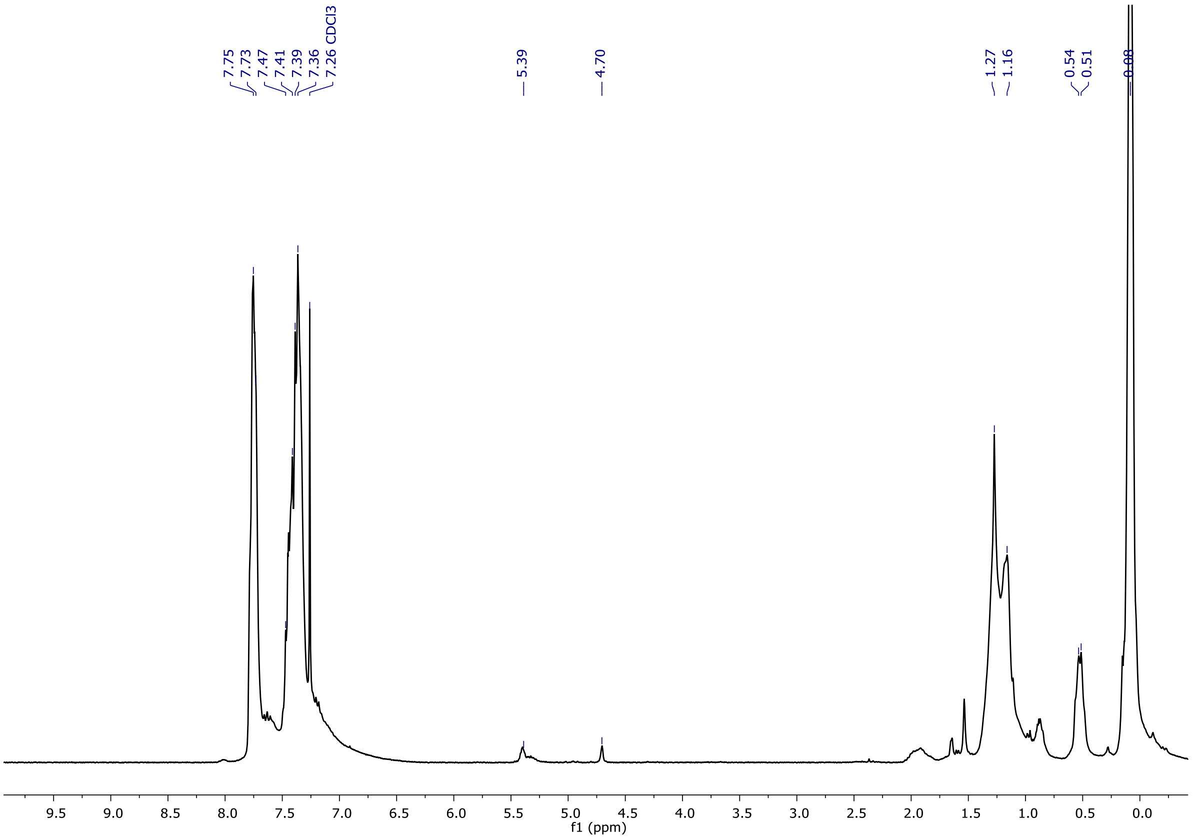

^{13}C NMR

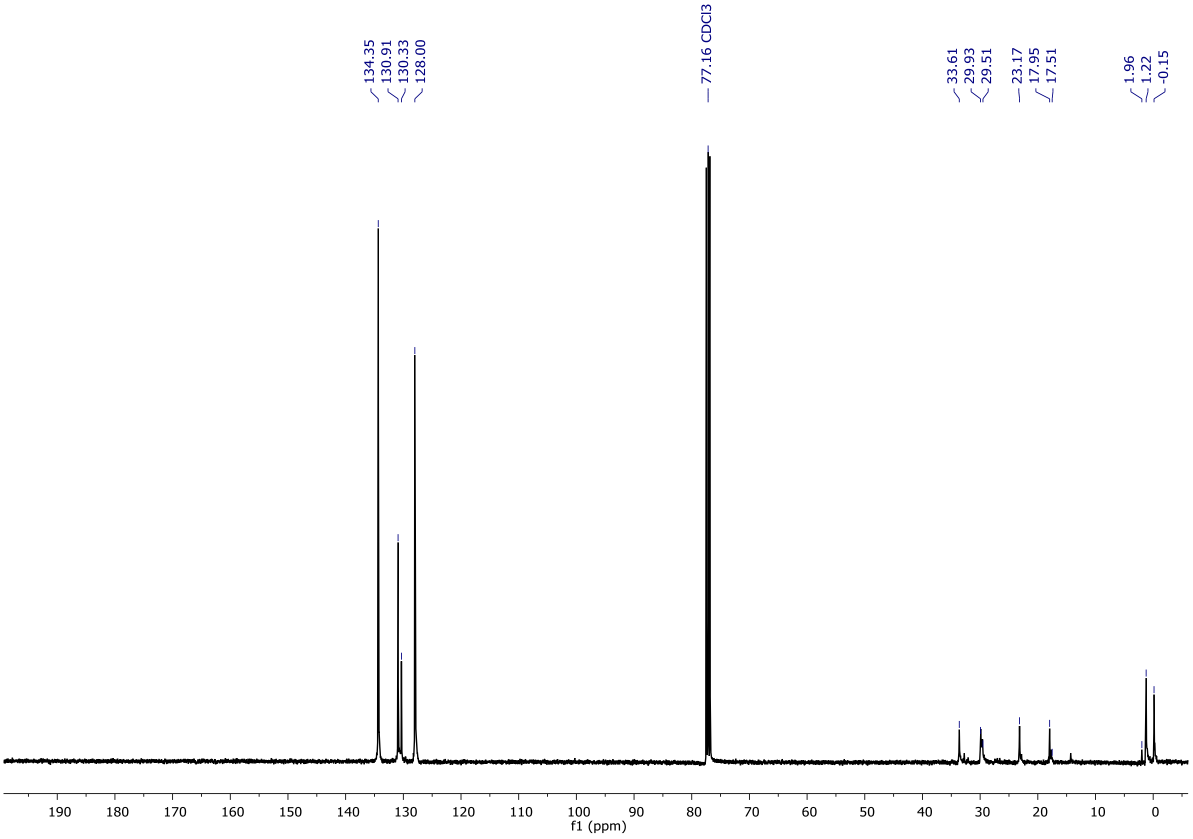

^{29}Si NMR

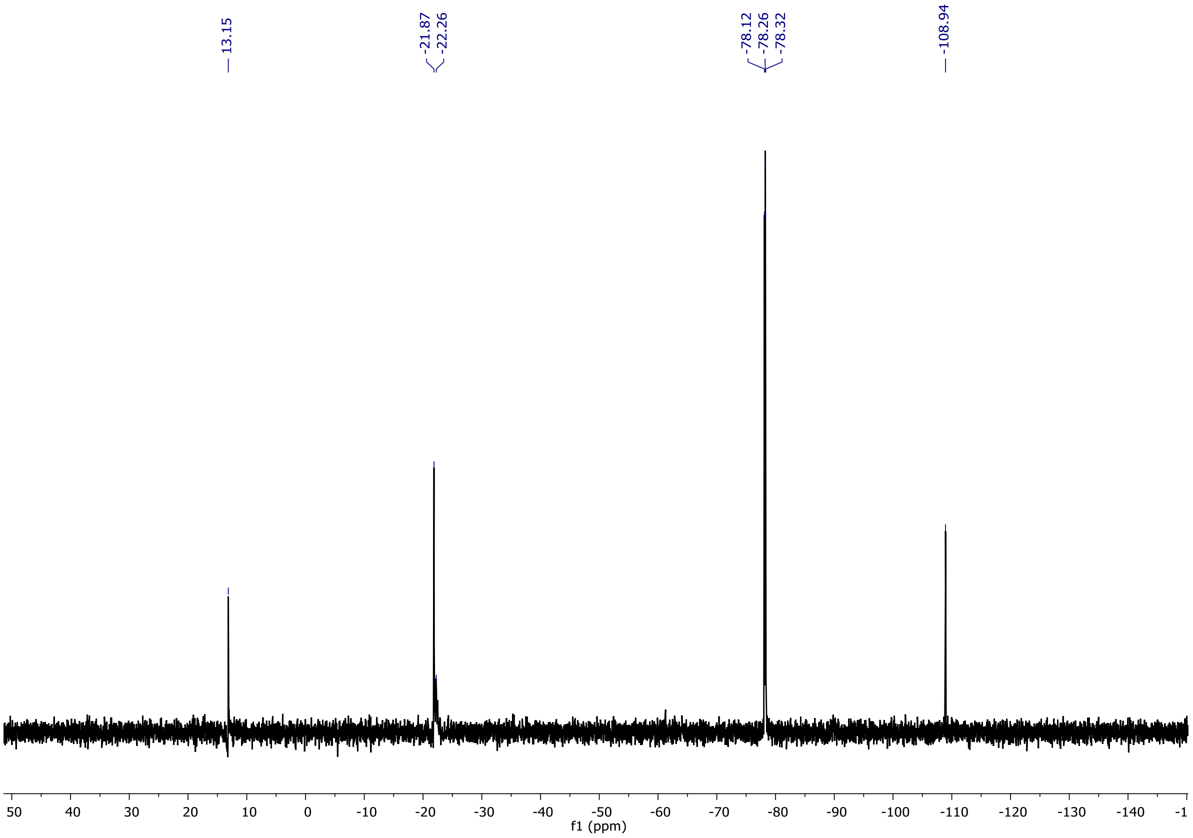

5-PhT$_8$@PS2

^{1}H NMR (300.2 MHz, $CDCl_3$, δ, ppm): 0.07-0.09 (m, -SiC***H$_3$***), 0.49-0.56, 1.16-1.28 (m, -C***H$_2$***-), 4.70 (m, -Si-***H***), 5.40 (m, -C***H***=C***H***- from by-product of bond isomerization), 7.36-7.46, 7.74-7.79 (m, **Ph**). **^{13}C NMR** (100.6 MHz, $CDCl_3$, δ, ppm): -0.15, 1.20 (-Si***C***H$_3$), 17.71, 17.96, 23.17, 29.31, 29.59, 29.68, 29.92, 32.75, 33.61 (-***C***H$_2$-), 127.96, 128.00, 130.36, 130.91, 134.36 (Ph). **^{29}Si NMR** (79.5 MHz, $CDCl_3$, δ, ppm): 13.10 (-***Si***-$(CH_2)_{10}$-Si-), -21.96, -22.21, -22.27 (-***Si***CH$_3$), -78.19, -78.33, -78.39, 109.00 (-***Si***O$_4$). **FT-IR** (cm^{-1}): 3073.56, 3052.28 (C-H phenyl), 2960.62, 2823.07 (-C-H), 1594.24 (C=C phenyl), 1489.73 (-C-H), 1430.70 (C=C phenyl), 1259.28 (Si-C), 1133.79, 1087.68, 1015.09 0(Si-O), 996.95 (C-H phenyl).

^{1}H NMR

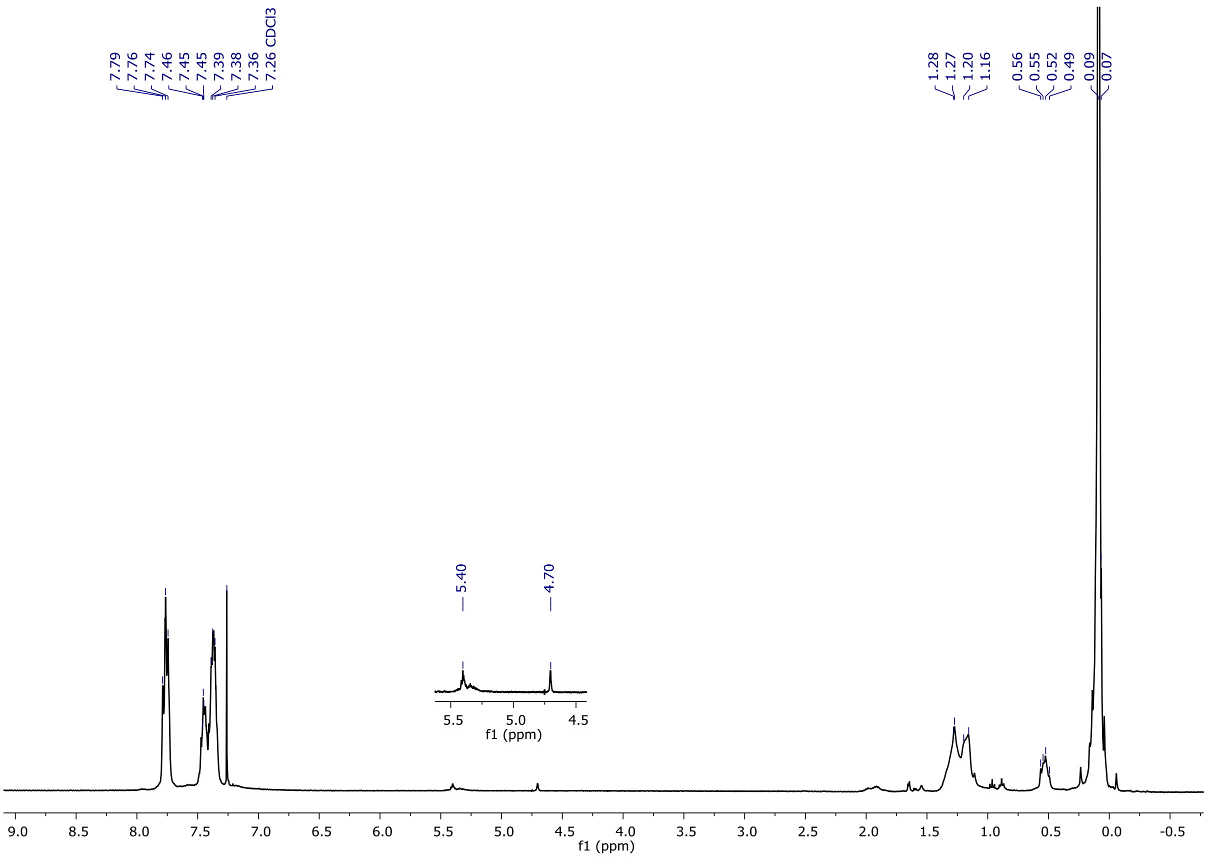

^{13}C NMR

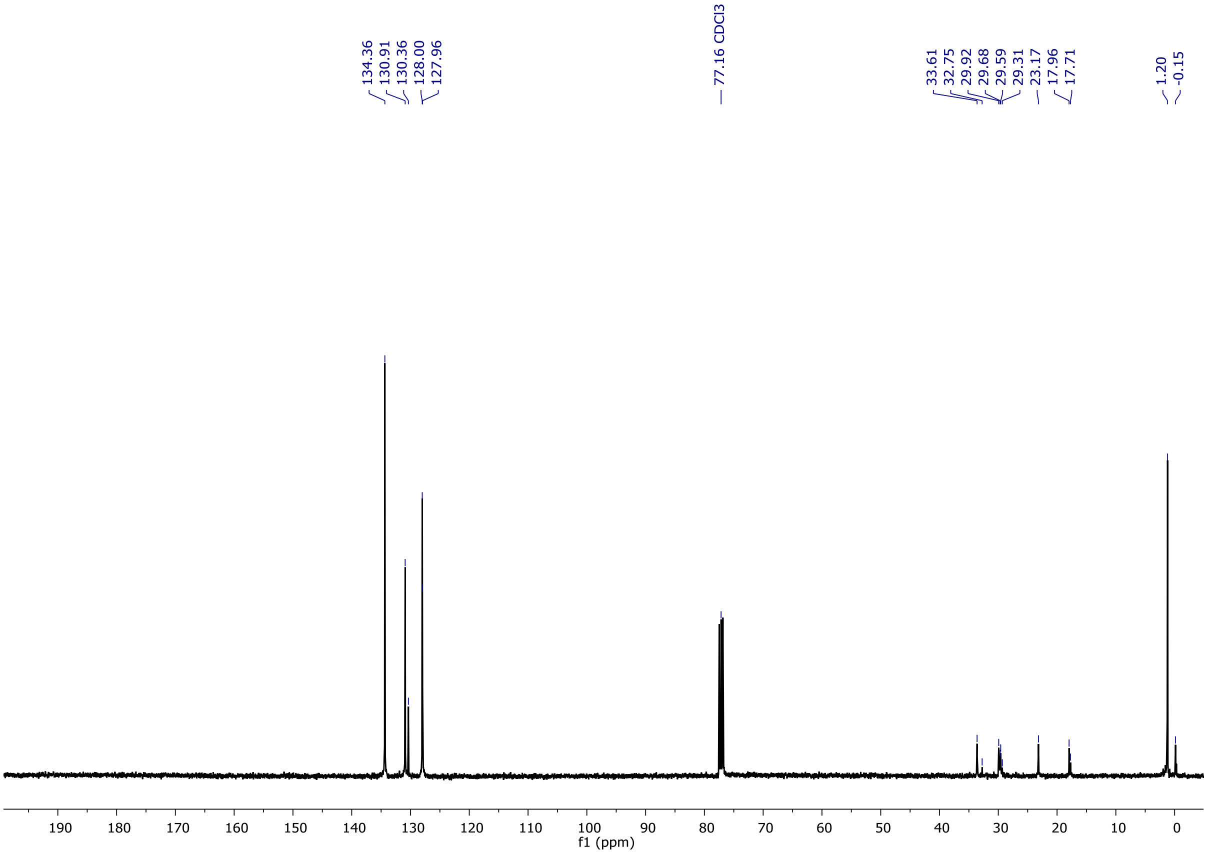

^{29}Si NMR

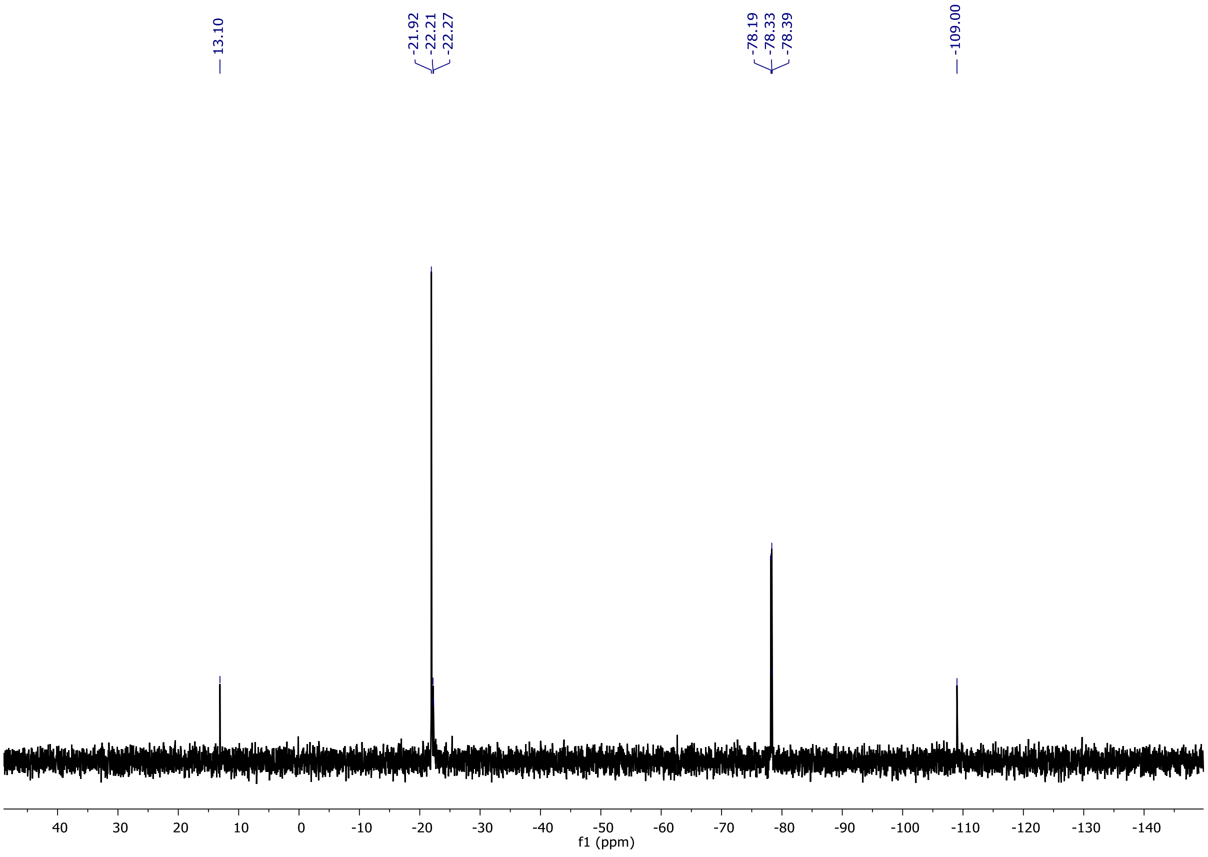

3. Stacked FT-IR spectra of starting material (PS2), substrates and the resulting products

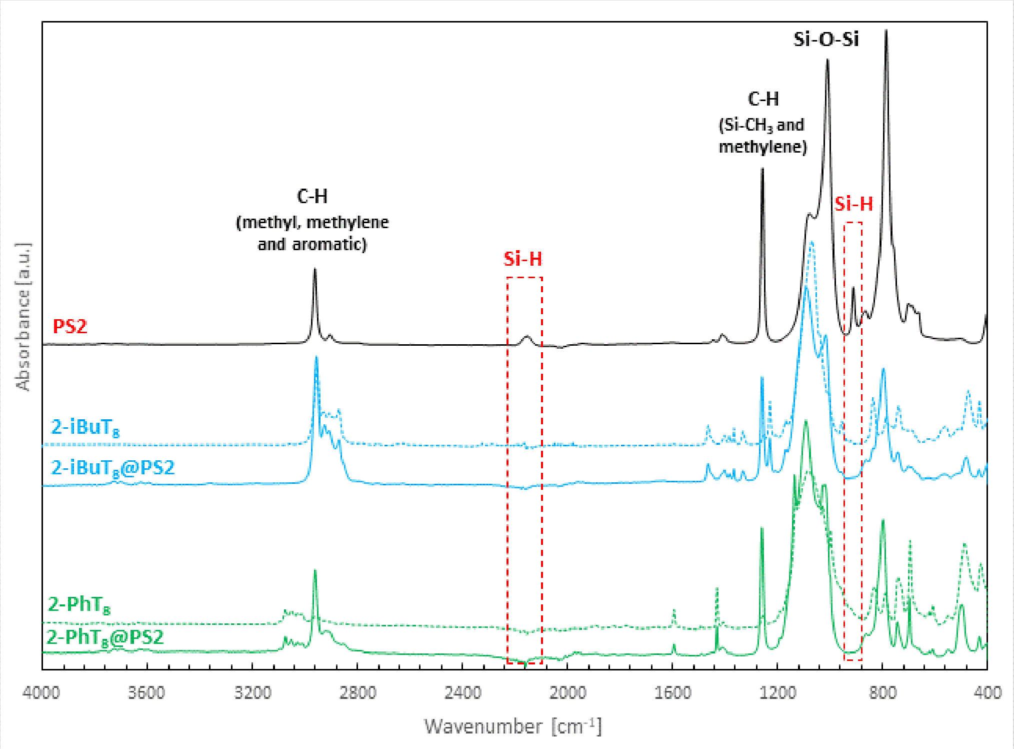

Figure S1. Selected FT-IR spectra of polysiloxane PS2 and mono(vinyl)substituted iBuT$_8$ (2-iBuT$_8$), and mono(vinyl)substituted PhT$_8$ (2-PhT$_8$) along with the obtained products 2-iBu/PhT$_8$@PS2.

4. References

1. Hou, Z.; Yang, B.; Zhang, D.; Xu, Z.; Kan, C. Polysiloxanes with Quaternary Ammonium and Polyether Groups for Silyl-Terminated Polypropylene Oxide Waterborne Emulsions. *J. Surfactants Deterg.* **2016**, *19*, 739–745, doi:10.1007/s11743-016-1825-8.
2. Guerra-Contreras, A.; Villegas, A.; Ramírez-Oliva, E.; Cervantes, J. Characterization and Study of Properties in a Polar Solvent of a Functionalized and Quaternized Poly(dimethylsiloxane-co-methyl-hydridosiloxane). *Silicon* **2017**, *9*, 525–533, doi:10.1007/s12633-015-9286-7.
3. Chung, D. won; Lim, J.C. Study on the effect of structure of polydimethylsiloxane grafted with polyethyleneoxide on surface activities. *Colloids Surfaces A Physicochem. Eng. Asp.* **2009**, *336*, 35–40, doi:10.1016/j.colsurfa.2008.11.020.
